# Chronic Fructose Substitution for Glucose or Sucrose in Food or Beverages and Metabolic Outcomes: An Updated Systematic Review and Meta-Analysis

**DOI:** 10.3389/fnut.2021.647600

**Published:** 2021-04-28

**Authors:** Mohammad Ishraq Zafar, Michael Frese, Kerry E. Mills

**Affiliations:** ^1^Institute of Reproductive Health/Center of Reproductive Medicine, Tongji Medical College, Huazhong University of Science and Technology, Wuhan, China; ^2^Faculty of Science and Technology, University of Canberra, Canberra, ACT, Australia

**Keywords:** fructose, glucose, sucrose, meta-analysis, glycemia

## Abstract

Despite the publication of several of meta-analyses in recent years, the effects of fructose on human health remains a topic of debate. We previously undertook two meta-analyses on post-prandial and chronic responses to isoenergetic replacement of fructose for sucrose or glucose in food or beverages (Evans et al. 2017, AJCN 106:506–518 & 519–529). Here we report on the results of an updated search with a complete re-extraction of previously identified studies and a new and more detailed subgroup-analysis and meta-regression. We identified two studies that were published after our previous analyses, which slightly altered effect sizes and conclusions. Overall, the isoenergetic substitution of fructose for glucose resulted in a statistically significant but clinically irrelevant reduction in fasting blood glucose, insulin, and triglyceride concentrations. A subgroup analysis by diabetes status revealed much larger reductions in fasting blood glucose in people with impaired glucose tolerance and type 2 diabetes. However, each of these subgroups contained only a single study. In people with a healthy body mass index, fructose consumption was associated with statistically significant, but clinically irrelevant reductions in fasting blood glucose and fasting blood insulin. Meta-regression of the outcomes by a number of pre-identified and *post-hoc* covariates revealed some sources of heterogeneity, such as year of publication, age of the participants at baseline, and participants' sex. However, the small number of studies and the large number of potential covariates precluded detailed investigations of effect sizes in different subpopulations. For example, well-controlled, high quality studies in people with impaired glucose tolerance and type 2 diabetes are still lacking. Taken together, the available data suggest that chronic consumption of fructose is neither more beneficial, nor more harmful than equivalent doses of sucrose or glucose for glycemic and other metabolic outcomes.

## Introduction

Historically, as reviewed by Sievenpiper (1), fructose was considered a healthy choice for people with diabetes (1). For the last several decades, however, fructose has instead been seen as a primary driver of adverse health outcomes (2–12). In 2004, a retrospective observational correlation of increasing dietary intake of high fructose corn syrup (HFCS) with increasing obesity was published (13). Despite the findings being observational, and the authors' own analysis that HFCS “may play a role” in the increase in obesity due to a “temporal relation,” this analysis led to a large number of studies being done to investigate not if, but how fructose causes harm, even though HFCS contains, at most, only 55% fructose. The recent publication of several meta-analyses demonstrating neutral or even positive effects of isoenergetic fructose consumption, both for short-term and chronic exposures (14–23), have not, despite significant media coverage (24–30), changed the views of many in the scientific community and the general public. This is perhaps because the epidemiological trials, real-world interventional trials, and isoenergetic randomized controlled trials are addressing different questions.

Our previous meta-analyses were the first to concentrate on potential “real world” changes in the use of fructose, i.e., the isoenergetic substitution of fructose for current uses of sucrose or glucose in food or beverages (14, 15). In addition, these analyses were restricted to studies that used double-blind methodology, provided participants with all foods, and/or kept a detailed analysis of the participants' food intakes. This allowed us to be more confident in the interpretation of the results. For example, we had fewer concerns that participants altered their behavior based on the knowledge of their allocation. Our previous analysis of post-prandial studies included 47 studies reporting on 62 individual study arms (14). The data overwhelmingly showed that fructose reduces post-prandial peak blood glucose, especially in people with overweight or obesity, and in people with impaired glucose tolerance, type 1 diabetes, and type 2 diabetes. Other changes were significant reductions in the post-prandial blood glucose area under the curve (AUC) and peak post-prandial insulin concentrations. No changes were observed in peak post-prandial triglyceride concentrations (14). Our acute and chronic findings gained some traction, at least in the public domain (24–30). However, several narrative reviews that were published well after our meta-analyses, failed to mention that, at least for the outcomes mentioned here, fructose does not seem to be a specific cause of disease above and beyond that of other sugars (31–36).

Given that over 4 years had elapsed since our previous search, and that the debate over the role of fructose in health is still ongoing, we undertook an updated search for chronic studies matching our previous search criteria.

## Methods

### Study Design

This update followed the Preferred Reporting Items for Systematic Reviews and Meta-Analyses (PRISMA) guidelines (37). The PICOTS question was: in people with normal glucose tolerance, impaired glucose tolerance, or diabetes, with healthy body weight, overweight, or obesity, does fructose, isoenergetically substituted for sucrose or glucose in food or beverages, alter measures of longer-term glycemic control [glycated hemoglobin (HbA1c), homeostatic model assessment (HOMA), fasting blood glucose, fasting blood insulin], blood lipids [fasting total cholesterol, low density lipoproteins (LDL), high density lipoproteins (HDL), and triglycerides], and obesity (measured as body weight), over a period of two or more weeks in a double-blind, food-controlled, or strict dietary analysis setting?

### Participants

Participants in the studies could be children, teenagers, or adults, with normal glucose tolerance, impaired glucose tolerance, or diabetes. People with healthy body weight, or with overweight, or obesity were included. No restrictions were placed on ethnicity of the participants, or the country in which the study took place.

### Interventions

Included interventions were purified fructose (i.e., not fructose-containing foods such as fruits), provided to participants in either foods (e.g., baked into cakes, dissolved in jams or yogurts) or beverages.

### Comparators

Acceptable comparators were purified glucose or sucrose provided to participants in the same vehicle and at the same caloric value as fructose.

### Systematic Review Protocol

We followed the same search strategy, inclusion and exclusion criteria, and largely the same subgroup analyses outlined in the original protocol registered previously (38). The PROSPERO registration number of the present study is CRD42015029385.

### Data Sources

The search terms from the original analysis were reused for the updated search (15). The Cochrane Library, MEDLINE, EMBASE, the World Health Organization (WHO) International Clinical Trials Registry and clinicaltrials.gov databases were searched. The search was restricted to the time frame from the day before the date of the previous search (April 26, 2016) until September 23, 2020; no other restrictions were applied. All citations were uploaded into Covidence (39). Duplicates were removed and the remaining studies were subjected to double-blind coding at the title and abstract level; conflicts were resolved by consensus. The remaining studies were obtained as full texts and subjected to double-blind coding for inclusion in the review; again, any conflicts were resolved by consensus.

### Study Selection and Data Extraction

The following selection criteria were applied to each citation and full text: randomized controlled trials in humans of at least 2 weeks' duration that compared fructose with either sucrose or glucose; the study was double-blind or blinded to participants; the diet was monitored or provided, or both; and data on any blood glucose outcome were provided. The studies could include people with or without diabetes, but not people who were acutely ill. Studies were excluded if they were of <2 weeks in duration, were unblinded or the diet was not isoenergetic (demonstrated through monitoring of the diet or providing all participants with their food), or if blood glucose data were not reported.

Data from all included studies (previously included studies and new studies) were extracted into an Excel spreadsheet by one author and checked by another. We extracted data on study characteristics (study type, substituted sugar, age, weight, diabetes status of participants etc.) along with changes in HbA1c, HOMA, fasting blood glucose, fasting blood insulin, fasting total, HDL and LDL cholesterol, fasting triglycerides, and body weight.

The definitions of normoglycemia, impaired glucose tolerance, and type 2 diabetes were taken from General Practice Management of Diabetes (40). If stated, we used the study authors' baseline values and classification of their study population. If this information was not provided, fasting blood glucose values were defined as the mean blood glucose value at time 0 of the intervention.

### Data Analysis

Data presented in different units (e.g., μIU/mL, pmol/mL, or g/L for insulin concentrations) were standardized using EndMemo.com (41). When required, data were converted using the statistical algorithms reported by the Cochrane Collaboration (42). Where data were given as means and standard deviations (SD), these were converted to standard errors (SE), using the following formula:

$$\begin{array}{l} SD=SE\;\times \sqrt{N}, \end{array}$$

where *N* = the number of participants in the study arm. Where neither SD or SE was given, and could not be calculated by other means, the SE was imputed by taking the mean of the SEs from all other studies of the same kind reporting the same outcome.

Mean differences (MD) and standard errors (SE) of the mean differences were calculated for crossover studies as follows:

$$\begin{array}{l} \text{MD}={\text{Outcome}}_{\left(\text{end of intervention period}\right)}-{\text{Outcome}}_{\left(\text{end of control period}\right)} \end{array}$$

and the SE as:

$$\begin{array}{l} SE\;=\;\sqrt{\left[\left(SEendi^{2}\;+SEendc^{2}\right)-2r\left(SEendi\;\times \;SEendc\right)\right]}, \end{array}$$

where *r* is the intrapersonal correlation coefficient of the individual outcome, SE endi = the standard error of the end value of the intervention period, and SE endc = the standard error of the end value of the control period (14).

For parallel studies, the mean differences were calculated as follows:

$$\begin{array}{l} \text{MD}={\text{Outcome}}_{\left(\text{end of intervention period}\right)}-{\text{Outcome}}_{\left(\text{end of control period}\right)} \end{array}$$

and the SE as:

$$\begin{array}{l} SE\;=\;\sqrt{}\left(SE-endi^{2}+SE-endc^{2}\right), \end{array}$$

where SEendi = the standard error of the end value of the intervention arm, and SEendc = the standard error of the end value of the control arm.

Outcome data were copied into Review Manager 5.4 and analyzed using a generic inverse variance, random effects model with 95% confidence intervals (CI) (43). The use of this model was chosen in order to combine crossover and parallel trials. A random effects model was chosen over a fixed effects model, as a random effects model is the appropriate statistical model for combining studies that differ in the participant characteristics (e.g., age, body weight, dose of sugar, etc.). Most outcomes were reported as mean differences; standardized mean differences were used where studies reported outcomes in different ways that could not be converted to single scale.

Where a study had more than two arms, both arms were included in separate subgroups with full participant numbers for all study arms. However, in these cases, the totals were removed from the meta-analyses, and only subtotals were included. If a study was included in a single subgroup, the number of participants in the repeated study arm was halved to avoid double-counting (44). If studies gave data as medians and ranges, or medians and inter-quartile ranges (IQR), these were converted to means and standard deviations following the work of Luo et al. and Wan et al. (45, 46).

Some studies gave participants fructose, sucrose, or glucose as a percent of daily energy requirements rather than a specific dose. In these cases, the doses were calculated from the baseline data (weight, height, BMI), using national averages where required.

Subgroups analyses were undertaken to determine the effect of study design (crossover compared with parallel design), publication date, blinding, dose of sugar used, funding source, diabetes status, body weight, sex, age, and sugar presentation (meal compared with beverage).

In some analyses, substantial heterogeneity was present. Subgroup analysis explained some, but not all of the heterogeneity. We therefore undertook meta-regression to identify the extent to which both factorial and continuous covariates altered the results.

Meta-regression was carried out in cases in which 10 or more studies were available for each covariate in an analysis. Outcomes with <10 studies were considered to be insufficient to enable a meaningful interpretation of outcomes (44). Where practical, meta-regression was undertaken using OpenMetaAnalyst with a random-effects model (47). Given the small number of studies under investigation, we could not undertake multivariate meta-regression, so each covariate was examined individually.

Heterogeneity of 0–40% as measured by *I*^2^ was defined as potentially unimportant, 30–60% was considered to be potentially moderate heterogeneity, 50–90% was defined as potentially substantial heterogeneity, and 85–100% was defined as potentially considerable heterogeneity (48).

### Study Quality

As all included studies were randomized controlled trials (RCTs), study quality was assessed using the Risk of Bias tool in Review Manager 5.4, based on the Cochrane Handbook for Systematic Reviews (49). The risk of bias was assessed in seven areas: (i) random sequence generation, (ii) allocation concealment, (iii) blinding of participants and personnel, (iv) blinding of outcome assessment, (v) incomplete outcome data (attrition bias), (vi) selective reporting (reporting bias), and (vii) other bias.

### Clinical Relevance

The minimum clinically important differences (MCID) for changes in metabolic measures was taken as follows: HbA1c: 1% (50), fasting blood glucose: 23% (51), fasting blood triglycerides: 30% (52), fasting LDL: 10% (53), fasting HDL: 10% (53), body weight: 5% (54). For standardized mean differences, a change of 0.5 units was taken to be a meaningful change (55). No MCIDs were found for the following outcomes: fasting insulin, HOMA-IR, HOMA2, fasting total cholesterol.

## Results

### Study Search

**Figure 1:**
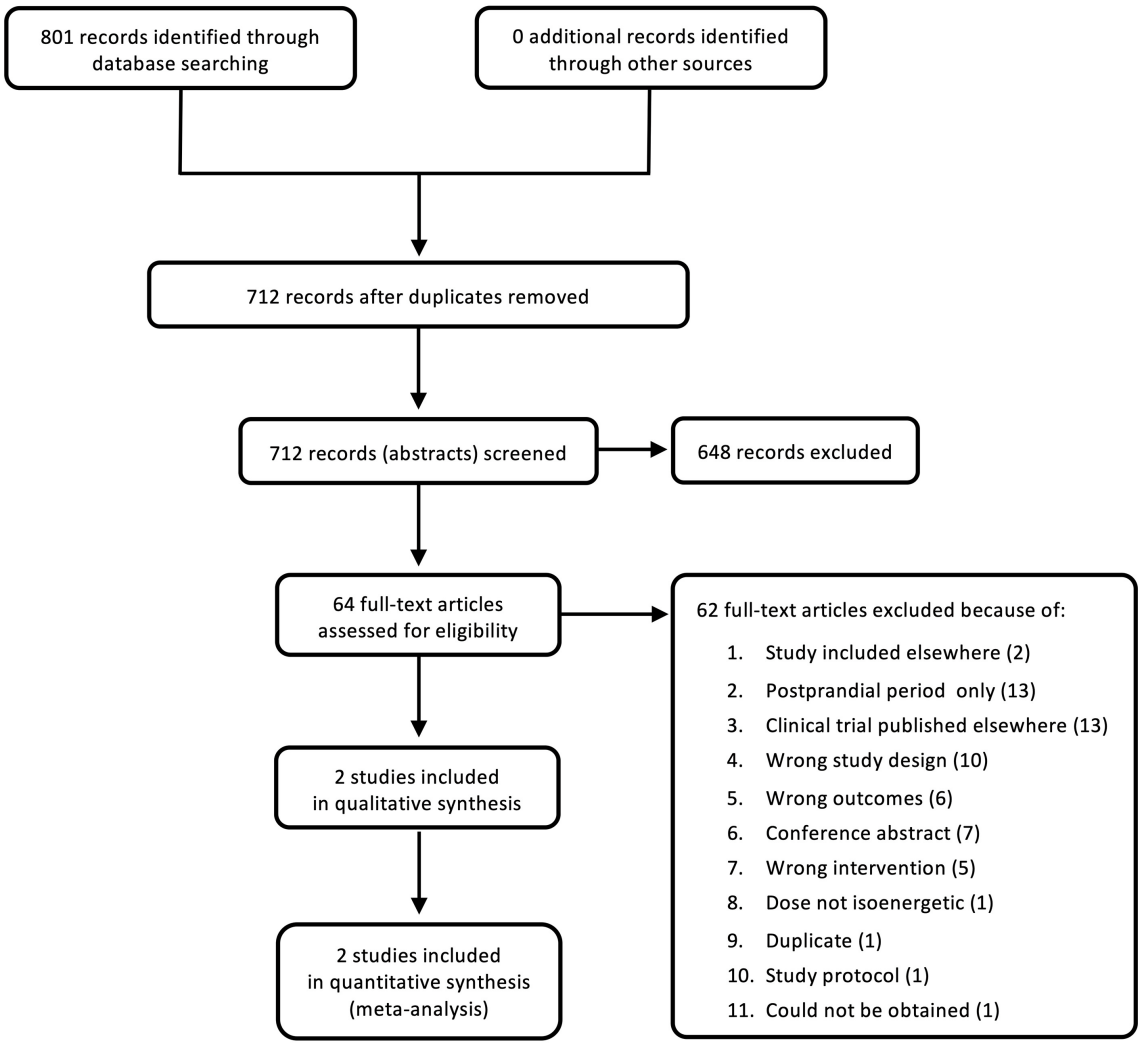
PRISMA flow diagram of searches and study selection.

The search was carried out on September 23, 2020 and yielded 801 references, of which 89 were duplicates. The remaining 712 studies were screened at title and abstract level. From these, 648 studies were deemed to be irrelevant. The remaining 64 full texts were analyzed at full text level. Of these, only two new studies were identified and included into the updated analysis (Figure 1). The majority of full texts were excluded as they dealt with acute, post-prandial effects of fructose, were clinical trials that had not been published, had an inappropriate study design, did not include a measure of glycemic control, were conference abstracts, or had an inappropriate intervention.

### Study Characteristics

The study characteristics of the included studies are shown in Table 1. In addition to the previously identified studies (56, 57, 59–64, 66–68), two new studies were included (58, 65), both of which were carried out in adults without diabetes. Angelopoulos et al. (58) included adults with an average BMI just into the overweight range (26.3 kg/m^2^), whereas Kuzma et al. (65) had two groups of participants, one in the healthy BMI range (average = 23.7) and one in the obese range (average = 31.0). Angelopoulos et al. (58) substituted 9% of each participant's weight-maintaining energy intake with fructose or glucose, whereas Kuzma et al. (65) substituted 25% of each participant's energy needs with fructose or glucose. Both studies were undertaken in adults.

**Table 1:** Characteristics of included studies.

**Study ID (reference)**	**Study kind**	**Study length**	***N* Int**.	***N* Cont**.	**Dose**	**Type of substitution**	**Glucose status**	**Presentation**	**Country**	**Blinding**	**Sex**	**Mean age**	**BMI category**	**Mean BMI**	**Funding**
Aeberli 2011 G40 (56)	X-over	6 × 3 w	29	29	40	Glucose	Norm	Beverage	Switzerland	Yes	M	22.8	Healthy	22.5	Government
Aeberli 2011 G80 (56)	X-over	6 × 3 w	29	29	80	Glucose	Norm	Beverage	Switzerland	Yes	M	22.8	Healthy	22.5	Government
Aeberli 2011 S80 (56)	X-over	6 × 3 w	29	29	80	Sucrose	Norm	Beverage	Switzerland	Yes	M	22.8	Healthy	22.5	Government
Aeberli 2013 G (57)	X-over	4 × 3 w	9	9	80	Glucose	Norm	Beverage	Switzerland	Yes	M	26.3	Healthy	22.4	Government
Aeberli 2013 S (57)	X-over	4 × 3 w	9	9	80	Sucrose	Norm	Beverage	Switzerland	Yes	M	26.3	Healthy	22.4	Government
Angelopolous 2016 (58)	Parallel	10 w	92	94	9% of EEI	Glucose	Norm	Beverage	USA	Yes	Both	37.7	Overweight	26.3	Private
Bantle 2000 (59)	X-over	2 × 6 w	24	24	70	Glucose	Norm	Beverage	USA	Unclear	Both	41.3	Overweight	25.1	Government
Bossetti 1984 (60)	X-over	2 × 2 w	8	8	78.5	Sucrose	Norm	Food	USA	Unclear	Both	26.7	Healthy	22.7	Government
Heden 2014/1 (61)	X-over	2 × 2 w	9	9	35	Glucose	Norm	Beverage	USA	Yes	M	18.3	Healthy	23.5	Government
Heden 2014/2 (61)	X-over	2 × 2 w	11	11	35	Glucose	Norm	Beverage	USA	Yes	M	17.1	Obese	30.6	Government
Heden 2014/3 (61)	X-over	2 × 2 w	11	11	35	Glucose	Norm	Beverage	USA	Yes	F	18.3	Healthy	24.2	Government
Heden 2014/4 (61)	X-over	2 × 2 w	9	9	35	Glucose	Norm	Beverage	USA	Yes	F	17.8	Obese	31.0	Government
Heden 2015 6666 (62)	X-over	2 × 2 w	7	7	35	Glucose	Norm	Beverage	USA	Unclear	Both	18.0	Obese	34.6	Government
Jin 2014 (63)	Parallel	4 w	9	12	99	Glucose	Norm	Beverage	USA	Yes	Both	13.5	Obese	32.6	Government
Koh 1988 I (64)	X-over	2 × 4 w	9	9	15% of EEI	Glucose	Impaired	Food	USA	Unclear	Both	54.0	Overweight	27.3	Government
Koh 1988 N (64)	X-over	2 × 4 w	9	9	15% of EEI	Glucose	Norm	Food	USA	Unclear	Both	50.0	Healthy	23.3	Government
Kuzma 2019 N (65)	X-over	3 × 8 d	12	12	25% of EEI	Glucose	Norm	Beverage	USA	Yes	Both	33.0	Healthy	23.7	Government
Kuzma 2019 O (65)	X-over	3 × 8 d	12	12	25% of EEI	Glucose	Norm	Beverage	USA	Yes	Both	39.0	Obese	31.0	Government
Lowndes 2015 (66)	Parallel	10 w	41	44	9% of EEI	Glucose	Norm	Beverage	USA	Yes	Both	35.7	Overweight	26.0	Private
Malerbi 1996 (67)	X-over	3 × 4 w	16	16	19-20% EEI	Sucrose	Type 2 diabetes	Food	Brazil	Unclear	Both	54.2	Overweight	25.6	Both
Silbernagel 2011 (68)	Parallel	4 w	10	10	150	Glucose	Norm	Beverage	Germany	Yes	Both	30.5	Overweight	25.9	Government

*G40, fructose/glucose 40 g/day; G80, fructose/glucose 80 g/day; G, glucose; S, sucrose; I, impaired glucose tolerance; N, normal glucose tolerance/body weight; O, overweight; X-over, crossover; EEI, estimated energy intake; M, males; F, females; Int., intervention; Cont., control; d, days; w, weeks; m, months*.

### Quality Assessment and Risk of Bias

Data on study quality as determined by the Cochrane 7-item risk of bias analysis is shown in Supplementary Figure 1. The inclusion criteria for study design were restrictive; hence the risk of bias was low for most outcomes. However, as reported in the original analysis (15), not all measures of bias were reliably reported.

### Fasting Blood Glucose

**Figure 2:**
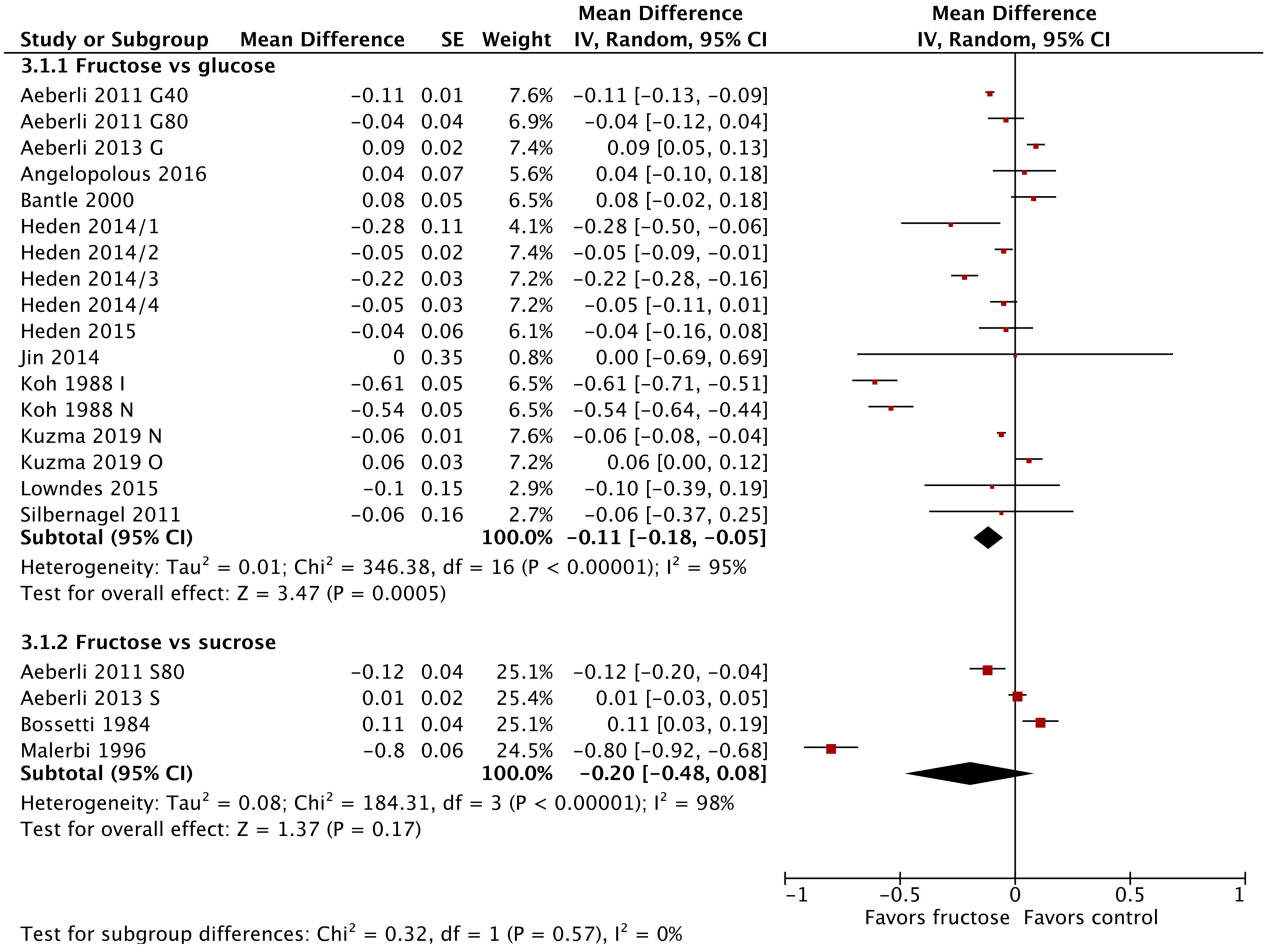
Subgroup meta-analysis of fasting blood glucose following isoenergetic substitution of glucose or sucrose by fructose in food or beverages by substituted sugar. Values are mean differences (95% CIs) (expressed as mmol/L) between fasting blood glucose after fructose consumption and fasting blood glucose following glucose or sucrose consumption. IV, inverse variance; SE, standard error; G40, fructose/glucose 40 g/day; G80, fructose/glucose 80 g/day; G, glucose; S, sucrose; I, impaired glucose tolerance, N, normal glucose tolerance/body weight; O, overweight.

Twenty-one studies/study arms reported on the change in fasting blood glucose following fructose substitution for glucose (17 study arms) or sucrose (4 studies). The addition of the new studies changed the effect size slightly, but not the direction or significance. The substitution of fructose for glucose reduced fasting blood glucose by 0.11 mmol/L (95% CI: −0.18, −0.05; *p* = 0.0005) (Figure 2), but this result was not clinically relevant. There were no significant differences between fructose and sucrose. When grouped by diabetes status, all three groups (normal glucose tolerance, impaired glucose tolerance, type 2 diabetes) showed statistically significant reductions in fasting blood glucose (Supplementary Figure 2). The single studies in people with impaired glucose tolerance (64) and type 2 diabetes (67) showed much larger reductions in fasting blood glucose (−0.61 mmol/L; −0.80 mmol/L, respectively), which were reduced to a statistically significant but not clinically relevant degree. No differences were observed between subgroups when divided by dose or baseline BMI (Supplementary Figures 3, 4).

### HbA1c

Because most studies were done in people without impaired glucose tolerance or diabetes, change in HbA1c was reported by only two studies (Supplementary Figure 5). As each of these studies reported change in HbA1c in a different way, we calculated the standardized mean differences. We found that Koh et al. (64) reported a statistically significant and meaningful difference in HbA1c [SMD = −2.51 (95% CI: −3.44, −1.57), *p* < 0.00001], whereas the change HbA1c reported by Malerbi et al. (67) was not significant (58).

### HOMA

The 13 studies/study arms that reported on HOMA used both HOMA-IR and HOMA2 as outcomes. In order to combine the HOMA results of all studies, we used a standardized mean difference analysis (Supplementary Figures 6–9). There were no significant differences between fructose and glucose [SMD = 0.11 (95% CI: −0.34, 0.56); *p* = 0.64]. A single study (56) that compared fructose with sucrose found a statistically significant increase in HOMA2 after fructose consumption.

### Fasting Blood Insulin

**Figure 3:**
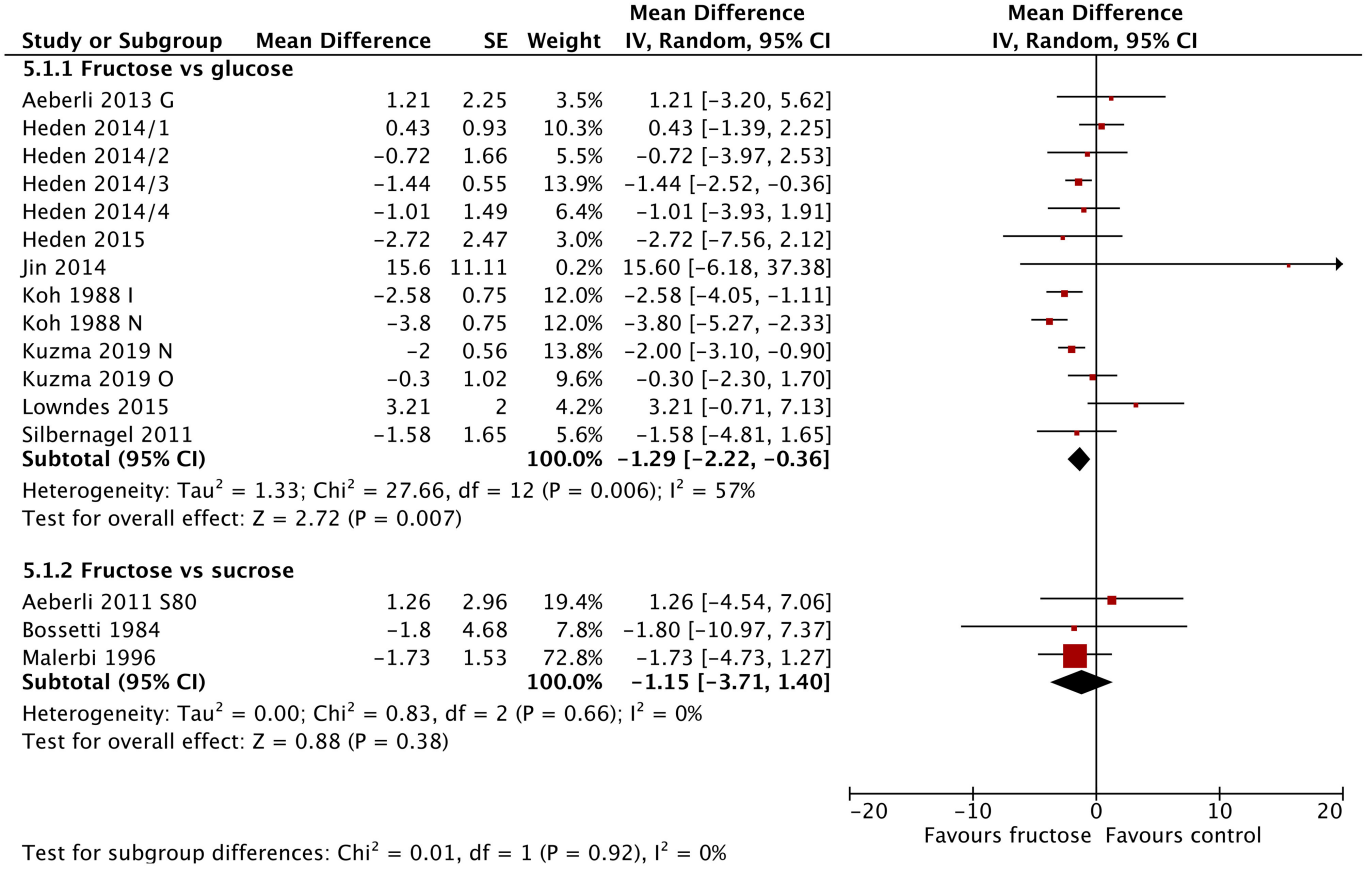
Subgroup meta-analysis of fasting blood insulin following isoenergetic substitution of glucose or sucrose by fructose in food or beverages by substituted sugar. Values are mean differences (95% CIs) (expressed as μIU/mL) between fasting blood glucose after fructose consumption and fasting blood glucose following glucose or sucrose consumption. IV, inverse variance; SE, standard error; G, glucose; S, sucrose; I, impaired glucose tolerance, N, normal glucose tolerance/body weight; O, overweight.

Sixteen studies/study arms reported on changes in fasting blood insulin following fructose substitution for glucose (13 study arms) or sucrose (three studies). Fasting blood insulin reduced significantly following fructose consumption compared with glucose consumption [MD = −1.29 μIU/mL (95% CI: −2.22, −0.36), *p* = 0.007] (Figure 3). The comparison with sucrose revealed similar results but was not statistically significant. Fasting insulin was also statistically significantly lowered in studies using lower doses (30–40 g/day) [MD = −1.00 μIU/mL (95% CI: −1.84, −0.16), *p* = 0.02] and in studies using doses >80 g/day [MD = −1.49 μIU/mL (95% CI: −2.55, −0.44), *p* = 0.005]. Studies in people without diabetes at baseline also showed statistically significant reductions in fasting blood insulin [MD = −0.82 μIU/mL (95% CI: −1.52, −0.12), *p* = 0.02] (Supplementary Figures 10–12). Baseline BMI did not influence blood insulin concentrations.

### Fasting Blood Lipids

#### Total Cholesterol

**Figure 4:**
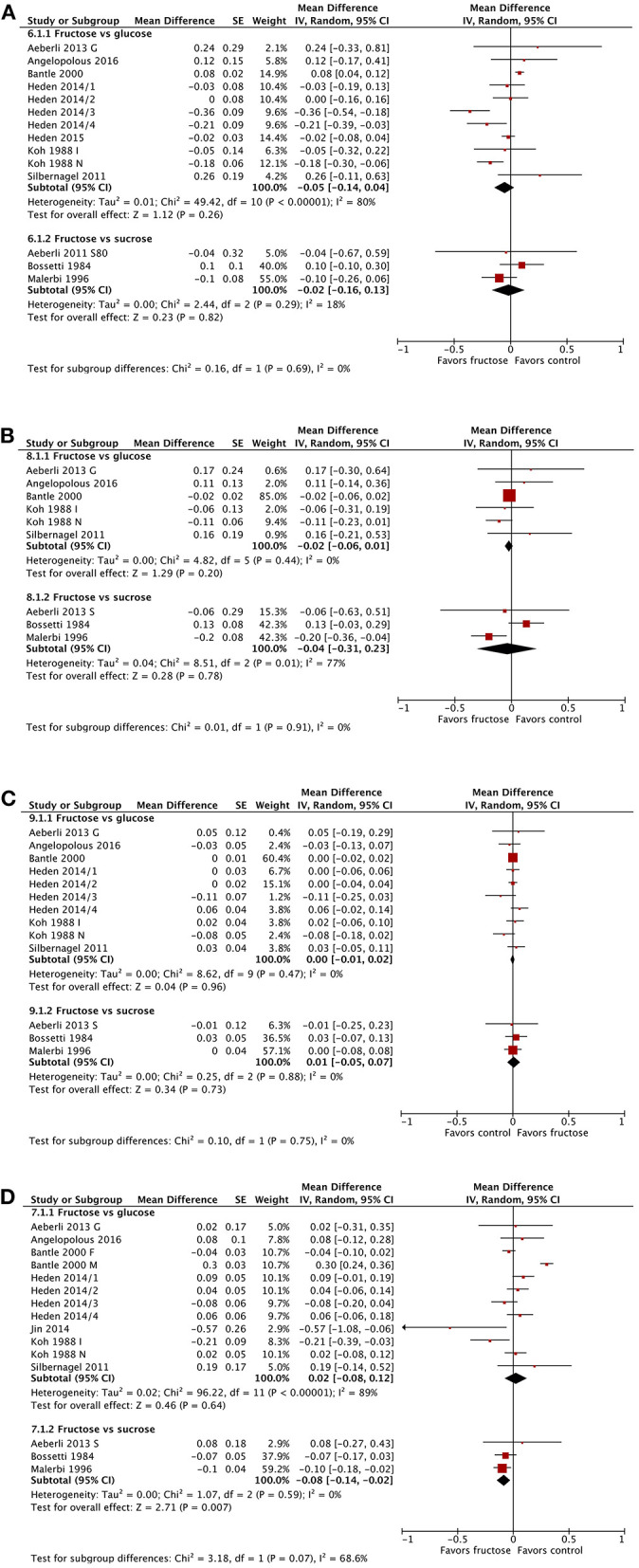
Subgroup meta-analysis of fasting total cholesterol **(A)**, low density lipoprotein **(B)**, high density lipoprotein **(C)**, and triglycerides **(D)** following isoenergetic substitution of glucose or sucrose by fructose in food or beverages by substituted sugar. Values are mean differences (95% CIs) (expressed as mmol/L) between fasting blood glucose after fructose consumption and fasting blood glucose following glucose or sucrose consumption. IV, inverse variance; SE, standard error; G, glucose; S, sucrose; I, impaired glucose tolerance, N, normal glucose tolerance/body weight; O, overweight.

The substitution of fructose for glucose or sucrose did not result in any significant changes in total cholesterol (Figure 4A). This did not differ when subgrouped by baseline BMI, dose, or diabetes status (Supplementary Figures 13–15).

#### Low Density Lipoproteins

The substitution of fructose for glucose or sucrose did not result in any significant changes in LDL cholesterol (Figure 4B), except when subgrouped by diabetes status (Supplementary Figure 16). The single study in people with type 2 diabetes showed a statistically but not clinically significant reduction in LDL following fructose consumption. This subgroup was also statistically different from the subgroup of studies in people without diabetes. No differences were apparent when subgrouping by BMI or dose (Supplementary Figures 17, 18).

#### High Density Lipoproteins

No changes in HDL were apparent (Figure 4C). No statistically significant differences emerged between any subgroups, by BMI, dose, or diabetes status (Supplementary Figures 19–21).

#### Triglycerides

The substitution of fructose for glucose or sucrose showed no significant changes in fasting triglyceride concentrations, except in the three studies comparing fructose with sucrose consumption (Figure 4D); this change was not clinically relevant. Subgrouping by baseline BMI or dose did not reveal any significant differences (Supplementary Figures 22, 23). When subgrouped by diabetes status, people with impaired glucose tolerance and those with type 2 diabetes showed statistically but not clinically relevant reductions in fasting triglycerides; however, each group was represented by only a single study in each group (Supplementary Figure 24).

### Body Weight

**Figure 5:**
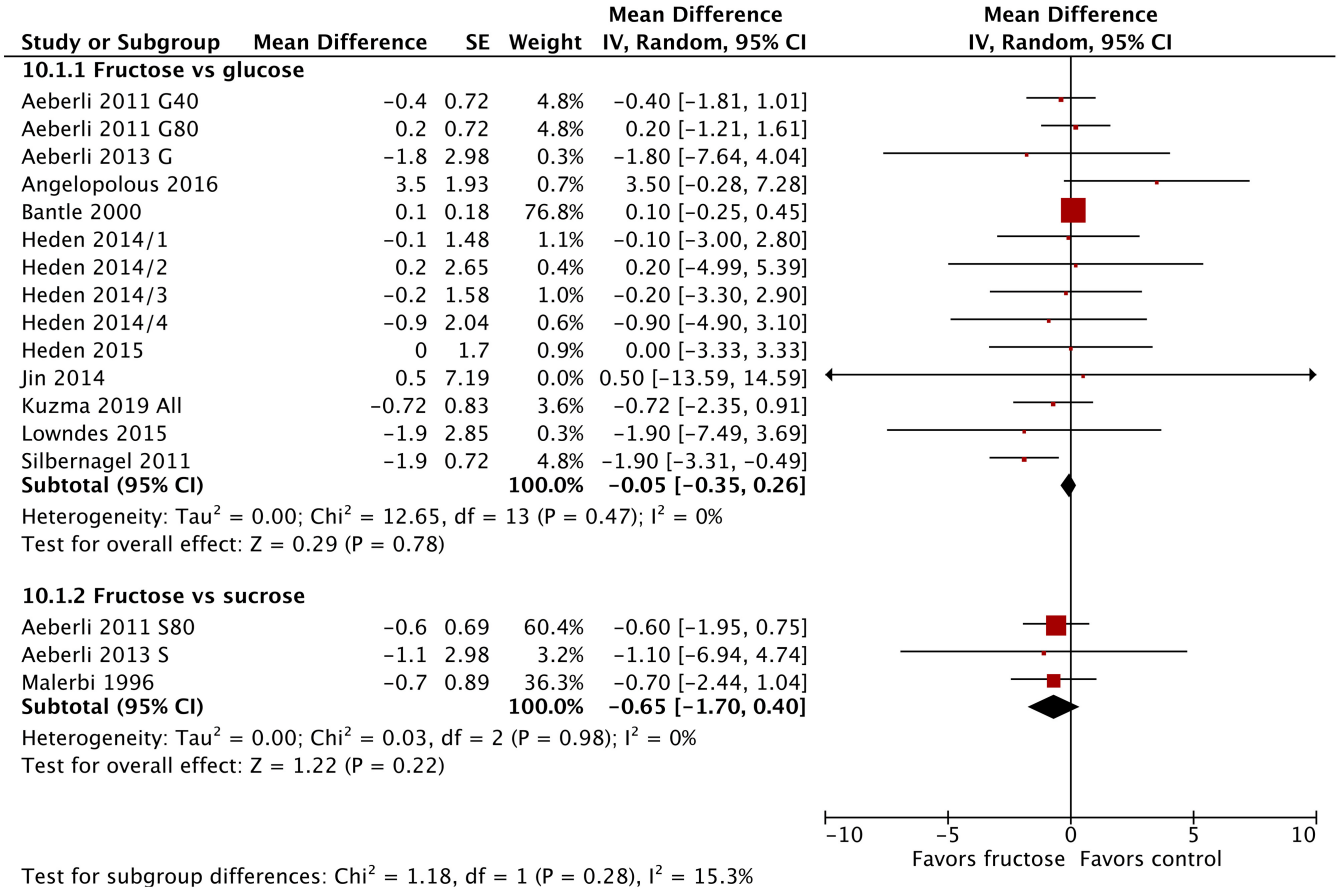
Subgroup meta-analysis of body weight following isoenergetic substitution of glucose or sucrose by fructose in food or beverages by substituted sugar. Values are mean differences (95% CIs) (expressed as kg) between fasting blood glucose after fructose consumption and fasting blood glucose following glucose or sucrose consumption. IV, inverse variance; SE, standard error; G40, fructose/glucose 40 g/day; G80, fructose/glucose 80 g/day; G, glucose; S, sucrose; I, impaired glucose tolerance, N, normal glucose tolerance/body weight; O, overweight.

Body weight was not significantly influenced by the substitution of fructose for glucose or sucrose (Figure 5). Similarly, subgroup analysis found no differences in body weight by baseline BMI or diabetes status (Supplementary Figures 25, 26), with the exception of dose. Studies using very high doses of fructose (>80 g/day) resulted in a statistically significant reduction in body weight [MD = −1.20 kg (95% CI: −2.11, −0.29), *p* = 0.01] (Supplementary Figure 27). This difference was not clinically significant.

### Meta-Regression

The results of our meta-regression analyses are presented in Table 2 and Supplementary Tables 1–3. For fasting blood glucose, a number of significant results came from single studies (e.g., impaired glucose tolerance, type 2 diabetes, funding from both industry, and government); these were ignored. However, a statistically significant difference was observed between the studies that provided food as the source of sugar rather than beverages, and for studies that blinded the participants to their allocation compared with those that provided food or kept account of the participants' diets. Similarly, both age of participants and year of publication were significantly associated with changes in fasting blood glucose. Unfortunately, the four food-based study arms were also among the studies causing a great deal of heterogeneity in the meta-regression by age of study (60, 64, 67), thus it is not clear if the difference came from the use of food, or simply from the age of the study.

**Table 2:** Meta-regression of mean differences in fasting blood glucose concentrations between consumption of fructose compared with sucrose or glucose by factor and continuous covariates.

**Potential covariant**	***N* study arms**	**Model coefficient**	**Lower bound**	**Upper bound**	***p*-value**	
**Factor covariates**						
Control sugar	Glucose	17				
	Sucrose	4	−0.079	−0.338	0.180	0.552
Diabetes status	Normoglycaemia	19				
	Impaired glucose tolerance	1	−0.545	−0.833	−0.256	**<0.001**
	Type 2 diabetes	1	−0.735	−1.031	−0.439	**<0.001**
Study type	Cross-over	17				
	Parallel	4	0.119	−0.184	0.421	0.442
Food vs. beverage	Beverage	17				
	Food	4	−0.405	−0.600	−0.220	**<0.001**
Gender	Males	7				
	Both	12	−0.107	−0.328	0.114	0.343
	Females	2	−0.069	−0.431	0.293	0.703
Funding	Government	18				
	Both	1	−0.697	−1.076	−0.319	**<0.001**
	Industry	2	0.086	−0.216	0.389	0.575
Blinding	Yes	15				
	Unclear	6	−0.239	−0.444	−0.034	**0.022**
**Continuous covariates**						
BMI	Range: 22.4–34.6	21	0.004	−0.025	0.034	0.766
Age	Range: 13.5–54.2	21	−0.012	−0.019	−0.005	**<0.001**
Dose	Range: 35–150	21	0.001	−0.003	0.004	0.633
Year of publication	Range: 1984–2019	21	0.011	0.003	0.020	**0.009**

*All data, including model coefficients, confidence intervals, and p-values were obtained using random-effect models and 95% confidence intervals in OpenMetaAnalyst. Values in bold represent statistically significant results*.

Meta-regression of the studies for fasting insulin similarly revealed differences arising from covariates (Supplementary Table 1). Unclear blinding, age of participants and year of publication all influenced the outcomes. Interestingly, studies in males were more likely to be associated with an increase in fasting blood insulin, compared with those in females.

Meta-regression of body weight by the same covariates revealed only dose as a significant influence on the outcome. The coefficient was very small, however, and this result is likely to be of limited relevance.

Meta-regression of fasting triglyceride concentrations showed several significant covariates. As seen in our previous analyses, the use of food instead of beverages as the vehicle for fructose delivery significantly reduced fasting triglycerides following fructose consumption compared with sucrose or glucose consumption. Interestingly, males were again more likely to have increased fasting triglyceride concentrations following fructose consumption compared with mixed or female-only studies, in whom significant reductions in fasting triglycerides were observed.

## Discussion

The results of our updated meta-analysis repeat and strengthen our previous findings on the lack of harmful effects specific to fructose consumption (15) in line with similar analyses (18–20, 22, 23), at least in the outcomes reported by these reviews. A reiteration and new discussion of these findings is warranted because many narrative reviews still propagate the view that fructose is more harmful than other sugars (31–36, 69, 70).

Our findings did show statistically significant reductions in fasting blood glucose (FBG) concentrations, fasting blood insulin (FBI) concentrations, and body weight. However, none of these differences was clinically relevant. Meta-regression did reveal some interesting findings, which require further investigation. For example, we found that presenting the sugar in foods rather than in beverages considerably altered the effect size for changes in FBG and FBI. However, we also found that the age of the study had a similar effect. The answer as to which of these covariates is causing this change is obscured by the fact that the food studies were also older.

Another interesting correlation to emerge from our meta-regression was the finding that the sex of the participants was associated with quite different outcomes. For example, overall fructose consumption lowered FBI and had no significant effect on triglycerides. Meta-regression by sex, however, found that female sex was associated with a reduction in FBI, whereas male sex was associated with no reduction in FBI. Similarly, female sex was associated with a statistically significant reduction in fasting triglycerides, whereas male sex was associated with a statistically significant increase in fasting triglycerides. Differences by sex in glucose homeostasis and lipoproteins have been described previously (71, 72). It is therefore possible that changes in these outcomes are also influenced by sex.

Even after subgroup meta-analysis and meta-regression, much heterogeneity remained. The lack of studies that covered multiple potential covariates, along with a small number of studies in total, did not allow us to investigate the sources of heterogeneity. We are also aware that undertaking multiple analyses will increase the possibility of statistical significance arising by chance. One should therefore be careful not to overinterpret some of our findings.

Of note, we are not stating that consumption of sugar, especially as refined carbohydrates, is advisable or beneficial. Consumption of highly energy-dense foods without significant fiber and/or micronutrient content is certainly inadvisable (73). For this reason, the WHO recommends that <10% of daily energy should come from free sugars (74). We argue purely that ascribing harmful effects to fructose in particular is counter to the evidence. Where the use of sugar will continue (e.g., as a preservative or in home-made cakes and other treats), information on the post-prandial benefits of fructose (e.g., a reduction in peak post-prandial blood glucose and insulin concentrations), particularly in those with impaired glucose tolerance, type 1 and type 2 diabetes, should be provided.

Our study design deliberately selected for studies that kept the diets between the groups isocaloric. However, there are other factors at play that influence real world weight gain. For example, it has been shown that fructose increases the subjective sensation of hunger and food-seeking behavior in functional MRI studies (45, 75) although recent work comparing the actual food intakes following glucose-, fructose-, high fructose corn syrup-, and aspartame-sweetened beverages found no difference between any sugar in the total number of calories consumed over 8 days (76). Furthermore, Silbernagel et al. found a statistically significant reduction in body weight following 4 weeks of fructose consumption (68). These partially contradictory findings suggest that further research should be conducted before any conclusions are made.

Interestingly, it appears that a significant proportion of ingested fructose is converted to glucose in the small intestine (77, 78), with only very large doses spilling over to the liver (79). This was first shown over 50 years ago in an elegant study by Öckerman and Lundborg (80). In this study, the authors administered fructose or galactose to humans directly into the jejunum at doses ranging from 37.5 to 150 g. Up to 70% of the fructose could be recovered as glucose in the mesenteric veins, while an administration of galactose did not result in the recovery of glucose. More recently, it has been established that the small intestine expresses fructokinase along with other fructolytic and gluconeogenic enzymes (77) and that their expression is regulated by GLUT5 (a glucose transporter protein) and KHK (ketohexokinase) (81). Thus, it is unlikely that small or moderate amounts of fructose are necessarily be more harmful than equivalent amounts of glucose, because at usual levels of consumption, most of the fructose simply never reaches the liver.

In the 4 years since our earlier search for articles on the effects of chronic fructose consumption, only two studies were published on isoenergetic fructose replacement for sucrose or glucose for periods longer than the immediate post-prandial period. However, 17 new fructose studies were published that were concerned with the post-prandial time period, despite little chance of these studies significantly changing the effect sizes already generated by previous meta-analyses.

The two new chronic studies (58, 65) were carried out in people with normal fasting blood glucose concentrations. In one study (65) only 24 participants were enrolled, half of whom had a healthy weight at baseline. The other study (58) enrolled more (i.e., 186) participants, but unfortunately, the average BMI at baseline was only just into the overweight range, and people with diabetes were actively excluded. Given the paucity of evidence in people with impaired glucose tolerance or diabetes, we find this disappointing. We therefore renew our call for high quality, isoenergetic studies to be carried out in people with a lack of glycemic control; else evidence-based dietary advice for these populations will continue to be lacking.

This updated systematic review and meta-analysis has confirmed our previous findings, i.e., that even high doses of fructose consumed daily do not adversely affect health when compared with isoenergetic amounts of sucrose or glucose. The absence of high-quality studies in people with, or at risk of diabetes hampers our ability to make specific recommendations based on diabetes status. Similarly, the large number of covariates and small number of studies did not allow us to investigate residual confounding through multivariate meta-regression. The little evidence we have in populations with diabetes does not support the claim that fructose is harmful for people with this condition; indeed, the opposite seems true. Whether these beneficial effects are real, however, can only be established with more evidence.

## Data Availability Statement

The raw data supporting the conclusions of this article will be made available by the authors, without undue reservation.

## Author Contributions

KM designed the study, carried out the search, and drafted the manuscript. KM and MZ did title/abstract and full text inclusion, data extraction/checking, and data analysis. KM and MF designed the analyses. MF and MZ critically analyzed the manuscript and suggested edits. All authors contributed to the article and approved the submitted version.

## Conflict of Interest

The authors declare that the research was conducted in the absence of any commercial or financial relationships that could be construed as a potential conflict of interest.
