# Supplementary material for: Chronic Fructose Substitution for Glucose or Sucrose in Food or Beverages and Metabolic Outcomes: An Updated Systematic Review and Meta-Analysis
Source: Front Nutr. 2021 Apr 28;8:647600. doi: 10.3389/fnut.2021.647600 (PMC8113762; doi:10.3389/fnut.2021.647600)
Supplement: Supplementary file 1 [file Data_Sheet_1.pdf]

Supplementary Material

Table of Contents

Supplementary figure 1: Risk of bias in included studies. 3

Supplementary figure 2: Subgroup meta-analysis of fasting blood glucose following isoenergetic substitution of glucose or sucrose by fructose in food or beverages by diabetes status. 4

Supplementary figure 3: Subgroup meta-analysis of fasting blood glucose following isoenergetic substitution of glucose or sucrose by fructose in food or beverages by dose of sugar. 5

Supplementary figure 4: Subgroup meta-analysis of fasting blood glucose following isoenergetic substitution of glucose or sucrose by fructose in food or beverages by baseline BMI. 6

Supplementary figure 5: Subgroup meta-analysis of HbA1c following isoenergetic substitution of glucose or sucrose by fructose in food or beverages by substituted sugar. 7

Supplementary figure 6: Subgroup meta-analysis of HOMA-IR/HOMA2 following isoenergetic substitution of glucose or sucrose by fructose in food or beverages by substituted sugar. 8

Supplementary figure 7: Subgroup meta-analysis of HOMA-IR/HOMA2 following isoenergetic substitution of glucose or sucrose by fructose in food or beverages by baseline BMI. 9

Supplementary figure 8: Subgroup meta-analysis of HOMA-IR/HOMA2 following isoenergetic substitution of glucose or sucrose by fructose in food or beverages by dose of sugar. 10

Supplementary figure 9: Subgroup meta-analysis of HOMA-IR/HOMA2 following isoenergetic substitution of glucose or sucrose by fructose in food or beverages by diabetes status. 11

Supplementary figure 10: Subgroup meta-analysis of fasting blood insulin following isoenergetic substitution of glucose or sucrose by fructose in food or beverages by dose of sugar. 12

Supplementary figure 11: Subgroup meta-analysis of fasting blood insulin following isoenergetic substitution of glucose or sucrose by fructose in food or beverages by dose of sugar. 13

Supplementary figure 12: Subgroup meta-analysis of fasting blood insulin following isoenergetic substitution of glucose or sucrose by fructose in food or beverages by baseline BMI. 14

Supplementary figure 13: Subgroup meta-analysis of fasting blood total cholesterol following isoenergetic substitution of glucose or sucrose by fructose in food or beverages by baseline BMI. ... 15

Supplementary figure 14: Subgroup meta-analysis of fasting blood total cholesterol following isoenergetic substitution of glucose or sucrose by fructose in food or beverages by dose of sugar. ... 16

Supplementary figure 15: Subgroup meta-analysis of fasting blood total cholesterol following isoenergetic substitution of glucose or sucrose by fructose in food or beverages by diabetes status. 17

Supplementary figure 16: Subgroup meta-analysis of fasting LDL cholesterol following isoenergetic substitution of glucose or sucrose by fructose in food or beverages by diabetes status. 18

Supplementary figure 17: Subgroup meta-analysis of fasting LDL cholesterol following isoenergetic substitution of glucose or sucrose by fructose in food or beverages by baseline BMI. 19

Supplementary figure 18: Subgroup meta-analysis of fasting LDL cholesterol following isoenergetic substitution of glucose or sucrose by fructose in food or beverages by dose of sugar. 20

Supplementary figure 19: Subgroup meta-analysis of fasting HDL cholesterol following isoenergetic substitution of glucose or sucrose by fructose in food or beverages by baseline BMI. 21

Supplementary figure 20: Subgroup meta-analysis of fasting HDL cholesterol following isoenergetic substitution of glucose or sucrose by fructose in food or beverages by dose of sugar. 22

Supplementary figure 21: Subgroup meta-analysis of fasting HDL cholesterol following isoenergetic substitution of glucose or sucrose by fructose in food or beverages by diabetes status. 23

Supplementary figure 22: Subgroup meta-analysis of fasting blood triglycerides following isoenergetic substitution of glucose or sucrose by fructose in food or beverages by baseline BMI. ... 24

Supplementary figure 23: Subgroup meta-analysis of fasting blood triglycerides following isoenergetic substitution of glucose or sucrose by fructose in food or beverages by dose of sugar. ... 25

Supplementary figure 24: Subgroup meta-analysis of fasting blood triglycerides following isoenergetic substitution of glucose or sucrose by fructose in food or beverages by diabetes status. 26

Supplementary figure 26: Subgroup meta-analysis of body weight following isoenergetic substitution of glucose or sucrose by fructose in food or beverages by diabetes status. 28

Supplementary figure 27: Subgroup meta-analysis of body weight following isoenergetic substitution of glucose or sucrose by fructose in food or beverages by dose of sugar. 29

Supplementary table 1: meta-regression of studies reporting on fasting blood insulin 30

Supplementary table 2: meta-regression of studies reporting on body weight 31

Supplementary table 2: meta-regression of studies reporting on fasting blood triglycerides 32

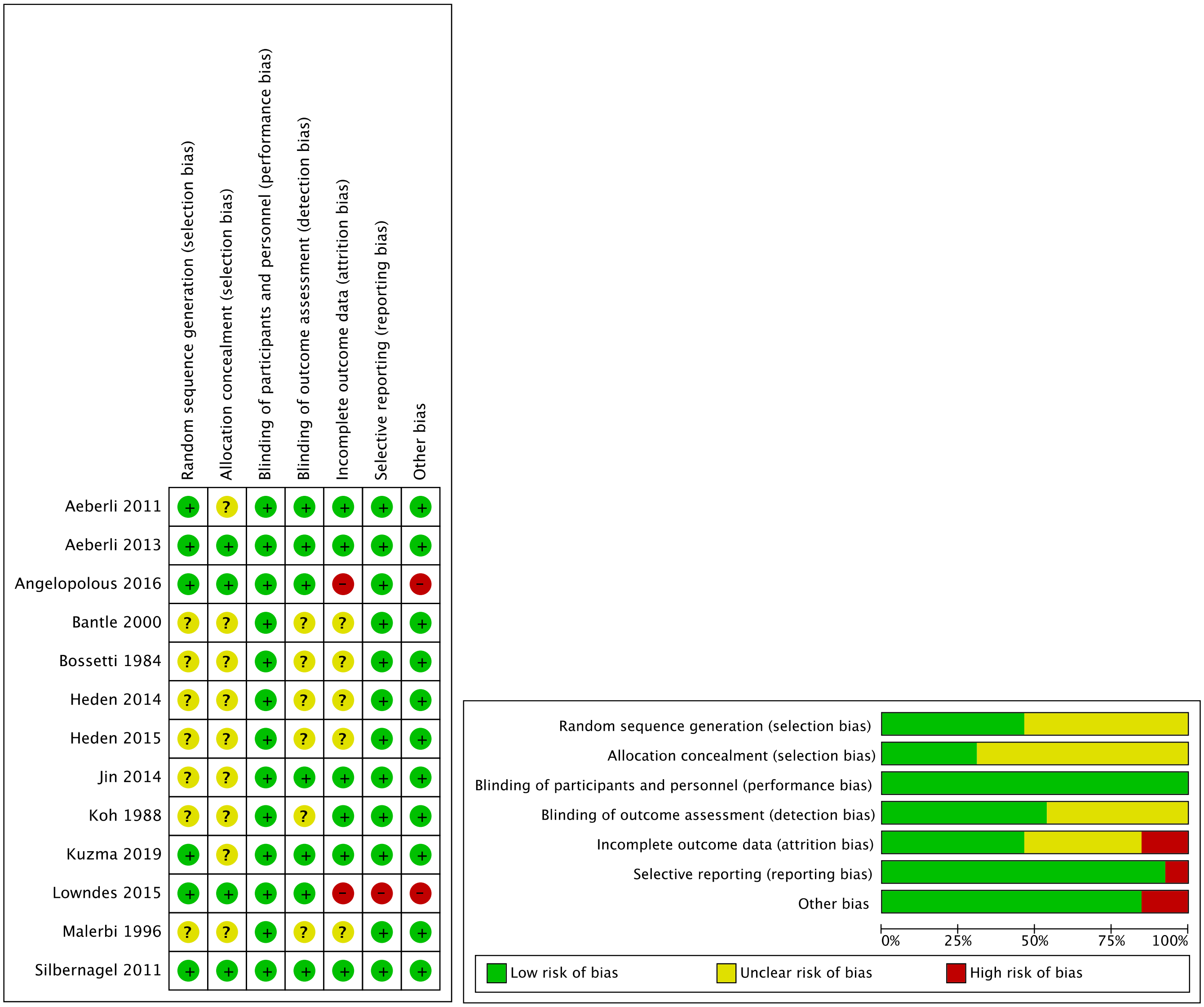

Supplementary figure 1: Risk of bias in included studies.

Study or Subgroup	Mean Difference	SE	Weight	Mean Difference IV, Random, 95% CI
**3.4.1 No diabetes**				
Aeberli 2011 G40	−0.11	0.01	7.0%	−0.11 [−0.13, −0.09]
Aeberli 2011 G80	−0.04	0.04	6.0%	−0.04 [−0.12, 0.04]
Aeberli 2011 S80	−0.12	0.04	6.0%	−0.12 [−0.20, −0.04]
Aeberli 2013 G	0.09	0.02	6.8%	0.09 [0.05, 0.13]
Aeberli 2013 S	0.01	0.02	6.8%	0.01 [−0.03, 0.05]
Angelopolous 2016	0.04	0.07	4.6%	0.04 [−0.10, 0.18]
Bantle 2000	0.08	0.05	5.6%	0.08 [−0.02, 0.18]
Bossetti 1984	0.11	0.04	6.0%	0.11 [0.03, 0.19]
Heden 2014/1	−0.28	0.11	3.0%	−0.28 [−0.50, −0.06]
Heden 2014/2	−0.05	0.02	6.8%	−0.05 [−0.09, −0.01]
Heden 2014/3	−0.22	0.03	6.5%	−0.22 [−0.28, −0.16]
Heden 2014/4	−0.05	0.03	6.5%	−0.05 [−0.11, 0.01]
Heden 2015	−0.04	0.06	5.1%	−0.04 [−0.16, 0.08]
Jin 2014	0	0.35	0.5%	0.00 [−0.69, 0.69]
Koh 1988 N	−0.54	0.05	5.6%	−0.54 [−0.64, −0.44]
Kuzma 2019 N	−0.06	0.01	7.0%	−0.06 [−0.08, −0.04]
Kuzma 2019 O	0.06	0.03	6.5%	0.06 [0.00, 0.12]
Lowndes 2015	−0.1	0.15	2.0%	−0.10 [−0.39, 0.19]
Silbernagel 2011	−0.06	0.16	1.8%	−0.06 [−0.37, 0.25]
**Subtotal (95% CI)**			**100.0%**	**−0.06 [−0.11, −0.01]**
Heterogeneity: $Tau^2 = 0.01$; $Chi^2 = 265.12$, df = 18 ($P < 0.00001$); $I^2 = 93\%$				
Test for overall effect: Z = 2.48 (P = 0.01)				
**3.4.2 Impaired glucose tolerance**				
Koh 1988 I	−0.61	0.05	100.0%	−0.61 [−0.71, −0.51]
**Subtotal (95% CI)**			**100.0%**	**−0.61 [−0.71, −0.51]**
Heterogeneity: Not applicable				
Test for overall effect: Z = 12.20 ($P < 0.00001$)				
**3.4.4 Type 2 diabetes**				
Malerbi 1996	−0.8	0.06	100.0%	−0.80 [−0.92, −0.68]
**Subtotal (95% CI)**			**100.0%**	**−0.80 [−0.92, −0.68]**
Heterogeneity: Not applicable				
Test for overall effect: Z = 13.33 ($P < 0.00001$)				

Test for subgroup differences: $Chi^2 = 191.22$, df = 2 ($P < 0.00001$), $I^2 = 99.0\%$

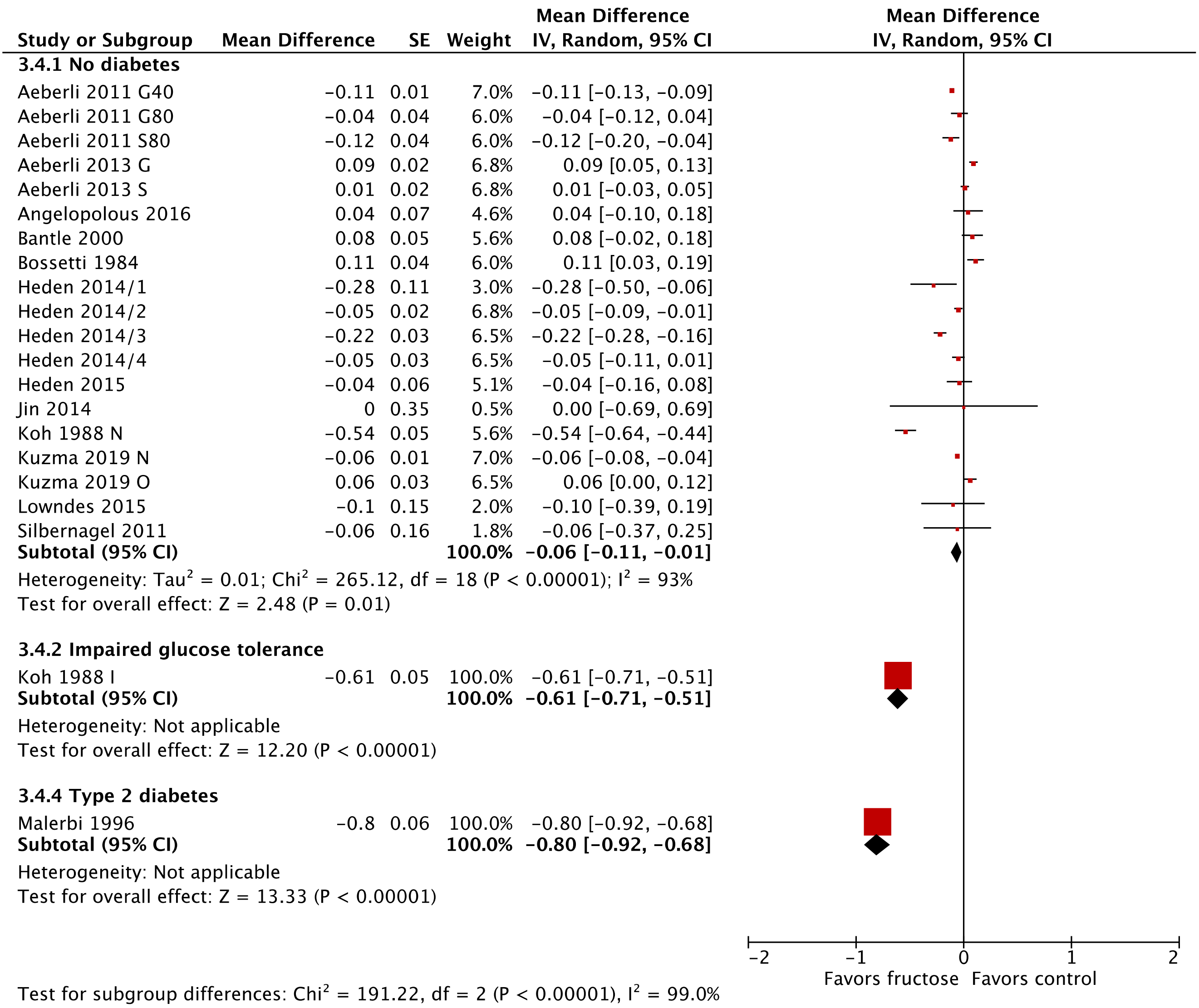

Supplementary figure 2: Subgroup meta-analysis of fasting blood glucose following isoenergetic substitution of glucose or sucrose by fructose in food or beverages by diabetes status. Values are mean differences [95% CIs] (expressed as mmol/L) between fasting blood glucose after fructose consumption and fasting blood glucose following glucose or sucrose consumption. IV, inverse variance; SE, standard error; G40, fructose/glucose 40 g/day; G80, fructose/glucose 80 g/day; G, glucose; S, sucrose; I, impaired glucose tolerance, N, normal glucose tolerance/body weight; O, overweight

Study or Subgroup	Mean Difference	SE	Weight	Mean Difference IV, Random, 95% CI
**3.3.1 30–40 grams/day**				
Aeberli 2011 G40	−0.11	0.01	23.8%	−0.11 [−0.13, −0.09]
Heden 2014/1	−0.28	0.11	5.0%	−0.28 [−0.50, −0.06]
Heden 2014/2	−0.05	0.02	21.7%	−0.05 [−0.09, −0.01]
Heden 2014/3	−0.22	0.03	19.0%	−0.22 [−0.28, −0.16]
Heden 2014/4	−0.05	0.03	19.0%	−0.05 [−0.11, 0.01]
Heden 2015	−0.04	0.06	11.4%	−0.04 [−0.16, 0.08]
**Subtotal (95% CI)**			**100.0%**	**−0.11 [−0.16, −0.05]**
Heterogeneity: $Tau^2 = 0.00$; $Chi^2 = 29.53$, df = 5 ($P < 0.0001$); $I^2 = 83\%$				
Test for overall effect: Z = 3.87 (P = 0.0001)				
**3.3.2 41–80 g/day**				
Aeberli 2011 G80	−0.04	0.04	10.4%	−0.04 [−0.12, 0.04]
Aeberli 2011 S80	−0.12	0.04	10.4%	−0.12 [−0.20, −0.04]
Aeberli 2013 G	0.09	0.02	10.7%	0.09 [0.05, 0.13]
Aeberli 2013 S	0.01	0.02	10.7%	0.01 [−0.03, 0.05]
Angelopolous 2016	0.04	0.07	9.7%	0.04 [−0.10, 0.18]
Bantle 2000	0.08	0.05	10.2%	0.08 [−0.02, 0.18]
Bossetti 1984	0.11	0.04	10.4%	0.11 [0.03, 0.19]
Koh 1988 I	−0.61	0.05	10.2%	−0.61 [−0.71, −0.51]
Koh 1988 N	−0.54	0.05	10.2%	−0.54 [−0.64, −0.44]
Lowndes 2015	−0.1	0.15	7.1%	−0.10 [−0.39, 0.19]
**Subtotal (95% CI)**			**100.0%**	**−0.11 [−0.24, 0.03]**
Heterogeneity: $Tau^2 = 0.04$; $Chi^2 = 301.80$, df = 9 ($P < 0.00001$); $I^2 = 97\%$				
Test for overall effect: Z = 1.55 (P = 0.12)				
**3.3.3 >80 g/day**				
Jin 2014	0	0.35	8.6%	0.00 [−0.69, 0.69]
Kuzma 2019 N	−0.06	0.01	25.1%	−0.06 [−0.08, −0.04]
Kuzma 2019 O	0.06	0.03	24.7%	0.06 [0.00, 0.12]
Malerbi 1996	−0.8	0.06	23.7%	−0.80 [−0.92, −0.68]
Silbernagel 2011	−0.06	0.16	17.9%	−0.06 [−0.37, 0.25]
**Subtotal (95% CI)**			**100.0%**	**−0.20 [−0.45, 0.05]**
Heterogeneity: $Tau^2 = 0.06$; $Chi^2 = 167.69$, df = 4 ($P < 0.00001$); $I^2 = 98\%$				
Test for overall effect: Z = 1.59 (P = 0.11)				

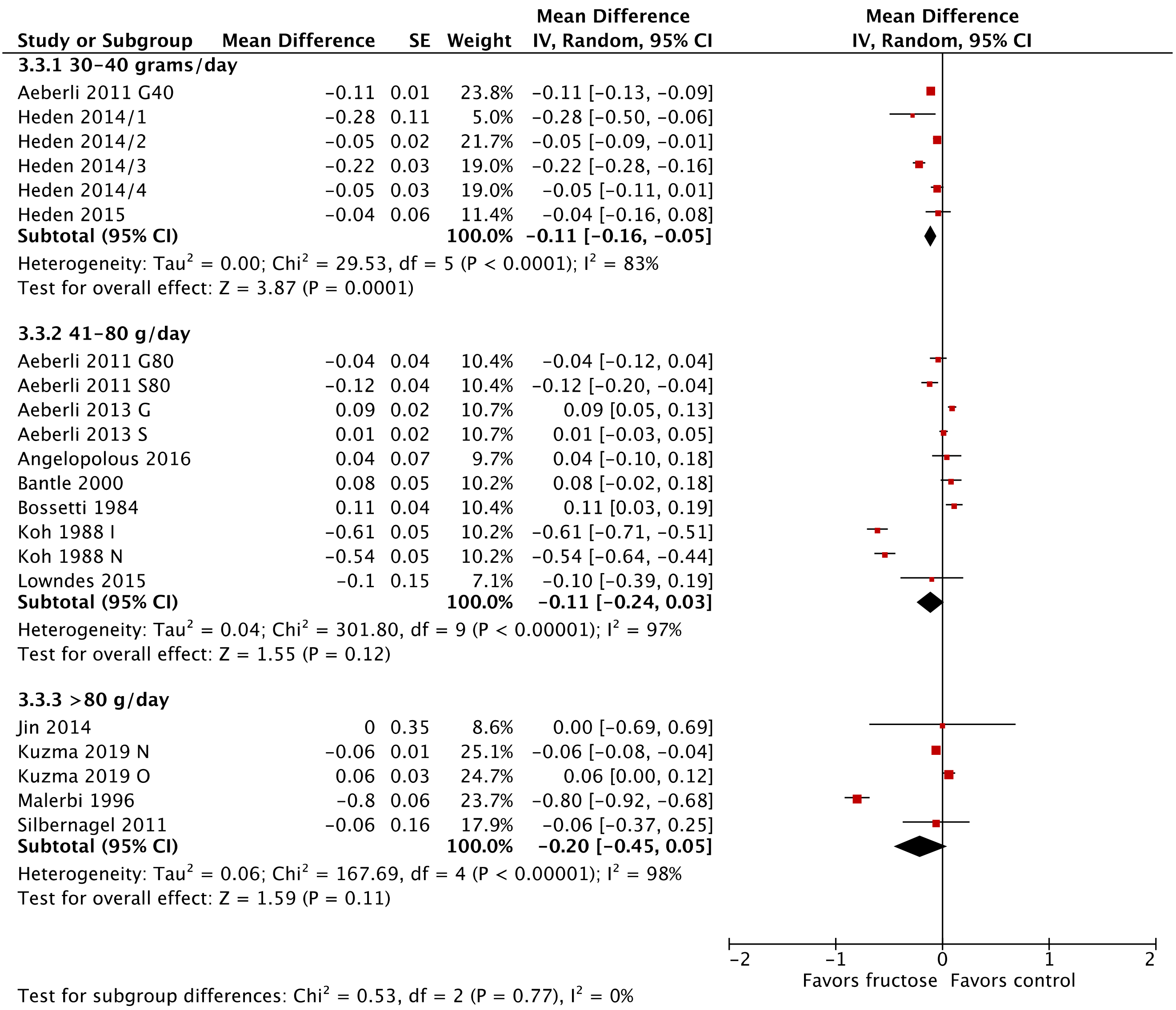

Test for subgroup differences: $Chi^2 = 0.53$, df = 2 ($P = 0.77$), $I^2 = 0\%$

Supplementary figure 3: Subgroup meta-analysis of fasting blood glucose following isoenergetic substitution of glucose or sucrose by fructose in food or beverages by dose of sugar. Values are mean differences [95% CIs] (expressed as mmol/L) between fasting blood glucose after fructose consumption and fasting blood glucose following glucose or sucrose consumption. IV, inverse variance; SE, standard error; G40, fructose/glucose 40 g/day; G80, fructose/glucose 80 g/day; G, glucose; S, sucrose; I, impaired glucose tolerance, N, normal glucose tolerance/body weight; O, overweight

Study or Subgroup	Mean Difference	SE	Weight	Mean Difference IV, Random, 95% CI
**3.2.1 BMI</= 25**				
Aeberli 2011 G40	−0.11	0.01	11.3%	−0.11 [−0.13, −0.09]
Aeberli 2011 G80	−0.04	0.04	10.0%	−0.04 [−0.12, 0.04]
Aeberli 2011 S80	−0.12	0.04	10.0%	−0.12 [−0.20, −0.04]
Aeberli 2013 G	0.09	0.02	11.0%	0.09 [0.05, 0.13]
Aeberli 2013 S	0.01	0.02	11.0%	0.01 [−0.03, 0.05]
Bossetti 1984	0.11	0.04	10.0%	0.11 [0.03, 0.19]
Heden 2014/1	−0.28	0.11	5.4%	−0.28 [−0.50, −0.06]
Heden 2014/3	−0.22	0.03	10.6%	−0.22 [−0.28, −0.16]
Koh 1988 N	−0.54	0.05	9.3%	−0.54 [−0.64, −0.44]
Kuzma 2019 N	−0.06	0.01	11.3%	−0.06 [−0.08, −0.04]
**Subtotal (95% CI)**			**100.0%**	**−0.10 [−0.17, −0.03]**
Heterogeneity: $Tau^2 = 0.01$; $Chi^2 = 236.35$, df = 9 (P < 0.00001); $I^2 = 96\%$				
Test for overall effect: Z = 2.89 (P = 0.004)				
**3.2.2 BMI 25.1-29.9**				
Angelopolous 2016	0.04	0.07	17.1%	0.04 [−0.10, 0.18]
Bantle 2000	0.08	0.05	17.3%	0.08 [−0.02, 0.18]
Koh 1988 I	−0.61	0.05	17.3%	−0.61 [−0.71, −0.51]
Lowndes 2015	−0.1	0.15	15.6%	−0.10 [−0.39, 0.19]
Malerbi 1996	−0.8	0.06	17.2%	−0.80 [−0.92, −0.68]
Silbernagel 2011	−0.06	0.16	15.4%	−0.06 [−0.37, 0.25]
**Subtotal (95% CI)**			**100.0%**	**−0.25 [−0.60, 0.10]**
Heterogeneity: $Tau^2 = 0.18$; $Chi^2 = 192.87$, df = 5 (P < 0.00001); $I^2 = 97\%$				
Test for overall effect: Z = 1.39 (P = 0.16)				
**3.2.3 30 and greater**				
Heden 2014/2	−0.05	0.02	19.5%	−0.05 [−0.09, −0.01]
Heden 2014/4	−0.05	0.03	19.3%	−0.05 [−0.11, 0.01]
Heden 2015	−0.04	0.06	18.3%	−0.04 [−0.16, 0.08]
Jin 2014	0	0.35	5.3%	0.00 [−0.69, 0.69]
Kuzma 2019 O	0.06	0.03	19.3%	0.06 [0.00, 0.12]
Malerbi 1996	−0.8	0.06	18.3%	−0.80 [−0.92, −0.68]
**Subtotal (95% CI)**			**100.0%**	**−0.16 [−0.35, 0.02]**
Heterogeneity: $Tau^2 = 0.05$; $Chi^2 = 168.44$, df = 5 (P < 0.00001); $I^2 = 97\%$				
Test for overall effect: Z = 1.71 (P = 0.09)				

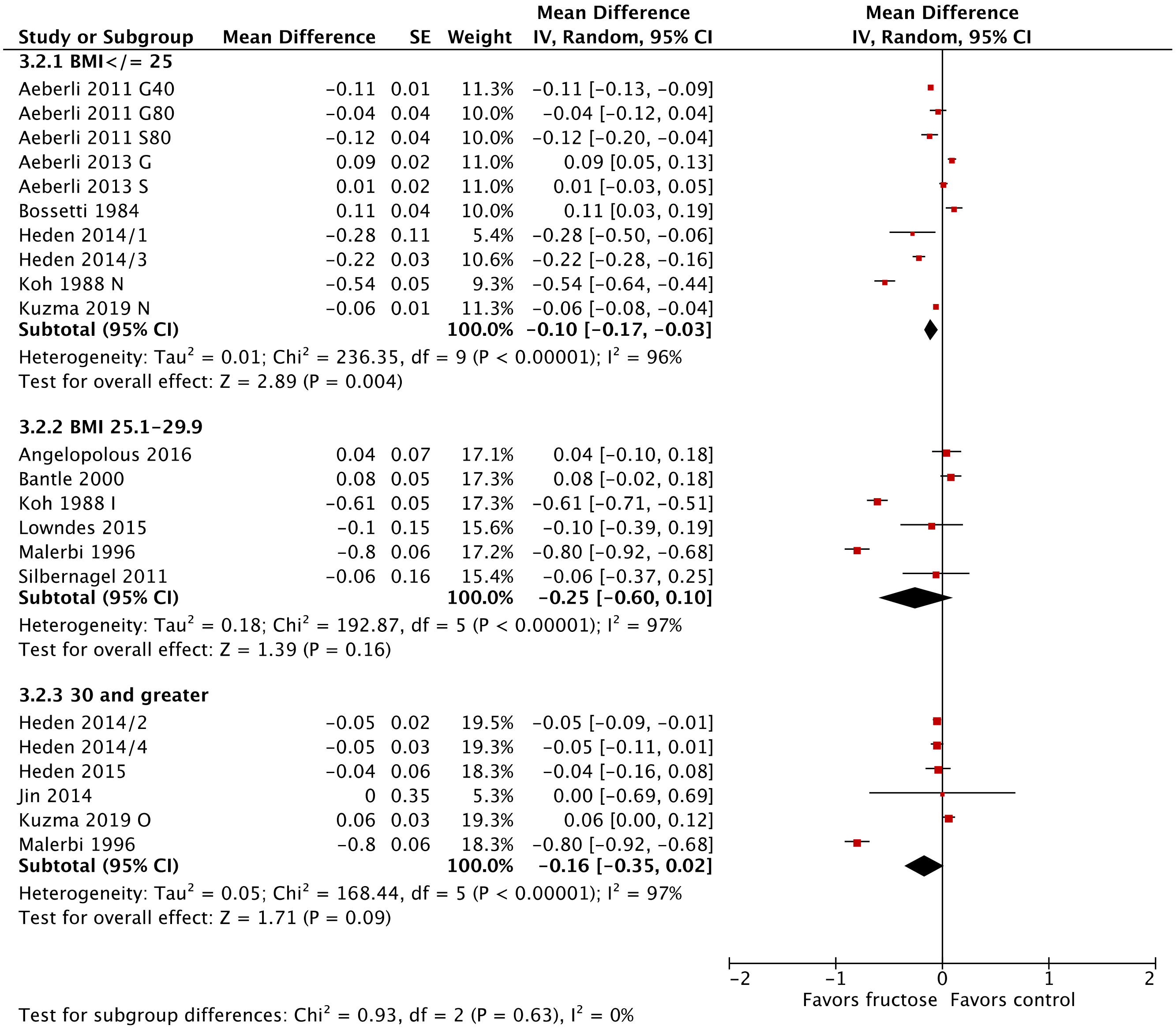

Test for subgroup differences: $Chi^2 = 0.93$, df = 2 (P = 0.63), $I^2 = 0\%$

Supplementary figure 4: Subgroup meta-analysis of fasting blood glucose following isoenergetic substitution of glucose or sucrose by fructose in food or beverages by baseline BMI. Values are mean differences [95% CIs] (expressed as mmol/L) between fasting blood glucose after fructose consumption and fasting blood glucose following glucose or sucrose consumption. IV, inverse variance; SE, standard error; G40, fructose/glucose 40 g/day; G80, fructose/glucose 80 g/day; G, glucose; S, sucrose; I, impaired glucose tolerance, N, normal glucose tolerance/body weight; O, overweight

Study or Subgroup	Std. Mean Difference	SE	Weight	Std. Mean Difference IV, Random, 95% CI
**1.1.1 Fructose vs glucose**				
Koh 1988 I	−3.0335	0.7413	41.2%	−3.03 [−4.49, −1.58]
Koh 1988 N	−2.1379	0.6203	58.8%	−2.14 [−3.35, −0.92]
**Subtotal (95% CI)**			**100.0%**	**−2.51 [−3.44, −1.57]**
Heterogeneity: Tau² = 0.00; Chi² = 0.86, df = 1 (P = 0.35); I² = 0%				
Test for overall effect: Z = 5.27 (P < 0.00001)				
**1.1.2 Fructose vs sucrose**				
Malerbi 1996	0	0.3536	100.0%	0.00 [−0.69, 0.69]
**Subtotal (95% CI)**			**100.0%**	**0.00 [−0.69, 0.69]**
Heterogeneity: Not applicable				
Test for overall effect: Z = 0.00 (P = 1.00)				
Test for subgroup differences: Chi² = 17.88, df = 1 (P < 0.0001), I² = 94.4%				

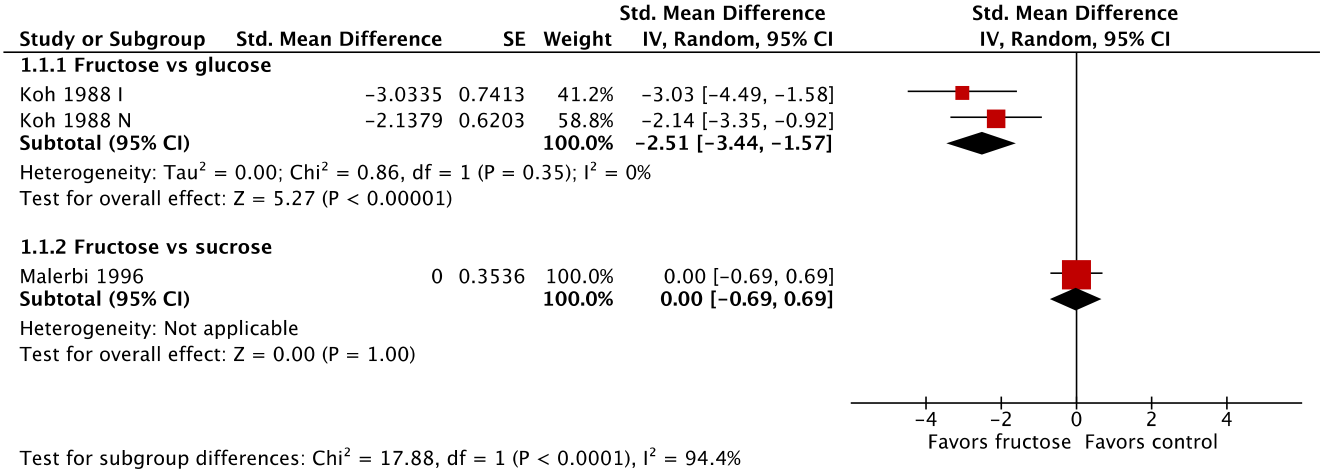

Supplementary figure 5: Subgroup meta-analysis of HbA1c following isoenergetic substitution of glucose or sucrose by fructose in food or beverages by substituted sugar. Values are standardized mean differences [95% CIs] (expressed as units) between HbA1c after fructose consumption and HbA1c following glucose or sucrose consumption. IV, inverse variance; SE, standard error; I, impaired glucose tolerance, N, body weight

Study or Subgroup	Std. Mean Difference	SE	Weight	Std. Mean Difference IV, Random, 95% CI
**2.1.1 Fructose vs glucose**				
Aeberli 2011 G40	0.1619	0.2631	10.0%	0.16 [−0.35, 0.68]
Aeberli 2011 G80	1.0363	0.2809	9.9%	1.04 [0.49, 1.59]
Heden 2014/1	0.5675	0.4834	7.6%	0.57 [−0.38, 1.51]
Heden 2014/2	1.5383	0.4973	7.5%	1.54 [0.56, 2.51]
Heden 2014/3	−0.6311	0.4391	8.1%	−0.63 [−1.49, 0.23]
Heden 2014/4	1.2222	0.5247	7.2%	1.22 [0.19, 2.25]
Heden 2015	−0.6944	0.5565	6.9%	−0.69 [−1.79, 0.40]
Jin 2014	0.2863	0.4437	8.1%	0.29 [−0.58, 1.16]
Kuzma 2019 N	−1.4078	0.4648	7.8%	−1.41 [−2.32, −0.50]
Kuzma 2019 O	0	0.4082	8.5%	0.00 [−0.80, 0.80]
Lowndes 2015	−0.3585	0.2189	10.5%	−0.36 [−0.79, 0.07]
Silbernagel 2011	−0.4181	0.4533	8.0%	−0.42 [−1.31, 0.47]
**Subtotal (95% CI)**			**100.0%**	**0.11 [−0.34, 0.56]**
Heterogeneity: Tau^2 = 0.45; Chi^2 = 46.30, df = 11 (P < 0.00001); I^2 = 76%				
Test for overall effect: Z = 0.47 (P = 0.64)				
**2.1.2 Fructose vs sucrose**				
Aeberli 2011 S80	0.7772	0.273	100.0%	0.78 [0.24, 1.31]
**Subtotal (95% CI)**			**100.0%**	**0.78 [0.24, 1.31]**
Heterogeneity: Not applicable				
Test for overall effect: Z = 2.85 (P = 0.004)				

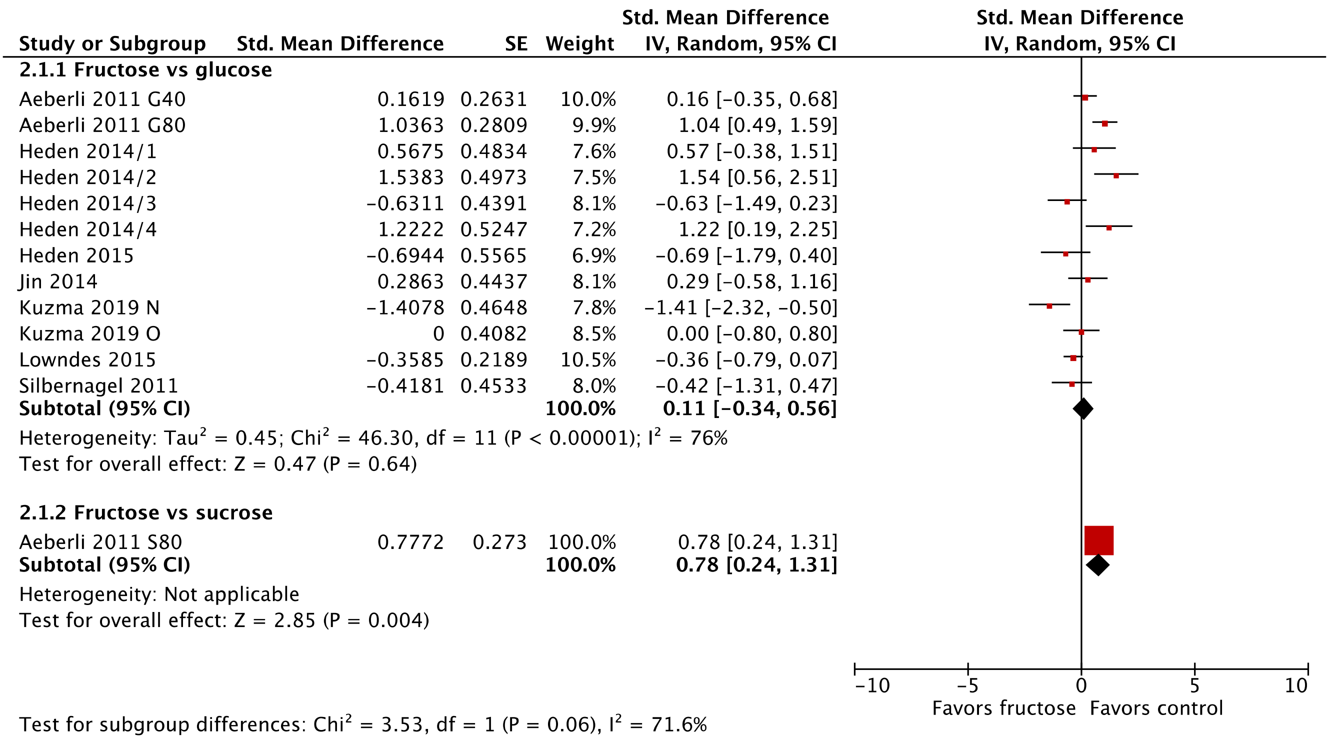

Test for subgroup differences: Chi^2 = 3.53, df = 1 (P = 0.06), I^2 = 71.6%

Supplementary figure 6: Subgroup meta-analysis of HOMA-IR/HOMA2 following isoenergetic substitution of glucose or sucrose by fructose in food or beverages by substituted sugar. Values are standardized mean differences [95% CIs] (expressed as units) between HOMA-IR or HOMA2 after fructose consumption and HOMA-IR or HOMA2 following glucose or sucrose consumption. IV, inverse variance; SE, standard error; G40, fructose/glucose 40 g/day; G80, fructose/glucose 80 g/day; N, normal body weight; O, overweight

Study or Subgroup	Std. Mean Difference	SE	Weight	Std. Mean Difference IV, Random, 95% CI
**2.2.1 BMI </= 25**				
Aeberli 2011 G40	0.1619	0.2631	18.5%	0.16 [−0.35, 0.68]
Aeberli 2011 G80	1.0363	0.2809	18.2%	1.04 [0.49, 1.59]
Aeberli 2011 S80	0.7772	0.273	18.4%	0.78 [0.24, 1.31]
Heden 2014/1	0.5687	0.4834	14.6%	0.57 [−0.38, 1.52]
Heden 2014/3	−0.6311	0.4391	15.4%	−0.63 [−1.49, 0.23]
Kuzma 2019 N	−1.4078	0.4648	14.9%	−1.41 [−2.32, −0.50]
**Subtotal (95% CI)**			**100.0%**	**0.14 [−0.52, 0.80]**
Heterogeneity: $Tau^2 = 0.54$; $Chi^2 = 28.42$, df = 5 ($P < 0.0001$); $I^2 = 82\%$				
Test for overall effect: Z = 0.41 (P = 0.68)				
**2.2.2 BMI 25.1−29.9**				
Heden 2014/2	1.5383	0.4973	13.1%	1.54 [0.56, 2.51]
Heden 2014/4	1.2222	0.5247	12.6%	1.22 [0.19, 2.25]
Heden 2015	−0.6944	0.5565	12.0%	−0.69 [−1.79, 0.40]
Jin 2014	0.2863	0.4437	14.2%	0.29 [−0.58, 1.16]
Kuzma 2019 O	0	0.4082	15.0%	0.00 [−0.80, 0.80]
Lowndes 2015	−0.3585	0.2189	19.0%	−0.36 [−0.79, 0.07]
Silbernagel 2011	−0.4181	0.4533	14.0%	−0.42 [−1.31, 0.47]
**Subtotal (95% CI)**			**100.0%**	**0.19 [−0.39, 0.76]**
Heterogeneity: $Tau^2 = 0.40$; $Chi^2 = 20.50$, df = 6 (P = 0.002); $I^2 = 71\%$				
Test for overall effect: Z = 0.64 (P = 0.52)				
**2.2.3 30 and greater**				
**Subtotal (95% CI)**				**Not estimable**
Heterogeneity: Not applicable				
Test for overall effect: Not applicable				

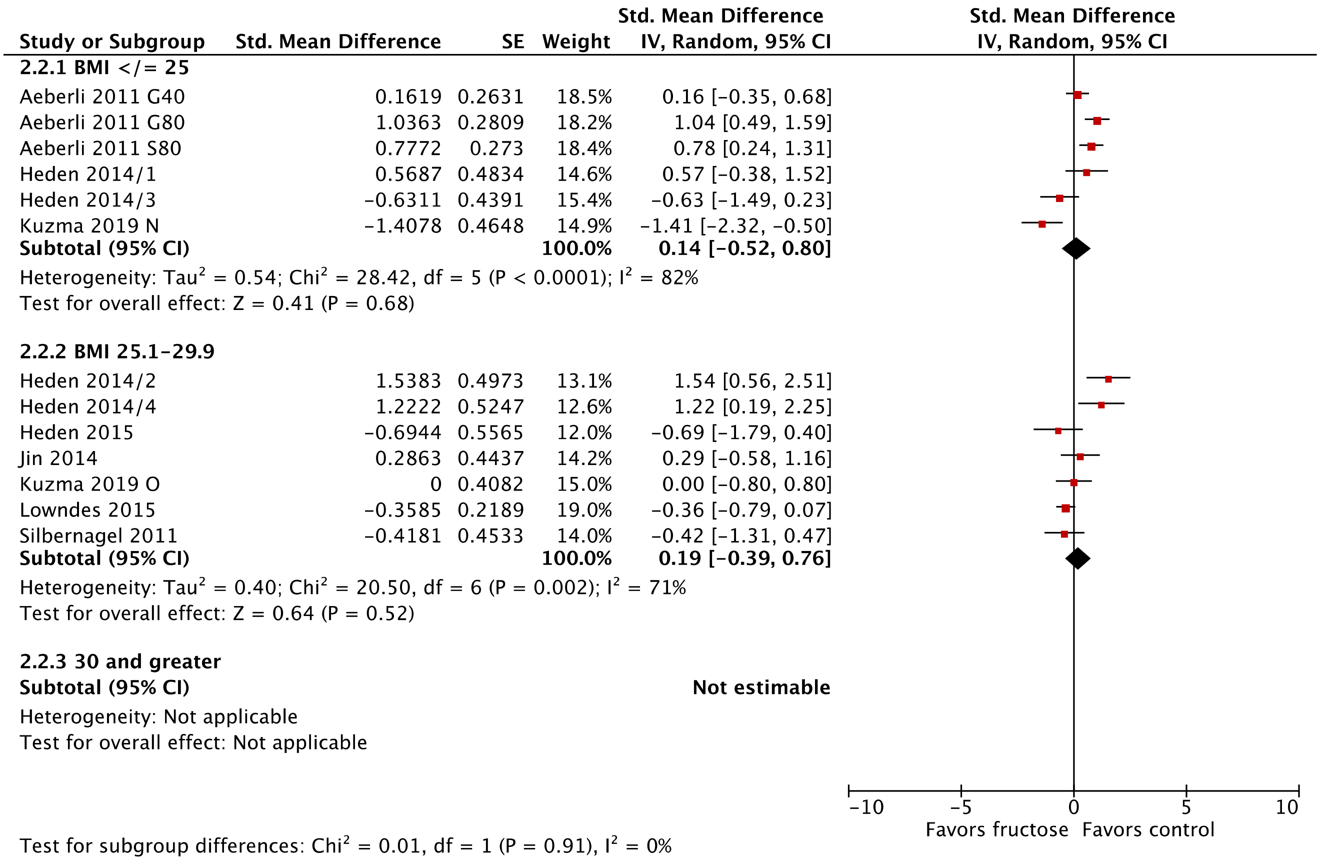

Test for subgroup differences: $Chi^2 = 0.01$, df = 1 (P = 0.91), $I^2 = 0\%$

Supplementary figure 7: Subgroup meta-analysis of HOMA-IR/HOMA2 following isoenergetic substitution of glucose or sucrose by fructose in food or beverages by baseline BMI. Values are standardized mean differences [95% CIs] (expressed as units) between HOMA-IR or HOMA2 after fructose consumption and HOMA-IR or HOMA2 following glucose or sucrose consumption. IV, inverse variance; SE, standard error; G40, fructose/glucose 40 g/day; G80, fructose/glucose 80 g/day; N, normal body weight; O, overweight

Study or Subgroup	Std. Mean Difference	SE	Weight	Std. Mean Difference IV, Random, 95% CI
**2.3.1 30-40 grams/day**				
Aeberli 2011 G40	0.1619	0.2631	21.0%	0.16 [−0.35, 0.68]
Heden 2014/1	0.5675	0.4834	16.2%	0.57 [−0.38, 1.51]
Heden 2014/2	1.5383	0.4973	15.8%	1.54 [0.56, 2.51]
Heden 2014/3	−0.6311	0.4391	17.1%	−0.63 [−1.49, 0.23]
Heden 2014/4	1.2222	0.5247	15.3%	1.22 [0.19, 2.25]
Heden 2015	−0.6944	0.5565	14.6%	−0.69 [−1.79, 0.40]
**Subtotal (95% CI)**			**100.0%**	**0.35 [−0.32, 1.01]**
Heterogeneity: $Tau^2 = 0.48$; $Chi^2 = 17.55$, df = 5 (P = 0.004); $I^2 = 72\%$				
Test for overall effect: Z = 1.02 (P = 0.31)				
**2.3.2 41-80 g/day**				
Aeberli 2011 G80	1.0363	0.2809	32.7%	1.04 [0.49, 1.59]
Aeberli 2011 S80	0.7772	0.273	32.9%	0.78 [0.24, 1.31]
Lowndes 2015	−0.3585	0.2189	34.4%	−0.36 [−0.79, 0.07]
**Subtotal (95% CI)**			**100.0%**	**0.47 [−0.42, 1.36]**
Heterogeneity: $Tau^2 = 0.56$; $Chi^2 = 18.89$, df = 2 (P < 0.0001); $I^2 = 89\%$				
Test for overall effect: Z = 1.04 (P = 0.30)				
**2.3.3 >80 g/day**				
Jin 2014	0.2863	0.4437	24.9%	0.29 [−0.58, 1.16]
Kuzma 2019 N	−1.4078	0.4648	24.1%	−1.41 [−2.32, −0.50]
Kuzma 2019 O	0	0.4082	26.5%	0.00 [−0.80, 0.80]
Silbernagel 2011	−0.4181	0.4533	24.5%	−0.42 [−1.31, 0.47]
**Subtotal (95% CI)**			**100.0%**	**−0.37 [−1.08, 0.34]**
Heterogeneity: $Tau^2 = 0.32$; $Chi^2 = 7.99$, df = 3 (P = 0.05); $I^2 = 62\%$				
Test for overall effect: Z = 1.03 (P = 0.31)				

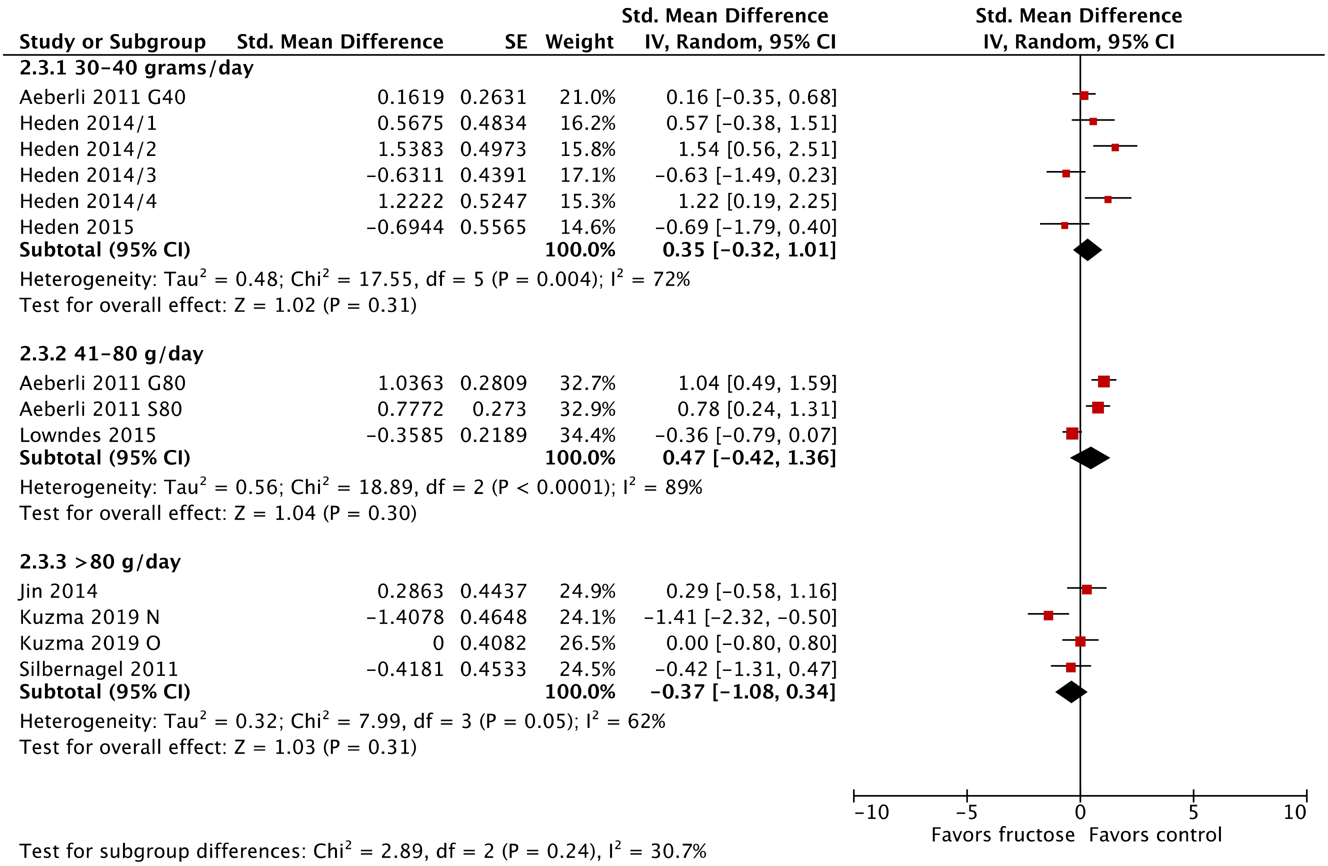

Test for subgroup differences: $Chi^2 = 2.89$, df = 2 (P = 0.24), $I^2 = 30.7\%$

Supplementary figure 8: Subgroup meta-analysis of HOMA-IR/HOMA2 following isoenergetic substitution of glucose or sucrose by fructose in food or beverages by dose of sugar. Values are standardized mean differences [95% CIs] (expressed as units) between HOMA-IR or HOMA2 after fructose consumption and HOMA-IR or HOMA2 following glucose or sucrose consumption. IV, inverse variance; SE, standard error; G40, fructose/glucose 40 g/day; G80, fructose/glucose 80 g/day; N, normal body weight; O, overweight

Study or Subgroup	Std. Mean Difference	SE	Weight	Std. Mean Difference IV, Random, 95% CI
**2.4.1 No diabetes**				
Aeberli 2011 G40	0.1619	0.2631	9.2%	0.16 [−0.35, 0.68]
Aeberli 2011 G80	1.0363	0.2809	9.0%	1.04 [0.49, 1.59]
Aeberli 2011 S80	0.7772	0.273	9.1%	0.78 [0.24, 1.31]
Heden 2014/1	0.5675	0.4834	6.9%	0.57 [−0.38, 1.51]
Heden 2014/2	1.5383	0.4973	6.8%	1.54 [0.56, 2.51]
Heden 2014/3	−0.6311	0.4391	7.4%	−0.63 [−1.49, 0.23]
Heden 2014/4	1.2222	0.5247	6.5%	1.22 [0.19, 2.25]
Heden 2015	−0.6944	0.5565	6.2%	−0.69 [−1.79, 0.40]
Jin 2014	0.2863	0.4437	7.3%	0.29 [−0.58, 1.16]
Kuzma 2019 N	−1.4078	0.4648	7.1%	−1.41 [−2.32, −0.50]
Kuzma 2019 O	0	0.4082	7.7%	0.00 [−0.80, 0.80]
Lowndes 2015	−0.3585	0.2189	9.6%	−0.36 [−0.79, 0.07]
Silbernagel 2011	−0.4181	0.4533	7.2%	−0.42 [−1.31, 0.47]
**Subtotal (95% CI)**			**100.0%**	**0.17 [−0.25, 0.59]**
Heterogeneity: $Tau^2 = 0.44$; $Chi^2 = 51.72$, df = 12 (P < 0.00001); $I^2 = 77\%$				
Test for overall effect: Z = 0.78 (P = 0.43)				
**2.4.2 Impaired glucose tolerance**				
**Subtotal (95% CI)**				**Not estimable**
Heterogeneity: Not applicable				
Test for overall effect: Not applicable				
**2.4.4 Type 2 diabetes**				
**Subtotal (95% CI)**				**Not estimable**
Heterogeneity: Not applicable				
Test for overall effect: Not applicable				

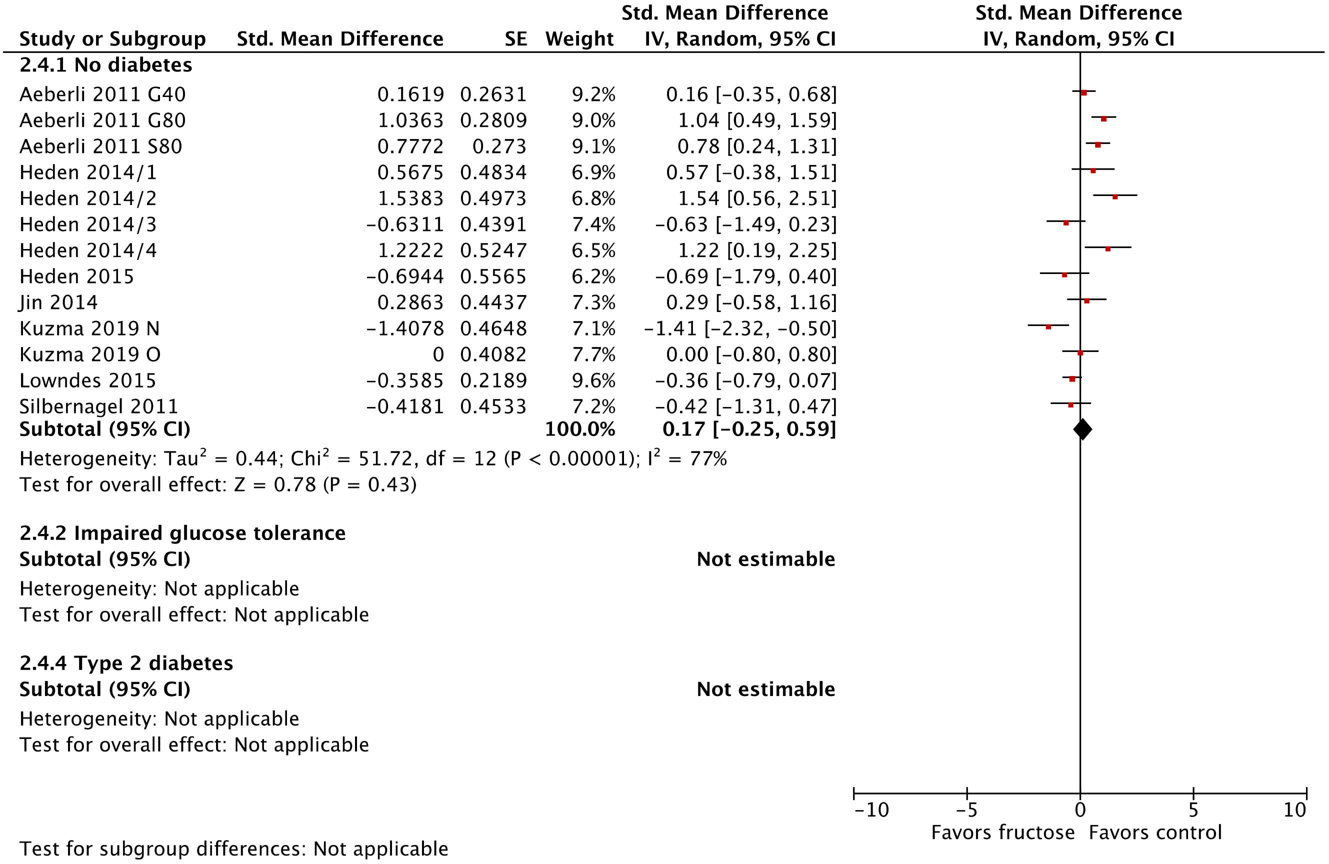

Test for subgroup differences: Not applicable

Supplementary figure 9: Subgroup meta-analysis of HOMA-IR/HOMA2 following isoenergetic substitution of glucose or sucrose by fructose in food or beverages by diabetes status. Values are standardized mean differences [95% CIs] (expressed as units) between HOMA-IR or HOMA2 after fructose consumption and HOMA-IR or HOMA2 following glucose or sucrose consumption. IV, inverse variance; SE, standard error; G40, fructose/glucose 40 g/day; G80, fructose/glucose 80 g/day; N, normal body weight; O, overweight

Study or Subgroup	Mean Difference	SE	Weight	Mean Difference IV, Random, 95% CI
**5.3.1 30-40 g/day**				
Heden 2014/1	0.43	0.93	21.3%	0.43 [-1.39, 2.25]
Heden 2014/2	-0.72	1.66	6.7%	-0.72 [-3.97, 2.53]
Heden 2014/3	-1.44	0.55	60.8%	-1.44 [-2.52, -0.36]
Heden 2014/4	-1.01	1.49	8.3%	-1.01 [-3.93, 1.91]
Heden 2015	-2.72	2.47	3.0%	-2.72 [-7.56, 2.12]
**Subtotal (95% CI)**			**100.0%**	**-1.00 [-1.84, -0.16]**
Heterogeneity: Tau² = 0.00; Chi² = 3.52, df = 4 (P = 0.48); I² = 0%				
Test for overall effect: Z = 2.33 (P = 0.02)				
**5.3.2 41-80 g/day**				
Aeberli 2011 S80	1.26	2.96	2.7%	1.26 [-4.54, 7.06]
Aeberli 2013 G	1.21	2.25	4.7%	1.21 [-3.20, 5.62]
Bossetti 1984	-1.8	4.68	1.1%	-1.80 [-10.97, 7.37]
Koh 1988 I	-0.31	0.75	42.7%	-0.31 [-1.78, 1.16]
Koh 1988 N	-0.44	0.75	42.7%	-0.44 [-1.91, 1.03]
Lowndes 2015	3.21	2	6.0%	3.21 [-0.71, 7.13]
**Subtotal (95% CI)**			**100.0%**	**-0.06 [-1.02, 0.91]**
Heterogeneity: Tau² = 0.00; Chi² = 3.70, df = 5 (P = 0.59); I² = 0%				
Test for overall effect: Z = 0.11 (P = 0.91)				
**5.3.3 >80 g/day**				
Jin 2014	15.6	11.11	0.2%	15.60 [-6.18, 37.38]
Kuzma 2019 N	-2	0.56	55.6%	-2.00 [-3.10, -0.90]
Kuzma 2019 O	-0.3	1.02	23.1%	-0.30 [-2.30, 1.70]
Malerbi 1996	-1.73	1.53	11.3%	-1.73 [-4.73, 1.27]
Silbernagel 2011	-1.58	1.65	9.8%	-1.58 [-4.81, 1.65]
**Subtotal (95% CI)**			**100.0%**	**-1.49 [-2.55, -0.44]**
Heterogeneity: Tau² = 0.20; Chi² = 4.54, df = 4 (P = 0.34); I² = 12%				
Test for overall effect: Z = 2.79 (P = 0.005)				

Test for subgroup differences: Chi² = 4.19, df = 2 (P = 0.12), I² = 52.3%

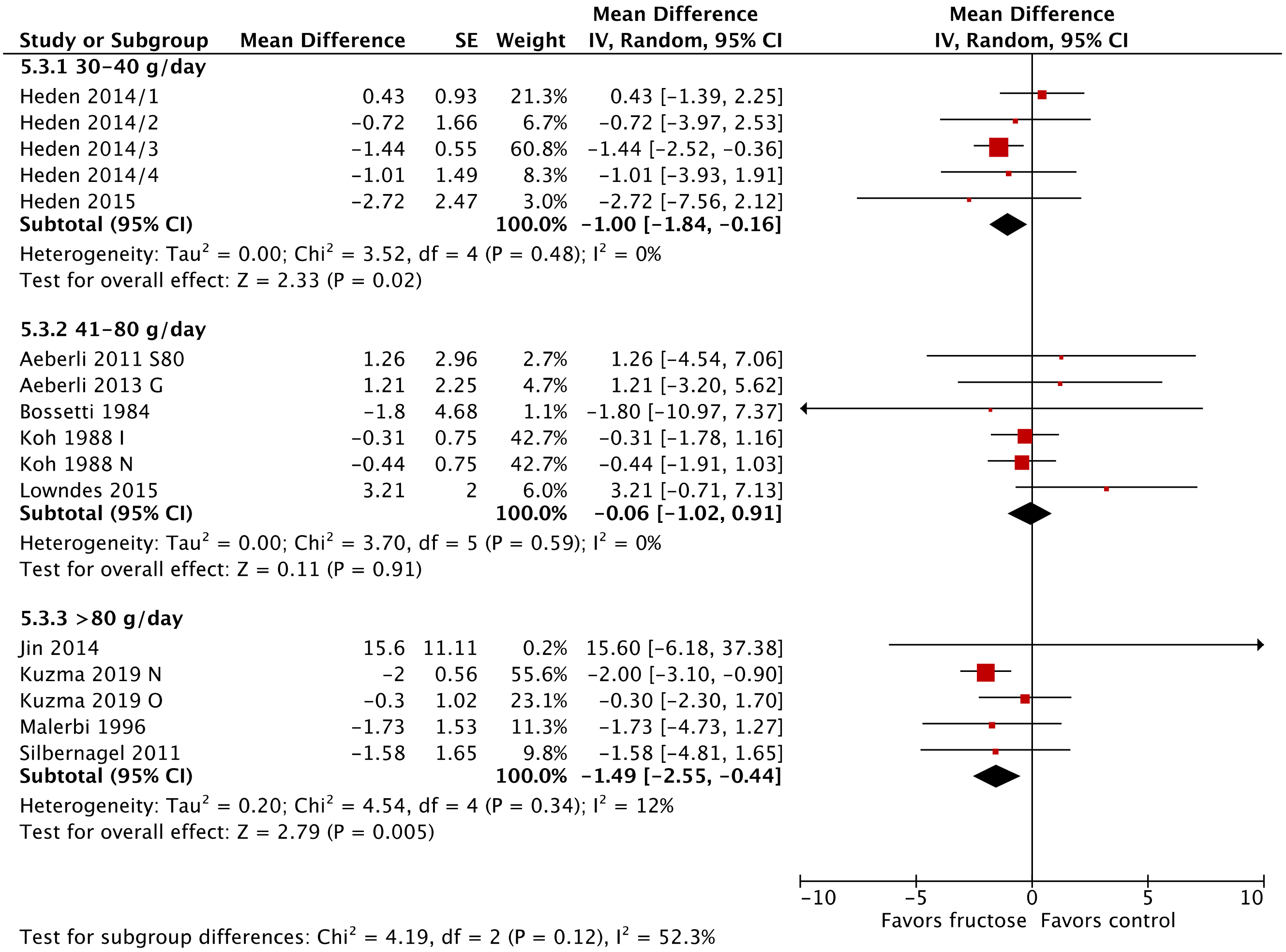

Supplementary figure 10: Subgroup meta-analysis of fasting blood insulin following isoenergetic substitution of glucose or sucrose by fructose in food or beverages by dose of sugar. Values are mean differences [95% CIs] (expressed as µIU/mL) between fasting blood insulin after fructose consumption and fasting blood insulin following glucose or sucrose consumption. IV, inverse variance; SE, standard error; G40, fructose/glucose 40 g/day; G80, fructose/glucose 80 g/day; G, glucose; S, sucrose; I, impaired glucose tolerance, N, normal glucose tolerance/body weight; O, overweight

Study or Subgroup	Mean Difference	SE	Weight	Mean Difference IV, Random, 95% CI
**5.4.1 No diabetes**				
Aeberli 2011 S80	1.26	2.96	1.4%	1.26 [-4.54, 7.06]
Aeberli 2013 G	1.21	2.25	2.4%	1.21 [-3.20, 5.62]
Bossetti 1984	-1.8	4.68	0.6%	-1.80 [-10.97, 7.37]
Heden 2014/1	0.43	0.93	11.0%	0.43 [-1.39, 2.25]
Heden 2014/2	-0.72	1.66	4.2%	-0.72 [-3.97, 2.53]
Heden 2014/3	-1.44	0.55	21.1%	-1.44 [-2.52, -0.36]
Heden 2014/4	-1.01	1.49	5.1%	-1.01 [-3.93, 1.91]
Heden 2015	-2.72	2.47	2.0%	-2.72 [-7.56, 2.12]
Jin 2014	15.6	11.11	0.1%	15.60 [-6.18, 37.38]
Koh 1988 N	-0.44	0.75	14.8%	-0.44 [-1.91, 1.03]
Kuzma 2019 N	-2	0.56	20.7%	-2.00 [-3.10, -0.90]
Kuzma 2019 O	-0.3	1.02	9.5%	-0.30 [-2.30, 1.70]
Lowndes 2015	3.21	2	3.0%	3.21 [-0.71, 7.13]
Silbernagel 2011	-1.58	1.65	4.2%	-1.58 [-4.81, 1.65]
**Subtotal (95% CI)**			**100.0%**	**-0.82 [-1.52, -0.12]**
Heterogeneity: $Tau^2 = 0.31$; $Chi^2 = 16.10$, df = 13 (P = 0.24); $I^2 = 19\%$				
Test for overall effect: Z = 2.29 (P = 0.02)				
**5.4.2 Impaired glucose tolerance**				
Koh 1988 I	-0.31	0.75	100.0%	-0.31 [-1.78, 1.16]
**Subtotal (95% CI)**			**100.0%**	**-0.31 [-1.78, 1.16]**
Heterogeneity: Not applicable				
Test for overall effect: Z = 0.41 (P = 0.68)				
**5.4.4 Type 2 diabetes**				
Malerbi 1996	-1.73	1.53	100.0%	-1.73 [-4.73, 1.27]
**Subtotal (95% CI)**			**100.0%**	**-1.73 [-4.73, 1.27]**
Heterogeneity: Not applicable				
Test for overall effect: Z = 1.13 (P = 0.26)				

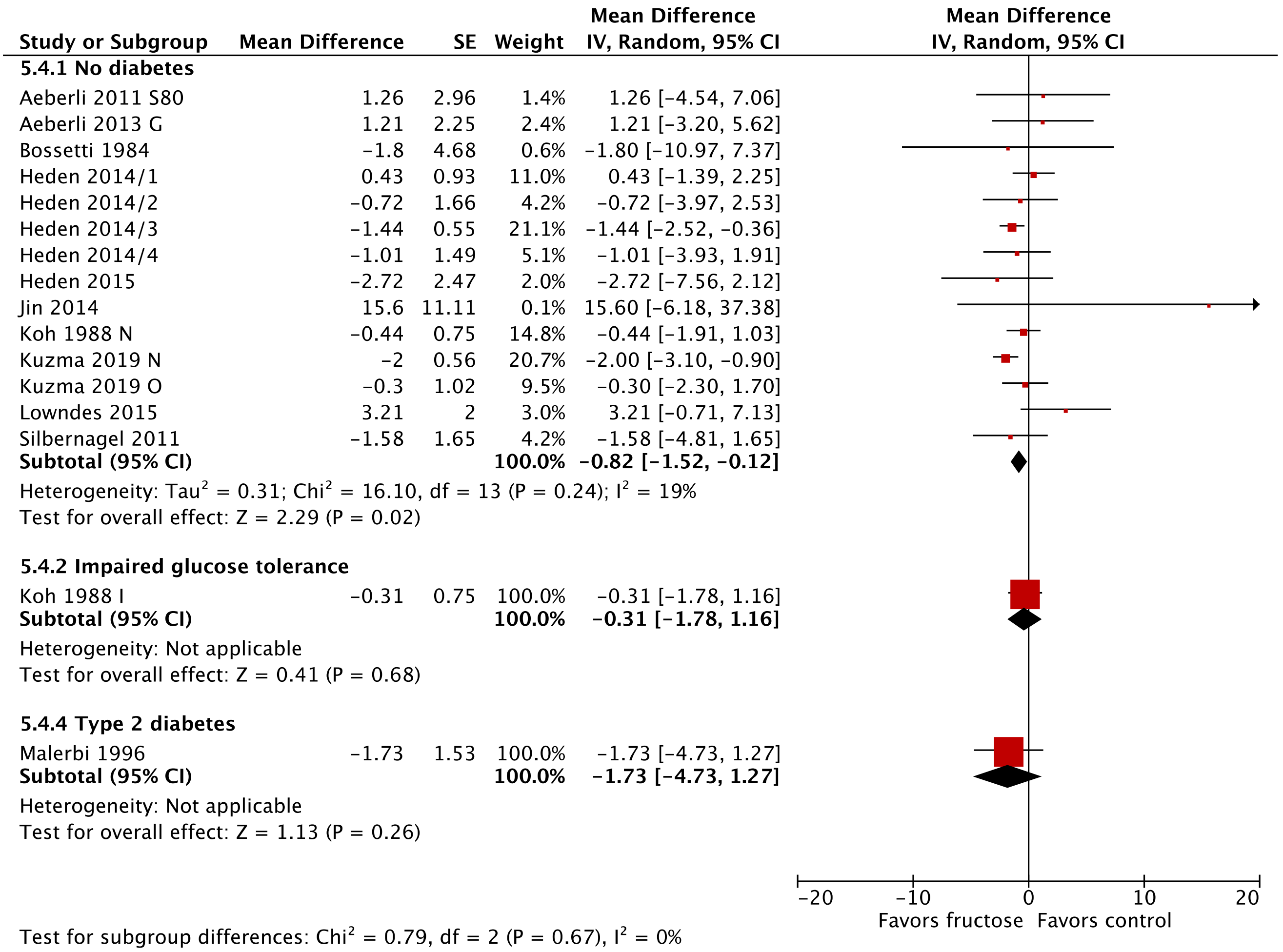

Test for subgroup differences: $Chi^2 = 0.79$, df = 2 (P = 0.67), $I^2 = 0\%$

Supplementary figure 11: Subgroup meta-analysis of fasting blood insulin following isoenergetic substitution of glucose or sucrose by fructose in food or beverages by dose of sugar. Values are mean differences [95% CIs] (expressed as µIU/mL) between fasting blood insulin after fructose consumption and fasting blood insulin following glucose or sucrose consumption. IV, inverse variance; SE, standard error; G40, fructose/glucose 40 g/day; G80, fructose/glucose 80 g/day; G, glucose; S, sucrose; I, impaired glucose tolerance, N, normal glucose tolerance/body weight; O, overweight

Study or Subgroup	Mean Difference	SE	Weight	Mean Difference IV, Random, 95% CI
**5.2.1 BMI </= 25**				
Aeberli 2013 G	1.21	0.75	16.4%	1.21 [-0.26, 2.68]
Aeberli 2013 S	1.26	0.99	14.6%	1.26 [-0.68, 3.20]
Bossetti 1984	-1.8	4.68	2.2%	-1.80 [-10.97, 7.37]
Heden 2014/1	0.43	0.93	15.0%	0.43 [-1.39, 2.25]
Heden 2014/3	-1.44	0.55	17.7%	-1.44 [-2.52, -0.36]
Koh 1988 N	-3.8	0.75	16.4%	-3.80 [-5.27, -2.33]
Kuzma 2019 N	-2	0.56	17.7%	-2.00 [-3.10, -0.90]
**Subtotal (95% CI)**			**100.0%**	**-0.82 [-2.28, 0.63]**
Heterogeneity: $Tau^2 = 2.82$; $Chi^2 = 33.80$, df = 6 (P < 0.00001); $I^2 = 82\%$				
Test for overall effect: Z = 1.11 (P = 0.27)				
**5.2.2 BMI 25.1-29.9**				
Koh 1988 I	-2.58	0.75	36.3%	-2.58 [-4.05, -1.11]
Lowndes 2015	3.21	2	17.9%	3.21 [-0.71, 7.13]
Malerbi 1996	-1.73	1.53	23.7%	-1.73 [-4.73, 1.27]
Silbernagel 2011	-1.58	1.65	22.1%	-1.58 [-4.81, 1.65]
**Subtotal (95% CI)**			**100.0%**	**-1.12 [-3.28, 1.04]**
Heterogeneity: $Tau^2 = 2.79$; $Chi^2 = 7.38$, df = 3 (P = 0.06); $I^2 = 59\%$				
Test for overall effect: Z = 1.02 (P = 0.31)				
**5.2.3 30 and greater**				
Heden 2014/2	-0.72	1.66	18.6%	-0.72 [-3.97, 2.53]
Heden 2014/4	-1.01	1.49	23.1%	-1.01 [-3.93, 1.91]
Heden 2015	-2.72	2.47	8.4%	-2.72 [-7.56, 2.12]
Jin 2014	15.6	11.11	0.4%	15.60 [-6.18, 37.38]
Kuzma 2019 O	-0.3	1.02	49.4%	-0.30 [-2.30, 1.70]
**Subtotal (95% CI)**			**100.0%**	**-0.68 [-2.09, 0.72]**
Heterogeneity: $Tau^2 = 0.00$; $Chi^2 = 3.02$, df = 4 (P = 0.55); $I^2 = 0\%$				
Test for overall effect: Z = 0.95 (P = 0.34)				

Test for subgroup differences: $Chi^2 = 0.11$, df = 2 (P = 0.95), $I^2 = 0\%$

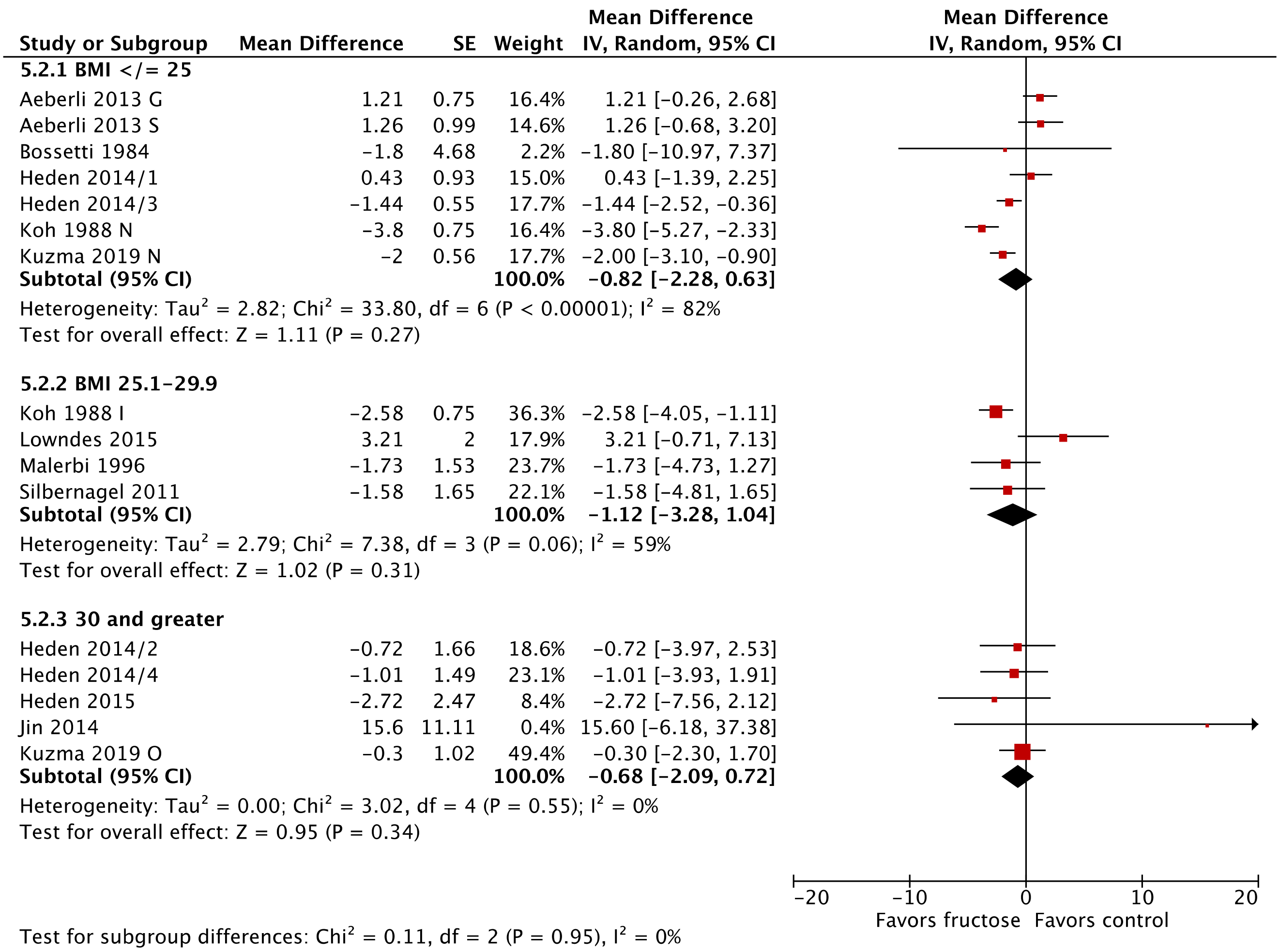

Supplementary figure 12: Subgroup meta-analysis of fasting blood insulin following isoenergetic substitution of glucose or sucrose by fructose in food or beverages by baseline BMI. Values are mean differences [95% CIs] (expressed as µIU/mL) between fasting blood insulin after fructose consumption and fasting blood insulin following glucose or sucrose consumption. IV, inverse variance; SE, standard error; G40, fructose/glucose 40 g/day; G80, fructose/glucose 80 g/day; G, glucose; S, sucrose; I, impaired glucose tolerance, N, normal glucose tolerance/body weight; O, overweight

Study or Subgroup	Mean Difference	SE	Weight	Mean Difference IV, Random, 95% CI
**6.2.1 BMI </= 25**				
Aeberli 2011 S80	−0.04	0.32	5.1%	−0.04 [−0.67, 0.59]
Aeberli 2013 G	0.24	0.29	6.0%	0.24 [−0.33, 0.81]
Bossetti 1984	0.1	0.1	20.0%	0.10 [−0.10, 0.30]
Heden 2014/1	−0.03	0.08	22.5%	−0.03 [−0.19, 0.13]
Heden 2014/3	−0.36	0.09	21.3%	−0.36 [−0.54, −0.18]
Koh 1988 N	−0.18	0.06	25.0%	−0.18 [−0.30, −0.06]
**Subtotal (95% CI)**			**100.0%**	**−0.10 [−0.25, 0.06]**
Heterogeneity: $Tau^2 = 0.02$; $Chi^2 = 15.78$, df = 5 (P = 0.007); $I^2 = 68\%$				
Test for overall effect: Z = 1.20 (P = 0.23)				
**6.2.2 BMI 25.1 - 29.9**				
Angelopolous 2016	0.12	0.15	9.7%	0.12 [−0.17, 0.41]
Bantle 2000	0.08	0.02	49.4%	0.08 [0.04, 0.12]
Koh 1988 I	−0.05	0.14	10.9%	−0.05 [−0.32, 0.22]
Malerbi 1996	−0.1	0.08	23.5%	−0.10 [−0.26, 0.06]
Silbernagel 2011	0.26	0.19	6.5%	0.26 [−0.11, 0.63]
**Subtotal (95% CI)**			**100.0%**	**0.04 [−0.06, 0.14]**
Heterogeneity: $Tau^2 = 0.01$; $Chi^2 = 6.61$, df = 4 (P = 0.16); $I^2 = 39\%$				
Test for overall effect: Z = 0.76 (P = 0.45)				
**6.2.3 BMI 30 and greater**				
Heden 2014/2	0	0.08	26.0%	0.00 [−0.16, 0.16]
Heden 2014/4	−0.21	0.09	22.5%	−0.21 [−0.39, −0.03]
Heden 2015	−0.02	0.03	51.5%	−0.02 [−0.08, 0.04]
**Subtotal (95% CI)**			**100.0%**	**−0.06 [−0.16, 0.05]**
Heterogeneity: $Tau^2 = 0.00$; $Chi^2 = 4.22$, df = 2 (P = 0.12); $I^2 = 53\%$				
Test for overall effect: Z = 1.07 (P = 0.28)				

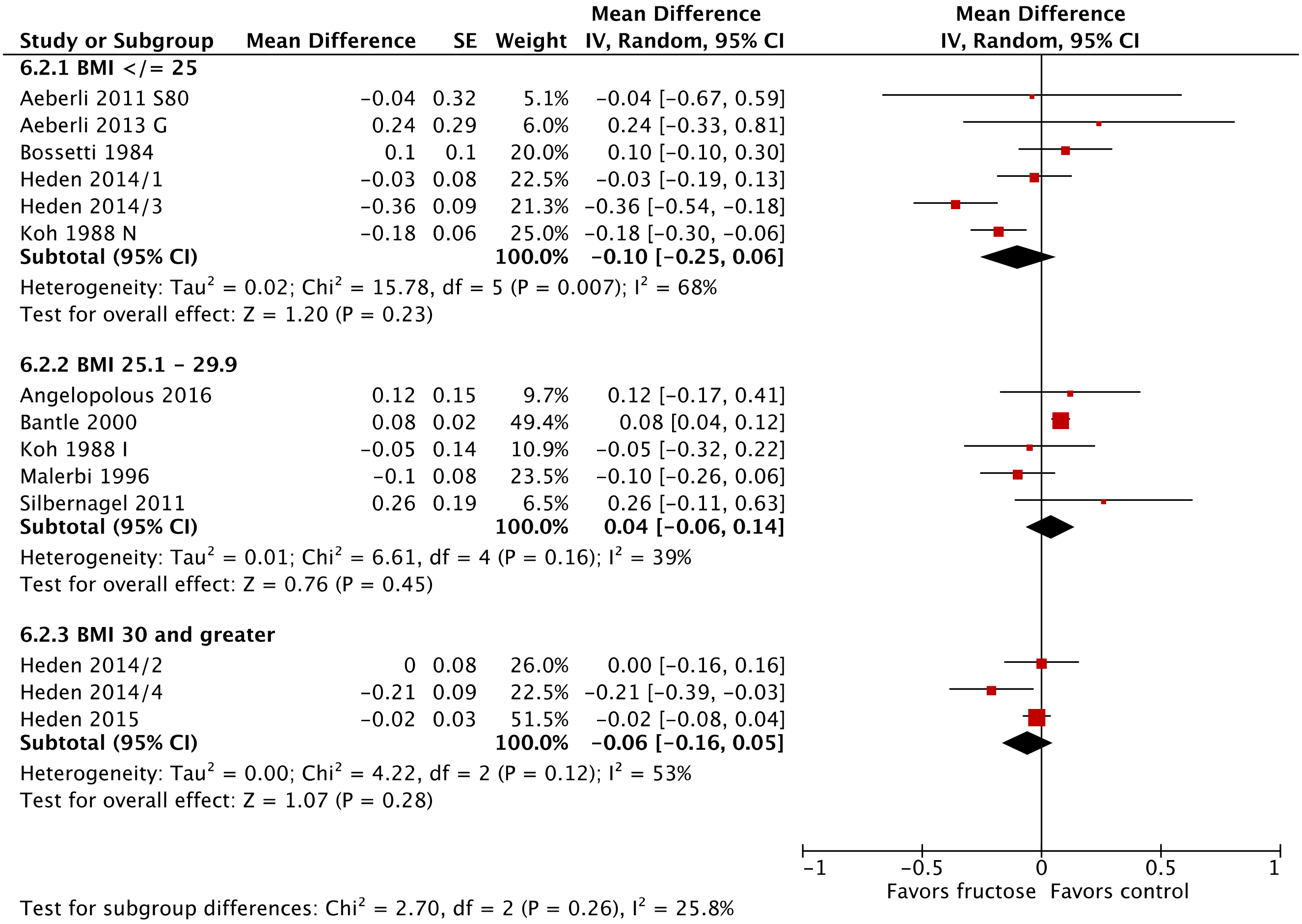

Test for subgroup differences: $Chi^2 = 2.70$, df = 2 (P = 0.26), $I^2 = 25.8\%$

Supplementary figure 13: Subgroup meta-analysis of fasting blood total cholesterol following isoenergetic substitution of glucose or sucrose by fructose in food or beverages by baseline BMI. Values are mean differences [95% CIs] (expressed as mmol/L) between fasting blood total cholesterol after fructose consumption and fasting blood total cholesterol following glucose or sucrose consumption. IV, inverse variance; SE, standard error; G40, fructose/glucose 40 g/day; G80, fructose/glucose 80 g/day; G, glucose; S, sucrose; I, impaired glucose tolerance, N, normal glucose tolerance/body weight; O, overweight

Study or Subgroup	Mean Difference	SE	Weight	Mean Difference IV, Random, 95% CI
**6.3.1 30-40 grams/day**				
Heden 2014/1	-0.03	0.08	19.2%	-0.03 [-0.19, 0.13]
Heden 2014/2	0	0.08	19.2%	0.00 [-0.16, 0.16]
Heden 2014/3	-0.36	0.09	17.7%	-0.36 [-0.54, -0.18]
Heden 2014/4	-0.21	0.09	17.7%	-0.21 [-0.39, -0.03]
Heden 2015	-0.02	0.03	26.3%	-0.02 [-0.08, 0.04]
**Subtotal (95% CI)**			**100.0%**	**-0.11 [-0.23, 0.01]**
Heterogeneity: Tau² = 0.01; Chi² = 16.36, df = 4 (P = 0.003); I² = 76%				
Test for overall effect: Z = 1.79 (P = 0.07)				
**6.3.2 41-80 g/day**				
Aeberli 2011 S80	-0.04	0.32	3.5%	-0.04 [-0.67, 0.59]
Aeberli 2013 G	0.24	0.29	4.2%	0.24 [-0.33, 0.81]
Angelopolous 2016	0.12	0.15	11.3%	0.12 [-0.17, 0.41]
Bantle 2000	0.08	0.02	28.4%	0.08 [0.04, 0.12]
Bossetti 1984	0.1	0.1	17.1%	0.10 [-0.10, 0.30]
Koh 1988 I	-0.05	0.14	12.2%	-0.05 [-0.32, 0.22]
Koh 1988 N	-0.18	0.06	23.3%	-0.18 [-0.30, -0.06]
**Subtotal (95% CI)**			**100.0%**	**0.01 [-0.11, 0.14]**
Heterogeneity: Tau² = 0.01; Chi² = 18.35, df = 6 (P = 0.005); I² = 67%				
Test for overall effect: Z = 0.22 (P = 0.83)				
**6.3.3 >80 g/day**				
Malerbi 1996	-0.1	0.08	61.5%	-0.10 [-0.26, 0.06]
Silbernagel 2011	0.26	0.19	38.5%	0.26 [-0.11, 0.63]
**Subtotal (95% CI)**			**100.0%**	**0.04 [-0.30, 0.38]**
Heterogeneity: Tau² = 0.04; Chi² = 3.05, df = 1 (P = 0.08); I² = 67%				
Test for overall effect: Z = 0.22 (P = 0.82)				

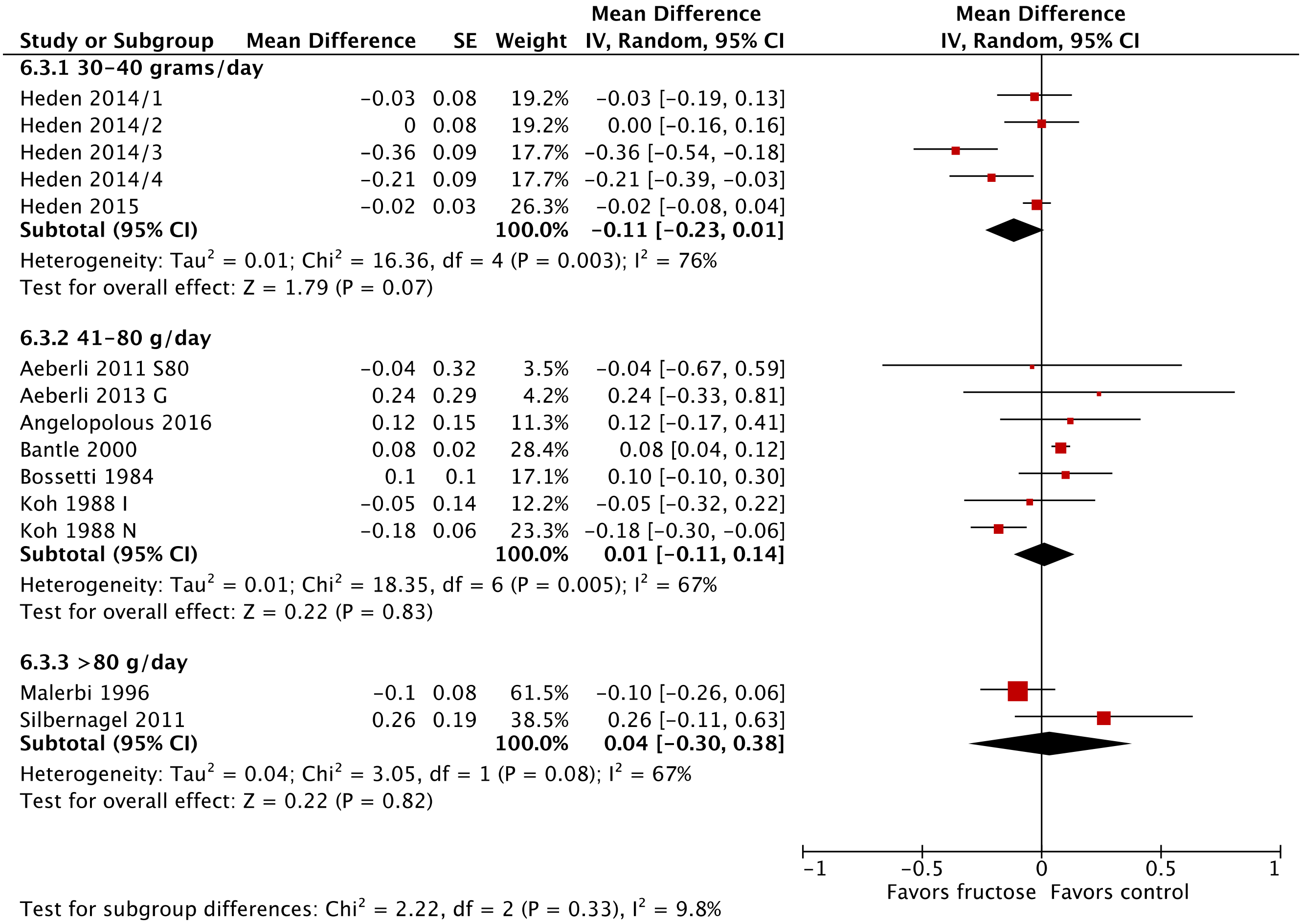

Test for subgroup differences: Chi² = 2.22, df = 2 (P = 0.33), I² = 9.8%

Supplementary figure 14: Subgroup meta-analysis of fasting blood total cholesterol following isoenergetic substitution of glucose or sucrose by fructose in food or beverages by dose of sugar. Values are mean differences [95% CIs] (expressed as mmol/L) between fasting blood total cholesterol after fructose consumption and fasting blood total cholesterol following glucose or sucrose consumption. IV, inverse variance; SE, standard error; G40, fructose/glucose 40 g/day; G80, fructose/glucose 80 g/day; G, glucose; S, sucrose; I, impaired glucose tolerance, N, normal glucose tolerance/body weight; O, overweight

Study or Subgroup	Mean Difference	SE	Weight	Mean Difference IV, Random, 95% CI
**6.4.1 No diabetes**				
Aeberli 2011 S80	−0.04	0.32	1.7%	−0.04 [−0.67, 0.59]
Aeberli 2013 G	0.24	0.29	2.0%	0.24 [−0.33, 0.81]
Angelopolous 2016	0.12	0.15	5.5%	0.12 [−0.17, 0.41]
Bantle 2000	0.08	0.02	14.5%	0.08 [0.04, 0.12]
Bossetti 1984	0.1	0.1	8.4%	0.10 [−0.10, 0.30]
Heden 2014/1	−0.03	0.08	10.0%	−0.03 [−0.19, 0.13]
Heden 2014/2	0	0.08	10.0%	0.00 [−0.16, 0.16]
Heden 2014/3	−0.36	0.09	9.2%	−0.36 [−0.54, −0.18]
Heden 2014/4	−0.21	0.09	9.2%	−0.21 [−0.39, −0.03]
Heden 2015	−0.02	0.03	13.9%	−0.02 [−0.08, 0.04]
Koh 1988 N	−0.18	0.06	11.7%	−0.18 [−0.30, −0.06]
Silbernagel 2011	0.26	0.19	4.0%	0.26 [−0.11, 0.63]
**Subtotal (95% CI)**			**100.0%**	**−0.04 [−0.12, 0.05]**
Heterogeneity: $Tau^2 = 0.01$; $Chi^2 = 49.94$, df = 11 (P < 0.00001); $I^2 = 78\%$				
Test for overall effect: Z = 0.87 (P = 0.39)				
**6.4.2 Impaired glucose tolerance**				
Koh 1988 I	−0.05	0.14	100.0%	−0.05 [−0.32, 0.22]
**Subtotal (95% CI)**			**100.0%**	**−0.05 [−0.32, 0.22]**
Heterogeneity: Not applicable				
Test for overall effect: Z = 0.36 (P = 0.72)				
**6.4.3 Type 2 diabetes**				
Malerbi 1996	−0.1	0.08	100.0%	−0.10 [−0.26, 0.06]
**Subtotal (95% CI)**			**100.0%**	**−0.10 [−0.26, 0.06]**
Heterogeneity: Not applicable				
Test for overall effect: Z = 1.25 (P = 0.21)				

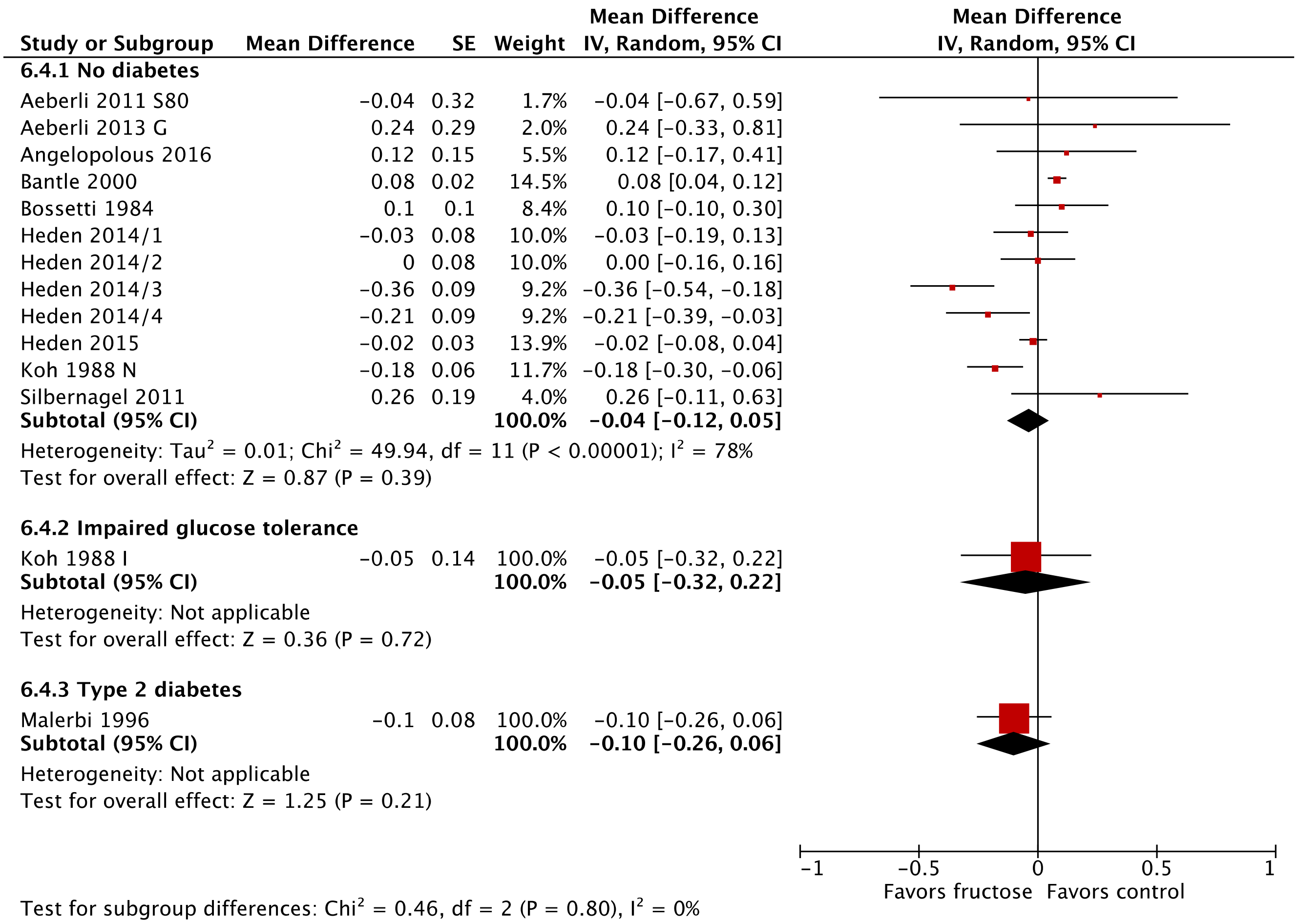

Test for subgroup differences: $Chi^2 = 0.46$, df = 2 (P = 0.80), $I^2 = 0\%$

Supplementary figure 15: Subgroup meta-analysis of fasting blood total cholesterol following isoenergetic substitution of glucose or sucrose by fructose in food or beverages by diabetes status. Values are mean differences [95% CIs] (expressed as mmol/L) between fasting blood total cholesterol after fructose consumption and fasting blood total cholesterol following glucose or sucrose consumption. IV, inverse variance; SE, standard error; G40, fructose/glucose 40 g/day; G80, fructose/glucose 80 g/day; G, glucose; S, sucrose; I, impaired glucose tolerance, N, normal glucose tolerance/body weight; O, overweight

Study or Subgroup	Mean Difference	SE	Weight	Mean Difference IV, Random, 95% CI
**8.4.1 No diabetes**				
Aeberli 2013 G	0.17	0.24	2.2%	0.17 [−0.30, 0.64]
Aeberli 2013 S	−0.06	0.29	1.5%	−0.06 [−0.63, 0.51]
Angelopolous 2016	0.11	0.13	6.9%	0.11 [−0.14, 0.36]
Bantle 2000	−0.02	0.02	48.5%	−0.02 [−0.06, 0.02]
Bossetti 1984	0.13	0.08	15.1%	0.13 [−0.03, 0.29]
Koh 1988 N	−0.11	0.06	22.3%	−0.11 [−0.23, 0.01]
Silbernagel 2011	0.16	0.19	3.4%	0.16 [−0.21, 0.53]
**Subtotal (95% CI)**			**100.0%**	**0.00 [−0.07, 0.07]**
Heterogeneity: $Tau^2 = 0.00$; $Chi^2 = 8.25$, df = 6 (P = 0.22); $I^2 = 27\%$				
Test for overall effect: Z = 0.04 (P = 0.97)				
**8.4.2 Impaired glucose tolerance**				
Koh 1988 I	−0.06	0.13	100.0%	−0.06 [−0.31, 0.19]
**Subtotal (95% CI)**			**100.0%**	**−0.06 [−0.31, 0.19]**
Heterogeneity: Not applicable				
Test for overall effect: Z = 0.46 (P = 0.64)				
**8.4.3 Type 2 diabetes**				
Malerbi 1996	−0.2	0.08	100.0%	−0.20 [−0.36, −0.04]
**Subtotal (95% CI)**			**100.0%**	**−0.20 [−0.36, −0.04]**
Heterogeneity: Not applicable				
Test for overall effect: Z = 2.50 (P = 0.01)				

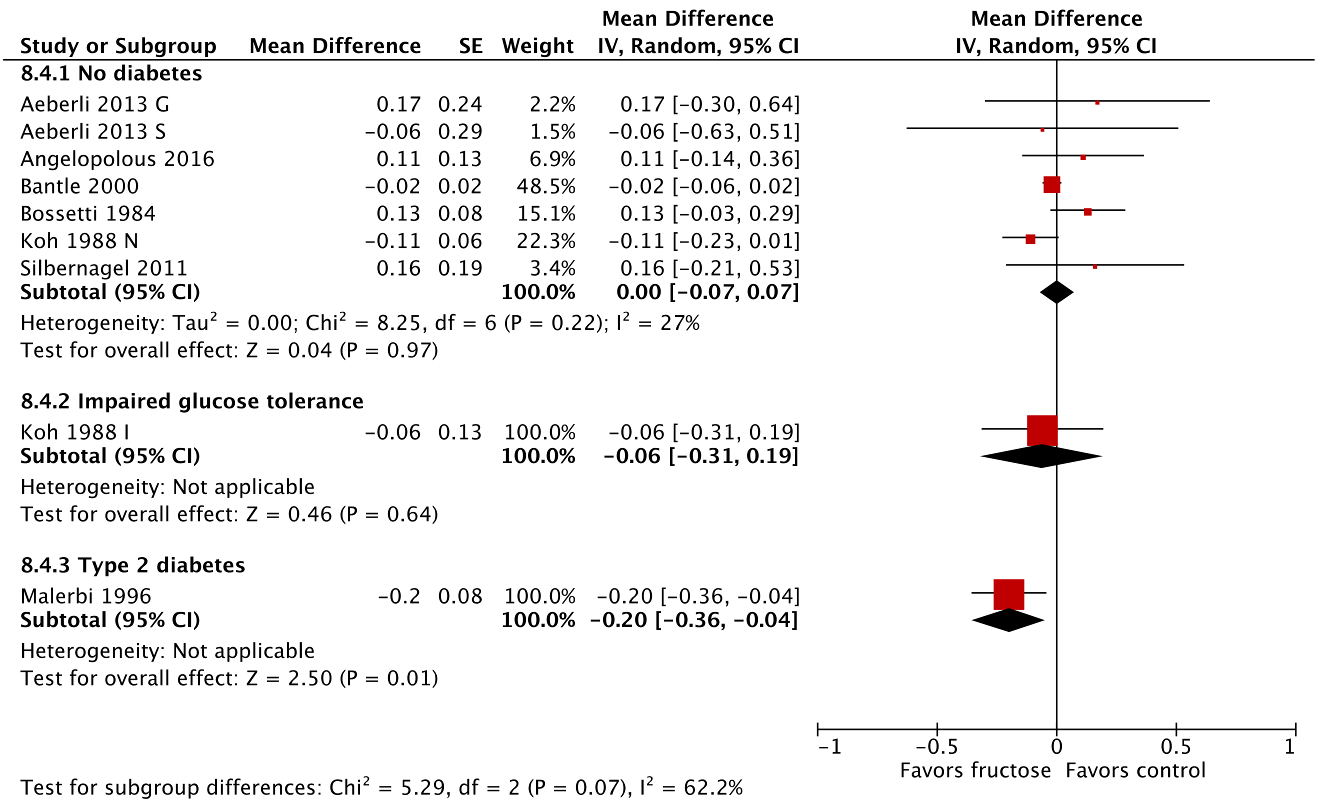

Test for subgroup differences: $Chi^2 = 5.29$, df = 2 (P = 0.07), $I^2 = 62.2\%$

Supplementary figure 16: Subgroup meta-analysis of fasting LDL cholesterol following isoenergetic substitution of glucose or sucrose by fructose in food or beverages by diabetes status. Values are mean differences [95% CIs] (expressed as mmol/L) between fasting blood LDL cholesterol after fructose consumption and fasting blood LDL cholesterol following glucose or sucrose consumption. IV, inverse variance; SE, standard error; G40, fructose/glucose 40 g/day; G80, fructose/glucose 80 g/day; G, glucose; S, sucrose; I, impaired glucose tolerance, N, normal glucose tolerance/body weight; O, overweight

Study or Subgroup	Mean Difference	SE	Weight	Mean Difference IV, Random, 95% CI
**8.2.1 BMI</= 25**				
Aeberli 2013 G	0.17	0.24	10.8%	0.17 [−0.30, 0.64]
Aeberli 2013 S	−0.06	0.29	7.8%	−0.06 [−0.63, 0.51]
Bossetti 1984	0.13	0.08	37.7%	0.13 [−0.03, 0.29]
Koh 1988 N	−0.11	0.06	43.7%	−0.11 [−0.23, 0.01]
**Subtotal (95% CI)**			**100.0%**	**0.01 [−0.16, 0.19]**
Heterogeneity: Tau^2 = 0.01; Chi^2 = 6.41, df = 3 (P = 0.09); I^2 = 53%				
Test for overall effect: Z = 0.17 (P = 0.87)				
**8.2.2 BMI 25.1−29.9**				
Angelopolous 2016	0.11	0.13	12.0%	0.11 [−0.14, 0.36]
Bantle 2000	−0.02	0.02	46.9%	−0.02 [−0.06, 0.02]
Koh 1988 I	−0.06	0.13	12.0%	−0.06 [−0.31, 0.19]
Malerbi 1996	−0.2	0.08	22.7%	−0.20 [−0.36, −0.04]
Silbernagel 2011	0.16	0.19	6.4%	0.16 [−0.21, 0.53]
**Subtotal (95% CI)**			**100.0%**	**−0.04 [−0.14, 0.06]**
Heterogeneity: Tau^2 = 0.01; Chi^2 = 6.94, df = 4 (P = 0.14); I^2 = 42%				
Test for overall effect: Z = 0.75 (P = 0.45)				
**8.2.3 30 and greater**				
**Subtotal (95% CI)**				**Not estimable**
Heterogeneity: Not applicable				
Test for overall effect: Not applicable				

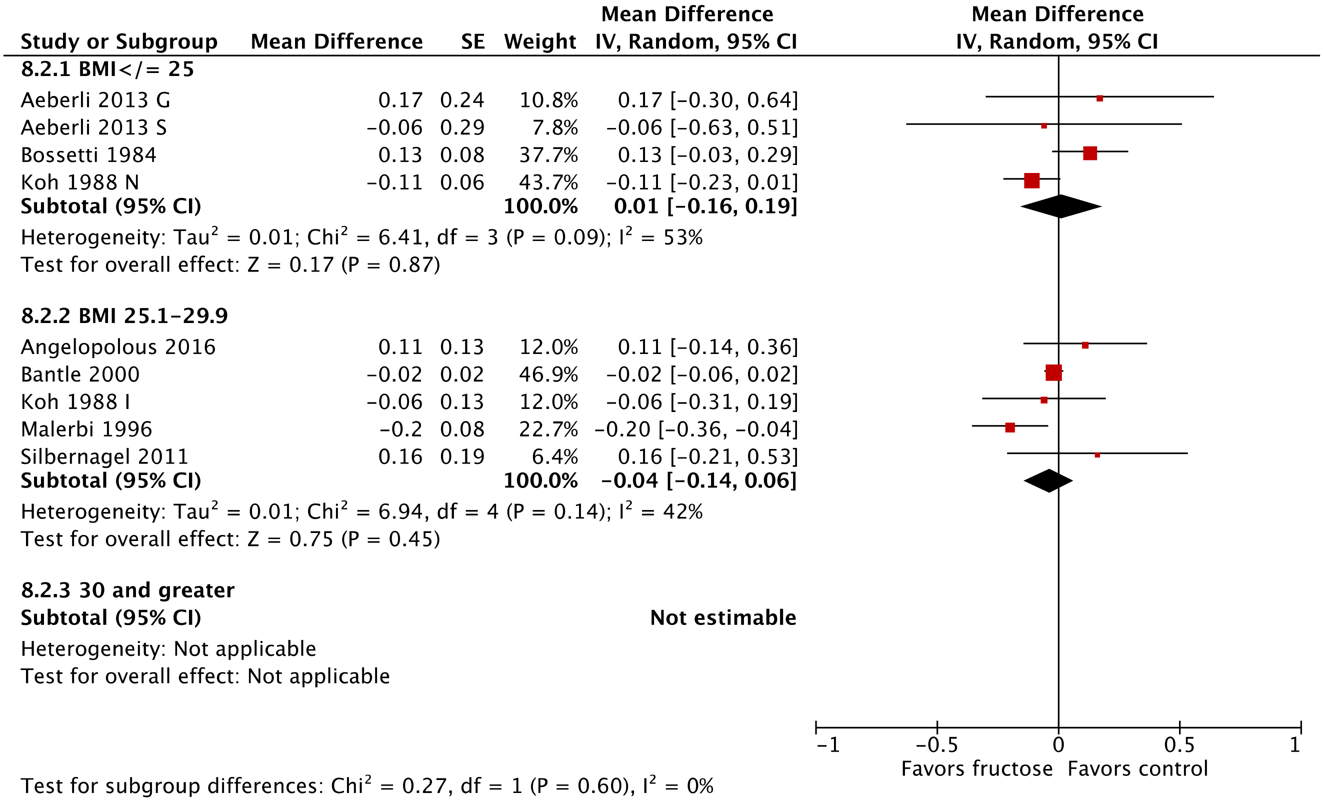

Test for subgroup differences: Chi^2 = 0.27, df = 1 (P = 0.60), I^2 = 0%

Supplementary figure 17: Subgroup meta-analysis of fasting LDL cholesterol following isoenergetic substitution of glucose or sucrose by fructose in food or beverages by baseline BMI. Values are mean differences [95% CIs] (expressed as mmol/L) between fasting blood LDL cholesterol after fructose consumption and fasting blood LDL cholesterol following glucose or sucrose consumption. IV, inverse variance; SE, standard error; G40, fructose/glucose 40 g/day; G80, fructose/glucose 80 g/day; G, glucose; S, sucrose; I, impaired glucose tolerance, N, normal glucose tolerance/body weight; O, overweight

Study or Subgroup	Mean Difference	SE	Weight	Mean Difference IV, Random, 95% CI
**8.3.1 30-40 grams/day**				
**Subtotal (95% CI)**				**Not estimable**
Heterogeneity: Not applicable				
Test for overall effect: Not applicable				
**8.3.2 41-80 g/day**				
Aeberli 2013 G	0.17	0.24	1.7%	0.17 [-0.30, 0.64]
Aeberli 2013 S	-0.06	0.29	1.2%	-0.06 [-0.63, 0.51]
Angelopolous 2016	0.11	0.13	5.5%	0.11 [-0.14, 0.36]
Bantle 2000	-0.02	0.02	53.7%	-0.02 [-0.06, 0.02]
Bossetti 1984	0.13	0.08	12.7%	0.13 [-0.03, 0.29]
Koh 1988 I	-0.06	0.13	5.5%	-0.06 [-0.31, 0.19]
Koh 1988 N	-0.11	0.06	19.8%	-0.11 [-0.23, 0.01]
**Subtotal (95% CI)**			**100.0%**	**-0.01 [-0.07, 0.05]**
Heterogeneity: $Tau^2 = 0.00$; $Chi^2 = 7.49$, df = 6 (P = 0.28); $I^2 = 20\%$				
Test for overall effect: Z = 0.35 (P = 0.73)				
**8.3.3 >80 g/day**				
Malerbi 1996	-0.2	0.08	61.5%	-0.20 [-0.36, -0.04]
Silbernagel 2011	0.16	0.19	38.5%	0.16 [-0.21, 0.53]
**Subtotal (95% CI)**			**100.0%**	**-0.06 [-0.40, 0.28]**
Heterogeneity: $Tau^2 = 0.04$; $Chi^2 = 3.05$, df = 1 (P = 0.08); $I^2 = 67\%$				
Test for overall effect: Z = 0.35 (P = 0.73)				

Test for subgroup differences: $Chi^2 = 0.08$, df = 1 (P = 0.78), $I^2 = 0\%$

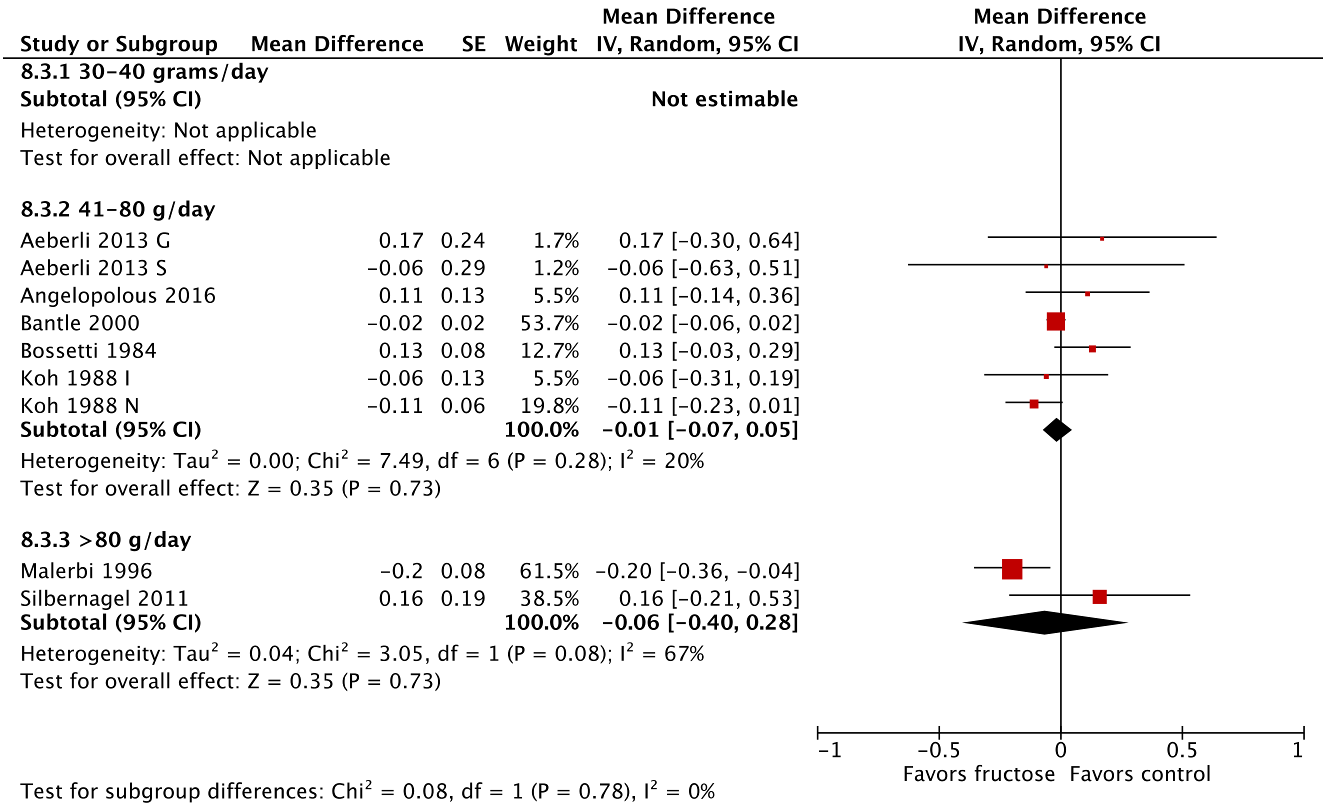

Supplementary figure 18: Subgroup meta-analysis of fasting LDL cholesterol following isoenergetic substitution of glucose or sucrose by fructose in food or beverages by dose of sugar. Values are mean differences [95% CIs] (expressed as mmol/L) between fasting blood LDL cholesterol after fructose consumption and fasting blood LDL cholesterol following glucose or sucrose consumption. IV, inverse variance; SE, standard error; G40, fructose/glucose 40 g/day; G80, fructose/glucose 80 g/day; G, glucose; S, sucrose; I, impaired glucose tolerance, N, normal glucose tolerance/body weight; O, overweight

Study or Subgroup	Mean Difference	SE	Weight	Mean Difference IV, Random, 95% CI
**9.2.1 BMI</= 25**				
Aeberli 2013 G	0.05	0.12	3.1%	0.05 [−0.19, 0.29]
Aeberli 2013 S	−0.01	0.12	3.1%	−0.01 [−0.25, 0.23]
Bossetti 1984	0.03	0.05	17.7%	0.03 [−0.07, 0.13]
Heden 2014/1	0	0.03	49.3%	0.00 [−0.06, 0.06]
Heden 2014/3	−0.11	0.07	9.1%	−0.11 [−0.25, 0.03]
Koh 1988 N	−0.08	0.05	17.7%	−0.08 [−0.18, 0.02]
**Subtotal (95% CI)**			**100.0%**	**−0.02 [−0.06, 0.02]**
Heterogeneity: $Tau^2 = 0.00$; $Chi^2 = 4.87$, df = 5 (P = 0.43); $I^2 = 0\%$				
Test for overall effect: Z = 0.84 (P = 0.40)				
**9.2.2 BMI 25.1-29.9**				
Angelopolous 2016	−0.03	0.05	3.3%	−0.03 [−0.13, 0.07]
Bantle 2000	0	0.01	81.5%	0.00 [−0.02, 0.02]
Koh 1988 I	0.02	0.04	5.1%	0.02 [−0.06, 0.10]
Malerbi 1996	0	0.04	5.1%	0.00 [−0.08, 0.08]
Silbernagel 2011	0.03	0.04	5.1%	0.03 [−0.05, 0.11]
**Subtotal (95% CI)**			**100.0%**	**0.00 [−0.02, 0.02]**
Heterogeneity: $Tau^2 = 0.00$; $Chi^2 = 1.14$, df = 4 (P = 0.89); $I^2 = 0\%$				
Test for overall effect: Z = 0.17 (P = 0.86)				
**9.2.3 30 and greater**				
Heden 2014/2	0	0.02	66.7%	0.00 [−0.04, 0.04]
Heden 2014/4	0.06	0.04	33.3%	0.06 [−0.02, 0.14]
**Subtotal (95% CI)**			**100.0%**	**0.02 [−0.04, 0.08]**
Heterogeneity: $Tau^2 = 0.00$; $Chi^2 = 1.80$, df = 1 (P = 0.18); $I^2 = 44\%$				
Test for overall effect: Z = 0.71 (P = 0.48)				

Test for subgroup differences: $Chi^2 = 1.23$, df = 2 (P = 0.54), $I^2 = 0\%$

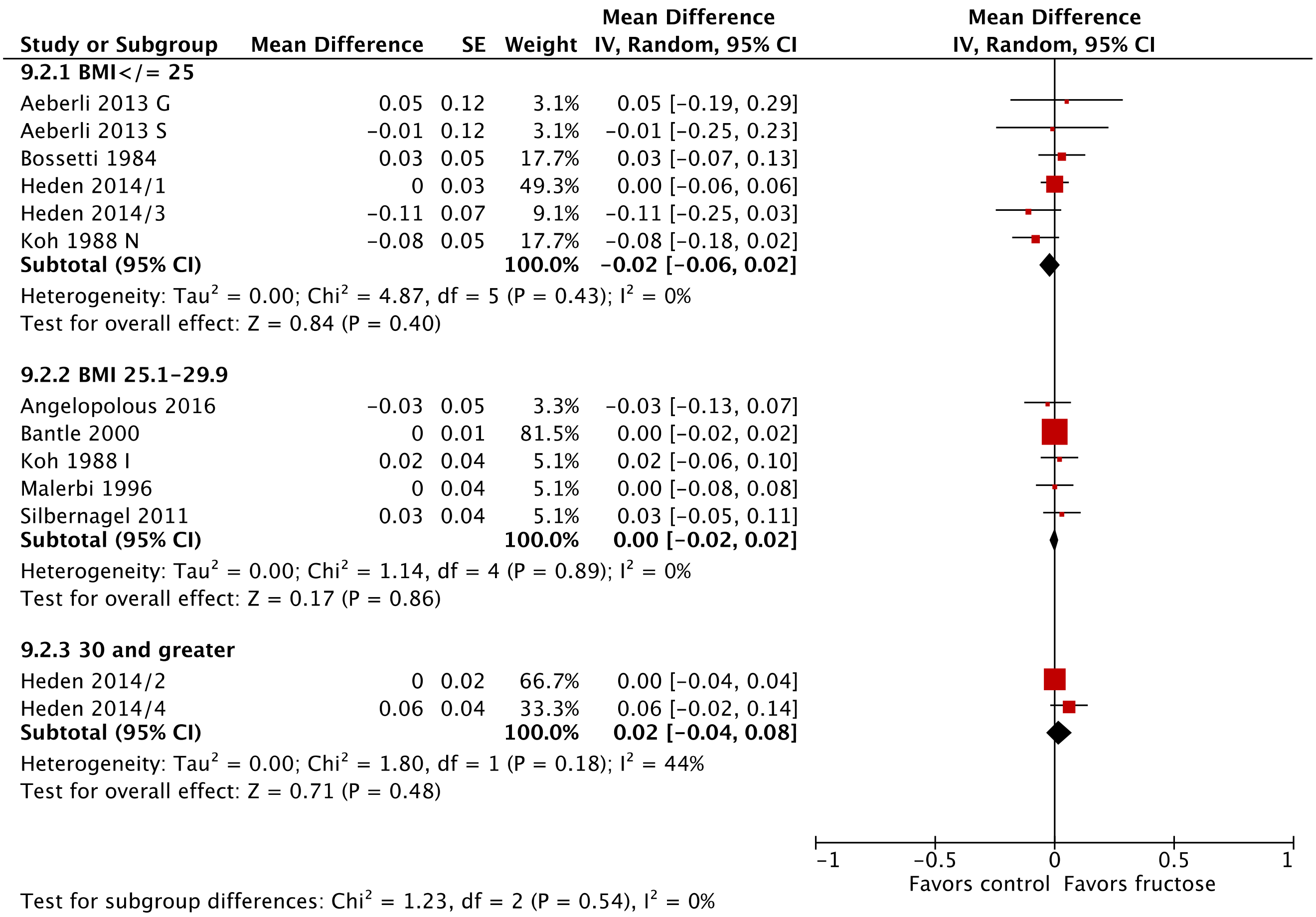

Supplementary figure 19: Subgroup meta-analysis of fasting HDL cholesterol following isoenergetic substitution of glucose or sucrose by fructose in food or beverages by baseline BMI. Values are mean differences [95% CIs] (expressed as mmol/L) between fasting blood HDL cholesterol after fructose consumption and fasting blood HDL cholesterol following glucose or sucrose consumption. IV, inverse variance; SE, standard error; G40, fructose/glucose 40 g/day; G80, fructose/glucose 80 g/day; G, glucose; S, sucrose; I, impaired glucose tolerance, N, normal glucose tolerance/body weight; O, overweight

Study or Subgroup	Mean Difference	SE	Weight	Mean Difference IV, Random, 95% CI
**9.3.1 30-40 grams/day**				
Heden 2014/1	0	0.03	29.0%	0.00 [-0.06, 0.06]
Heden 2014/2	0	0.02	43.1%	0.00 [-0.04, 0.04]
Heden 2014/3	-0.11	0.07	8.0%	-0.11 [-0.25, 0.03]
Heden 2014/4	0.06	0.04	19.9%	0.06 [-0.02, 0.14]
**Subtotal (95% CI)**			**100.0%**	**0.00 [-0.04, 0.04]**
Heterogeneity: $Tau^2 = 0.00$; $Chi^2 = 4.67$, df = 3 (P = 0.20); $I^2 = 36\%$				
Test for overall effect: Z = 0.15 (P = 0.88)				
**9.3.2 41-80 g/day**				
Aeberli 2013 G	0.05	0.12	0.6%	0.05 [-0.19, 0.29]
Aeberli 2013 S	-0.01	0.12	0.6%	-0.01 [-0.25, 0.23]
Angelopolous 2016	-0.03	0.05	3.3%	-0.03 [-0.13, 0.07]
Bantle 2000	0	0.01	83.6%	0.00 [-0.02, 0.02]
Bossetti 1984	0.03	0.05	3.3%	0.03 [-0.07, 0.13]
Koh 1988 I	0.02	0.04	5.2%	0.02 [-0.06, 0.10]
Koh 1988 N	-0.08	0.05	3.3%	-0.08 [-0.18, 0.02]
**Subtotal (95% CI)**			**100.0%**	**-0.00 [-0.02, 0.02]**
Heterogeneity: $Tau^2 = 0.00$; $Chi^2 = 3.69$, df = 6 (P = 0.72); $I^2 = 0\%$				
Test for overall effect: Z = 0.15 (P = 0.88)				
**9.3.3 >80 g/day**				
Malerbi 1996	0	0.04	50.0%	0.00 [-0.08, 0.08]
Silbernagel 2011	0.03	0.04	50.0%	0.03 [-0.05, 0.11]
**Subtotal (95% CI)**			**100.0%**	**0.01 [-0.04, 0.07]**
Heterogeneity: $Tau^2 = 0.00$; $Chi^2 = 0.28$, df = 1 (P = 0.60); $I^2 = 0\%$				
Test for overall effect: Z = 0.53 (P = 0.60)				

Test for subgroup differences: $Chi^2 = 0.32$, df = 2 (P = 0.85), $I^2 = 0\%$

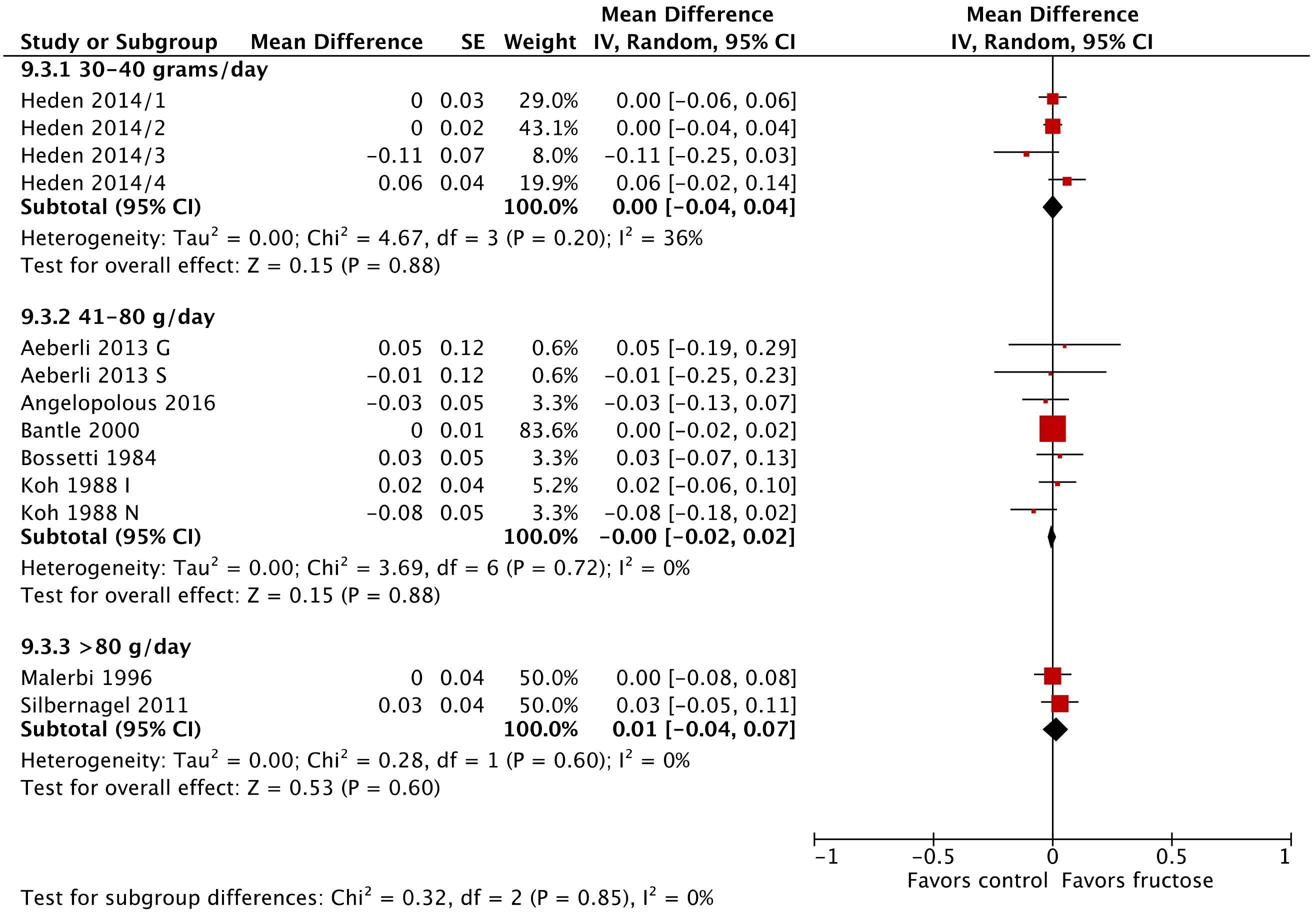

Supplementary figure 20: Subgroup meta-analysis of fasting HDL cholesterol following isoenergetic substitution of glucose or sucrose by fructose in food or beverages by dose of sugar. Values are mean differences [95% CIs] (expressed as mmol/L) between fasting blood HDL cholesterol after fructose consumption and fasting blood HDL cholesterol following glucose or sucrose consumption. IV, inverse variance; SE, standard error; G40, fructose/glucose 40 g/day; G80, fructose/glucose 80 g/day; G, glucose; S, sucrose; I, impaired glucose tolerance, N, normal glucose tolerance/body weight; O, overweight

Study or Subgroup	Mean Difference	SE	Weight	Mean Difference IV, Random, 95% CI
**9.4.1 No diabetes**				
Aeberli 2013 G	0.05	0.12	0.4%	0.05 [−0.19, 0.29]
Aeberli 2013 S	−0.01	0.12	0.4%	−0.01 [−0.25, 0.23]
Angelopolous 2016	−0.03	0.05	2.4%	−0.03 [−0.13, 0.07]
Bantle 2000	0	0.01	61.0%	0.00 [−0.02, 0.02]
Bossetti 1984	0.03	0.05	2.4%	0.03 [−0.07, 0.13]
Heden 2014/1	0	0.03	6.8%	0.00 [−0.06, 0.06]
Heden 2014/2	0	0.02	15.2%	0.00 [−0.04, 0.04]
Heden 2014/3	−0.11	0.07	1.2%	−0.11 [−0.25, 0.03]
Heden 2014/4	0.06	0.04	3.8%	0.06 [−0.02, 0.14]
Koh 1988 N	−0.08	0.05	2.4%	−0.08 [−0.18, 0.02]
Silbernagel 2011	0.03	0.04	3.8%	0.03 [−0.05, 0.11]
**Subtotal (95% CI)**			**100.0%**	**0.00 [−0.02, 0.02]**
Heterogeneity: $Tau^2 = 0.00$; $Chi^2 = 8.74$, df = 10 (P = 0.56); $I^2 = 0\%$				
Test for overall effect: Z = 0.04 (P = 0.97)				
**9.4.2 Impaired glucose tolerance**				
Koh 1988 I	0.02	0.04	100.0%	0.02 [−0.06, 0.10]
**Subtotal (95% CI)**			**100.0%**	**0.02 [−0.06, 0.10]**
Heterogeneity: Not applicable				
Test for overall effect: Z = 0.50 (P = 0.62)				
**9.4.3 Type 2 diabetes**				
Malerbi 1996	0	0.04	100.0%	0.00 [−0.08, 0.08]
**Subtotal (95% CI)**			**100.0%**	**0.00 [−0.08, 0.08]**
Heterogeneity: Not applicable				
Test for overall effect: Z = 0.00 (P = 1.00)				

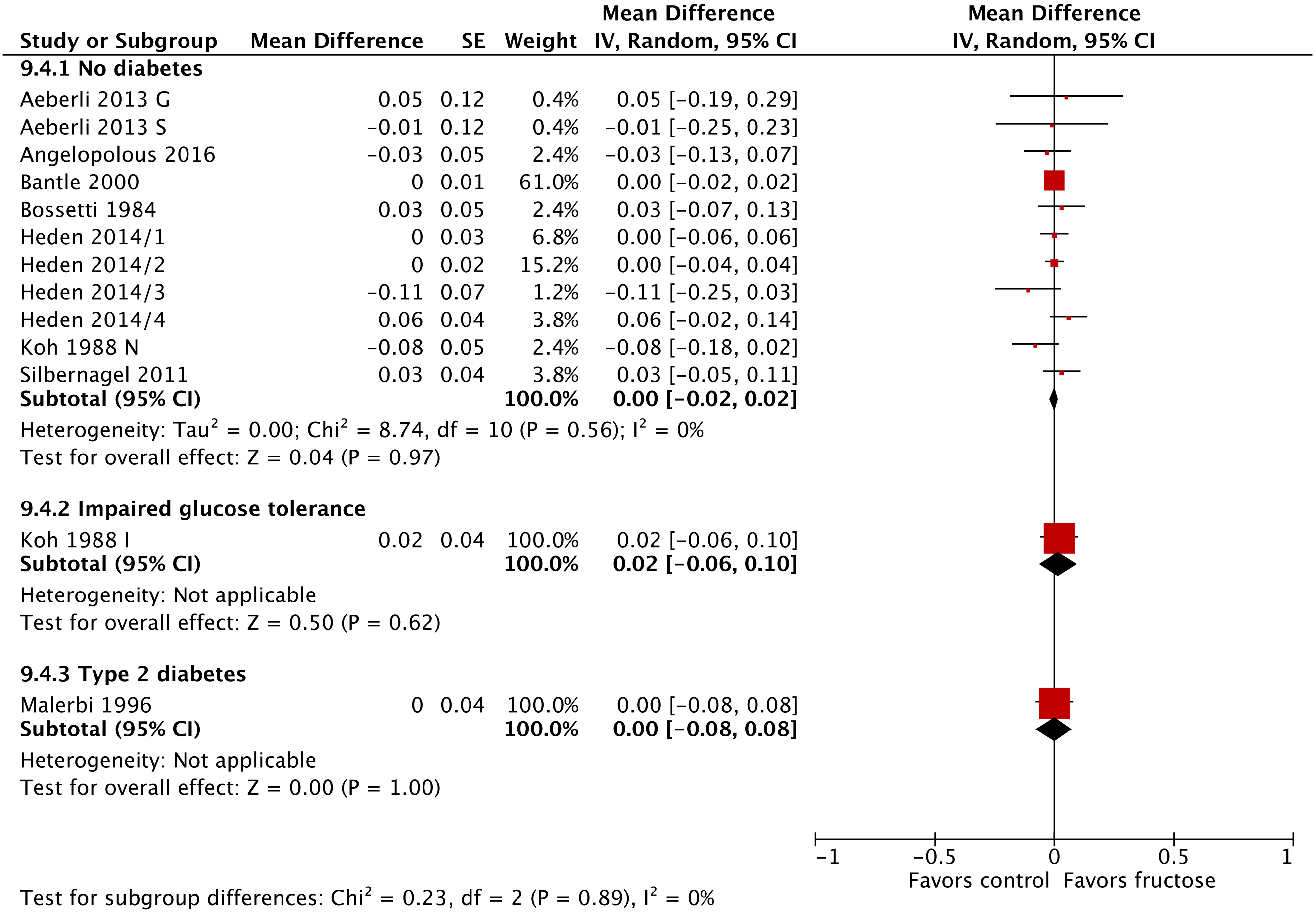

Test for subgroup differences: $Chi^2 = 0.23$, df = 2 (P = 0.89), $I^2 = 0\%$

Supplementary figure 21: Subgroup meta-analysis of fasting HDL cholesterol following isoenergetic substitution of glucose or sucrose by fructose in food or beverages by diabetes status. Values are mean differences [95% CIs] (expressed as mmol/L) between fasting blood HDL cholesterol after fructose consumption and fasting blood HDL cholesterol following glucose or sucrose consumption. IV, inverse variance; SE, standard error; G40, fructose/glucose 40 g/day; G80, fructose/glucose 80 g/day; G, glucose; S, sucrose; I, impaired glucose tolerance, N, normal glucose tolerance/body weight; O, overweight

Study or Subgroup	Mean Difference	SE	Weight	Mean Difference IV, Random, 95% CI
**7.2.1 BMI</= 25**				
Aeberli 2013 G	0.02	0.17	3.6%	0.02 [−0.31, 0.35]
Aeberli 2013 S	0.08	0.18	3.2%	0.08 [−0.27, 0.43]
Bossetti 1984	−0.07	0.05	24.5%	−0.07 [−0.17, 0.03]
Heden 2014/1	0.09	0.05	24.5%	0.09 [−0.01, 0.19]
Heden 2014/3	−0.08	0.06	19.7%	−0.08 [−0.20, 0.04]
Koh 1988 N	0.02	0.05	24.5%	0.02 [−0.08, 0.12]
**Subtotal (95% CI)**			**100.0%**	**−0.00 [−0.07, 0.06]**
Heterogeneity: Tau² = 0.00; Chi² = 7.34, df = 5 (P = 0.20); I² = 32%				
Test for overall effect: Z = 0.08 (P = 0.94)				
**7.2.2 BMI 25.1-29.9**				
Angelopolous 2016	0.08	0.1	15.7%	0.08 [−0.12, 0.28]
Bantle 2000 F	−0.04	0.03	18.9%	−0.04 [−0.10, 0.02]
Bantle 2000 M	0.3	0.03	18.9%	0.30 [0.24, 0.36]
Koh 1988 I	−0.21	0.09	16.3%	−0.21 [−0.39, −0.03]
Malerbi 1996	−0.1	0.04	18.6%	−0.10 [−0.18, −0.02]
Silbernagel 2011	0.19	0.17	11.6%	0.19 [−0.14, 0.52]
**Subtotal (95% CI)**			**100.0%**	**0.03 [−0.15, 0.21]**
Heterogeneity: Tau² = 0.04; Chi² = 100.47, df = 5 (P < 0.00001); I² = 95%				
Test for overall effect: Z = 0.34 (P = 0.74)				
**7.2.3 30 and greater**				
Heden 2014/2	0.04	0.05	48.0%	0.04 [−0.06, 0.14]
Heden 2014/4	0.06	0.06	44.2%	0.06 [−0.06, 0.18]
Jin 2014	−0.57	0.26	7.9%	−0.57 [−1.08, −0.06]
**Subtotal (95% CI)**			**100.0%**	**0.00 [−0.15, 0.15]**
Heterogeneity: Tau² = 0.01; Chi² = 5.60, df = 2 (P = 0.06); I² = 64%				
Test for overall effect: Z = 0.01 (P = 0.99)				

Test for subgroup differences: Chi² = 0.12, df = 2 (P = 0.94), I² = 0%

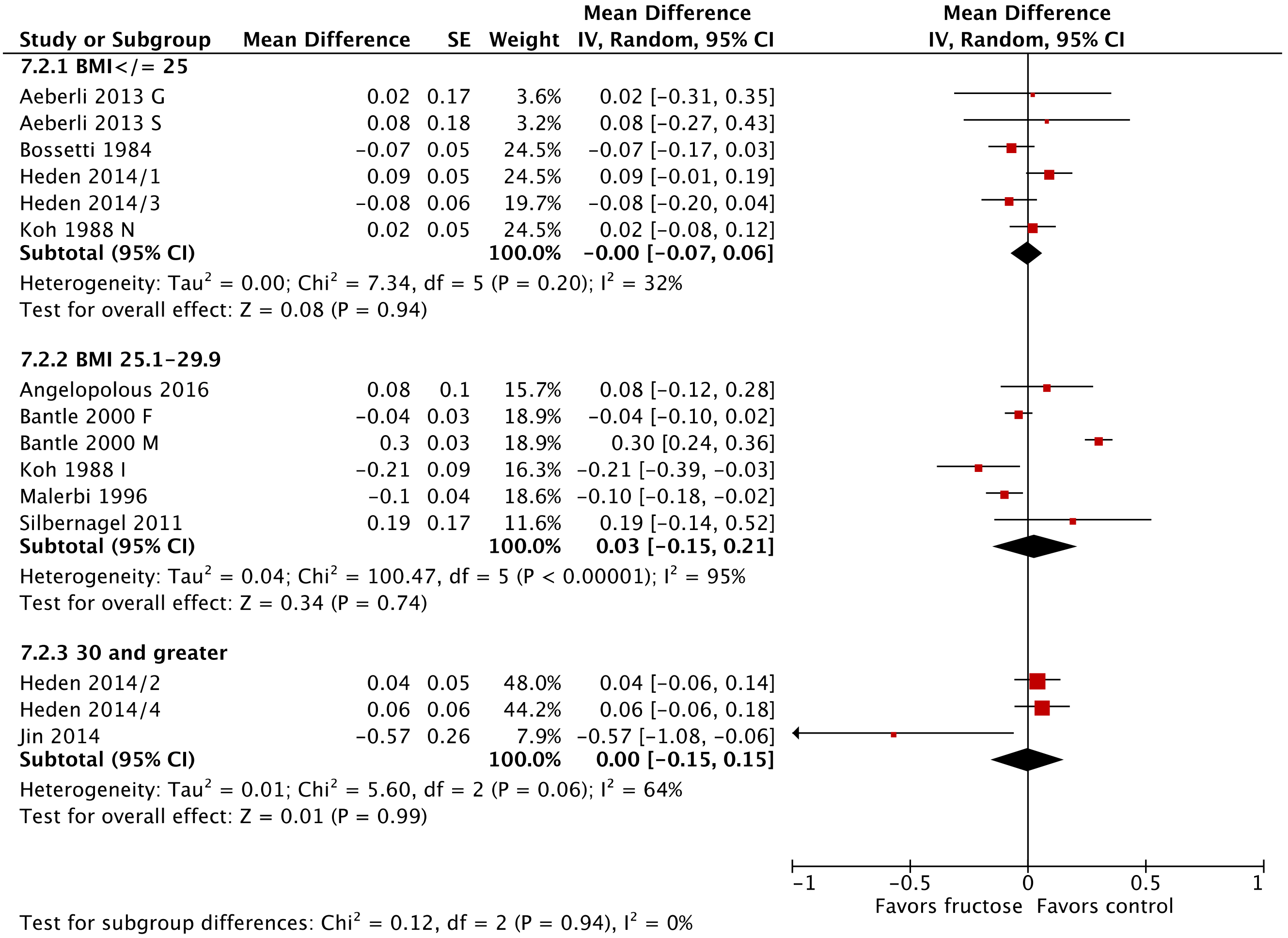

Supplementary figure 22: Subgroup meta-analysis of fasting blood triglycerides following isoenergetic substitution of glucose or sucrose by fructose in food or beverages by baseline BMI. Values are mean differences [95% CIs] (expressed as mmol/L) between fasting blood triglycerides after fructose consumption and fasting blood triglycerides following glucose or sucrose consumption. IV, inverse variance; SE, standard error; G40, fructose/glucose 40 g/day; G80, fructose/glucose 80 g/day; G, glucose; S, sucrose; I, impaired glucose tolerance, N, normal glucose tolerance/body weight; O, overweight

Study or Subgroup	Mean Difference	SE	Weight	Mean Difference IV, Random, 95% CI
**7.3.1 30–40 grams/day**				
Heden 2014/1	0.09	0.05	27.7%	0.09 [−0.01, 0.19]
Heden 2014/2	0.04	0.05	27.7%	0.04 [−0.06, 0.14]
Heden 2014/3	−0.08	0.06	22.3%	−0.08 [−0.20, 0.04]
Heden 2014/4	0.06	0.06	22.3%	0.06 [−0.06, 0.18]
**Subtotal (95% CI)**			**100.0%**	**0.03 [−0.04, 0.10]**
Heterogeneity: $Tau^2 = 0.00$; $Chi^2 = 5.07$, df = 3 (P = 0.17); $I^2 = 41\%$				
Test for overall effect: Z = 0.89 (P = 0.37)				
**7.3.2 41–80 g/day**				
Aeberli 2013 G	0.02	0.17	8.4%	0.02 [−0.31, 0.35]
Aeberli 2013 S	0.08	0.18	7.9%	0.08 [−0.27, 0.43]
Angelopolous 2016	0.08	0.1	12.0%	0.08 [−0.12, 0.28]
Bantle 2000 F	−0.04	0.03	15.1%	−0.04 [−0.10, 0.02]
Bantle 2000 M	0.3	0.03	15.1%	0.30 [0.24, 0.36]
Bossetti 1984	−0.07	0.05	14.5%	−0.07 [−0.17, 0.03]
Koh 1988 I	−0.21	0.09	12.5%	−0.21 [−0.39, −0.03]
Koh 1988 N	0.02	0.05	14.5%	0.02 [−0.08, 0.12]
**Subtotal (95% CI)**			**100.0%**	**0.02 [−0.12, 0.17]**
Heterogeneity: $Tau^2 = 0.03$; $Chi^2 = 90.69$, df = 7 (P < 0.00001); $I^2 = 92\%$				
Test for overall effect: Z = 0.32 (P = 0.75)				
**7.3.3 >80 g/day**				
Jin 2014	−0.57	0.26	20.2%	−0.57 [−1.08, −0.06]
Malerbi 1996	−0.1	0.04	49.0%	−0.10 [−0.18, −0.02]
Silbernagel 2011	0.19	0.17	30.8%	0.19 [−0.14, 0.52]
**Subtotal (95% CI)**			**100.0%**	**−0.11 [−0.40, 0.19]**
Heterogeneity: $Tau^2 = 0.04$; $Chi^2 = 6.16$, df = 2 (P = 0.05); $I^2 = 68\%$				
Test for overall effect: Z = 0.70 (P = 0.48)				

Test for subgroup differences: $Chi^2 = 0.78$, df = 2 (P = 0.68), $I^2 = 0\%$

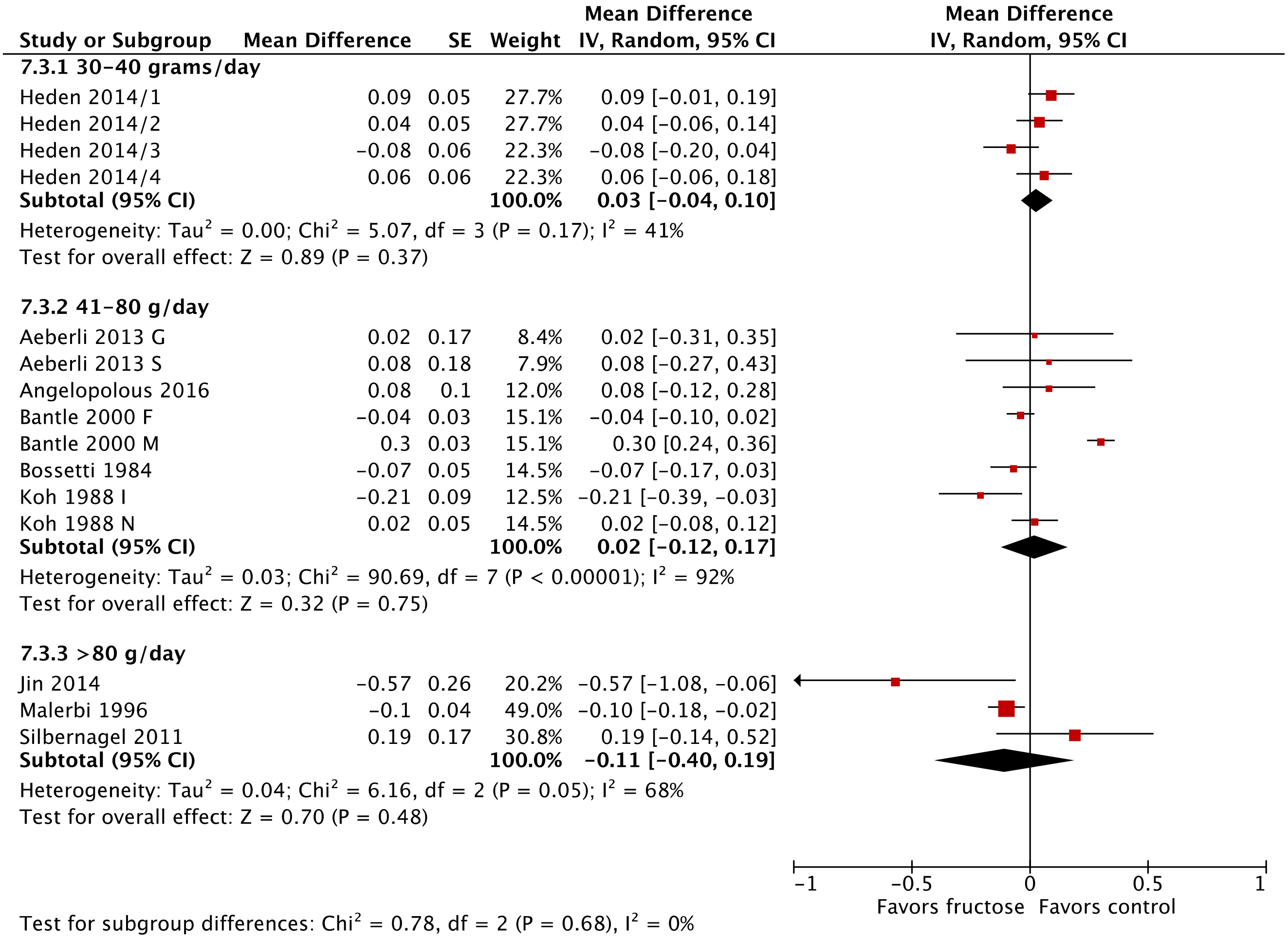

Supplementary figure 23: Subgroup meta-analysis of fasting blood triglycerides following isoenergetic substitution of glucose or sucrose by fructose in food or beverages by dose of sugar. Values are mean differences [95% CIs] (expressed as mmol/L) between fasting blood triglycerides after fructose consumption and fasting blood triglycerides following glucose or sucrose consumption. IV, inverse variance; SE, standard error; G40, fructose/glucose 40 g/day; G80, fructose/glucose 80 g/day; G, glucose; S, sucrose; I, impaired glucose tolerance, N, normal glucose tolerance/body weight; O, overweight

Study or Subgroup	Mean Difference	SE	Weight	Mean Difference IV, Random, 95% CI
**7.4.1 No diabetes**				
Aeberli 2013 G	0.02	0.17	4.5%	0.02 [−0.31, 0.35]
Aeberli 2013 S	0.08	0.18	4.2%	0.08 [−0.27, 0.43]
Angelopolous 2016	0.08	0.1	7.3%	0.08 [−0.12, 0.28]
Bantle 2000 F	−0.04	0.03	10.2%	−0.04 [−0.10, 0.02]
Bantle 2000 M	0.3	0.03	10.2%	0.30 [0.24, 0.36]
Bossetti 1984	−0.07	0.05	9.5%	−0.07 [−0.17, 0.03]
Heden 2014/1	0.09	0.05	9.5%	0.09 [−0.01, 0.19]
Heden 2014/2	0.04	0.05	9.5%	0.04 [−0.06, 0.14]
Heden 2014/3	−0.08	0.06	9.1%	−0.08 [−0.20, 0.04]
Heden 2014/4	0.06	0.06	9.1%	0.06 [−0.06, 0.18]
Jin 2014	−0.57	0.26	2.6%	−0.57 [−1.08, −0.06]
Koh 1988 N	0.02	0.05	9.5%	0.02 [−0.08, 0.12]
Silbernagel 2011	0.19	0.17	4.5%	0.19 [−0.14, 0.52]
**Subtotal (95% CI)**			**100.0%**	**0.04 [−0.06, 0.13]**
Heterogeneity: $Tau^2 = 0.02$; $Chi^2 = 94.51$, df = 12 ($P < 0.00001$); $I^2 = 87\%$				
Test for overall effect: Z = 0.77 (P = 0.44)				
**7.4.2 Impaired glucose tolerance**				
Koh 1988 I	−0.21	0.09	100.0%	−0.21 [−0.39, −0.03]
**Subtotal (95% CI)**			**100.0%**	**−0.21 [−0.39, −0.03]**
Heterogeneity: Not applicable				
Test for overall effect: Z = 2.33 (P = 0.02)				
**7.4.3 Type 2 diabetes**				
Malerbi 1996	−0.1	0.04	100.0%	−0.10 [−0.18, −0.02]
**Subtotal (95% CI)**			**100.0%**	**−0.10 [−0.18, −0.02]**
Heterogeneity: Not applicable				
Test for overall effect: Z = 2.50 (P = 0.01)				

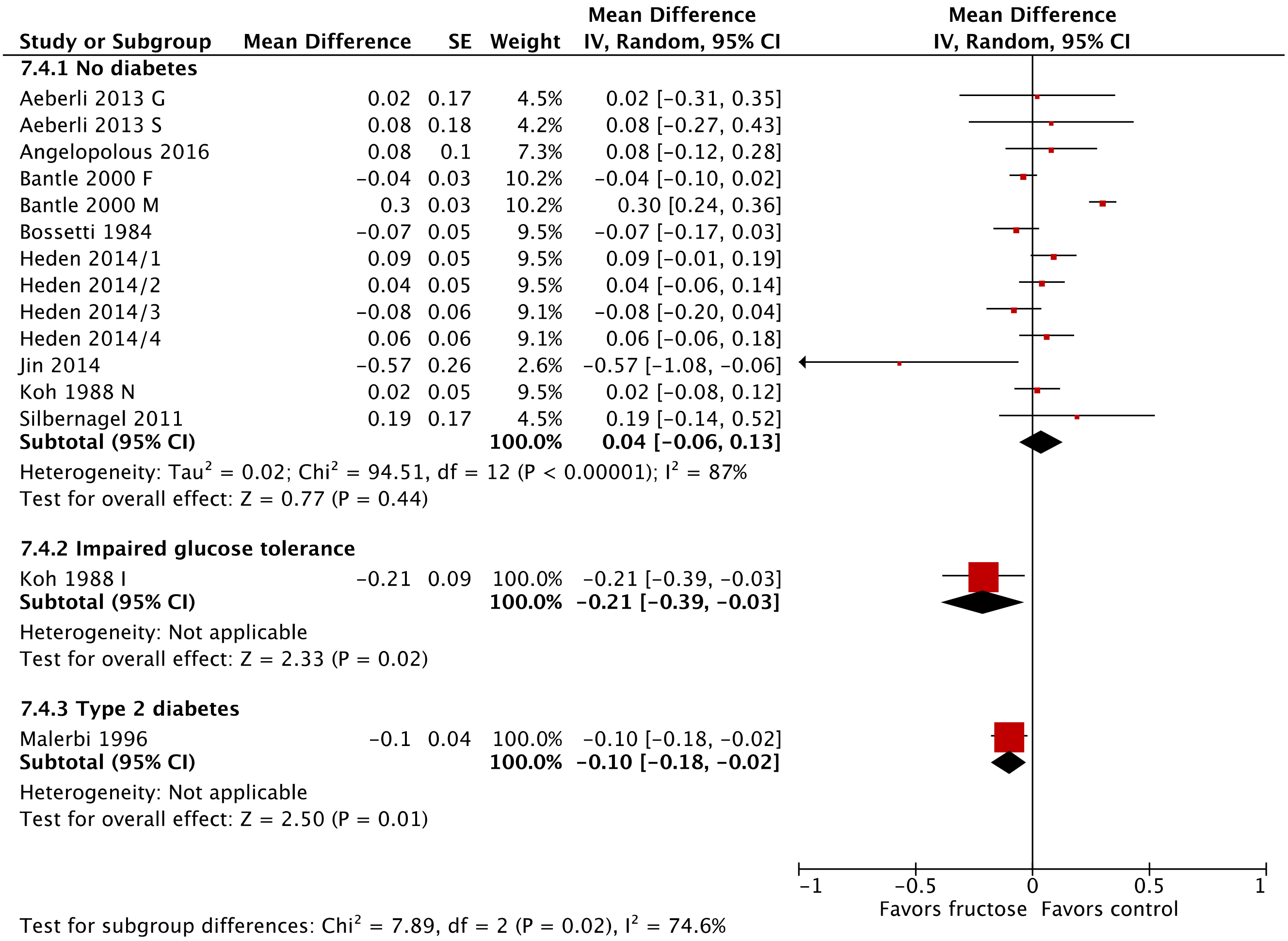

Test for subgroup differences: $Chi^2 = 7.89$, df = 2 (P = 0.02), $I^2 = 74.6\%$

Supplementary figure 24: Subgroup meta-analysis of fasting blood triglycerides following isoenergetic substitution of glucose or sucrose by fructose in food or beverages by diabetes status. Values are mean differences [95% CIs] (expressed as mmol/L) between fasting blood triglycerides after fructose consumption and fasting blood triglycerides following glucose or sucrose consumption. IV, inverse variance; SE, standard error; G40, fructose/glucose 40 g/day; G80, fructose/glucose 80 g/day; G, glucose; S, sucrose; I, impaired glucose tolerance, N, normal glucose tolerance/body weight; O, overweight

Study or Subgroup	Mean Difference	SE	Weight	Mean Difference IV, Random, 95% CI
**10.2.1 BMI</= 25**				
Aeberli 2011 G40	-0.4	0.72	27.4%	-0.40 [-1.81, 1.01]
Aeberli 2011 G80	0.2	0.72	27.4%	0.20 [-1.21, 1.61]
Aeberli 2011 S80	-0.6	0.69	29.8%	-0.60 [-1.95, 0.75]
Aeberli 2013 G	-1.8	2.98	1.6%	-1.80 [-7.64, 4.04]
Aeberli 2013 S	-1.1	2.98	1.6%	-1.10 [-6.94, 4.74]
Heden 2014/1	-0.1	1.48	6.5%	-0.10 [-3.00, 2.80]
Heden 2014/3	-0.2	1.58	5.7%	-0.20 [-3.30, 2.90]
**Subtotal (95% CI)**			**100.0%**	**-0.30 [-1.04, 0.44]**
Heterogeneity: Tau^2 = 0.00; Chi^2 = 1.04, df = 6 (P = 0.98); I^2 = 0%				
Test for overall effect: Z = 0.79 (P = 0.43)				
**10.2.2 BMI 25.1-29.9**				
Angelopolous 2016	3.5	1.93	6.1%	3.50 [-0.28, 7.28]
Bantle 2000	0.1	0.18	33.5%	0.10 [-0.25, 0.45]
Kuzma 2019 All	-0.72	0.83	18.7%	-0.72 [-2.35, 0.91]
Lowndes 2015	-1.9	2.85	3.1%	-1.90 [-7.49, 3.69]
Malerbi 1996	-0.7	0.89	17.5%	-0.70 [-2.44, 1.04]
Silbernagel 2011	-1.9	0.72	21.1%	-1.90 [-3.31, -0.49]
**Subtotal (95% CI)**			**100.0%**	**-0.47 [-1.50, 0.56]**
Heterogeneity: Tau^2 = 0.80; Chi^2 = 12.29, df = 5 (P = 0.03); I^2 = 59%				
Test for overall effect: Z = 0.89 (P = 0.37)				
**10.2.3 30 and greater**				
Heden 2014/2	0.2	2.65	19.0%	0.20 [-4.99, 5.39]
Heden 2014/4	-0.9	2.04	32.1%	-0.90 [-4.90, 3.10]
Heden 2015	0	1.7	46.3%	0.00 [-3.33, 3.33]
Jin 2014	0.5	7.19	2.6%	0.50 [-13.59, 14.59]
**Subtotal (95% CI)**			**100.0%**	**-0.24 [-2.50, 2.03]**
Heterogeneity: Tau^2 = 0.00; Chi^2 = 0.16, df = 3 (P = 0.98); I^2 = 0%				
Test for overall effect: Z = 0.21 (P = 0.84)				

Test for subgroup differences: Chi^2 = 0.08, df = 2 (P = 0.96), I^2 = 0%

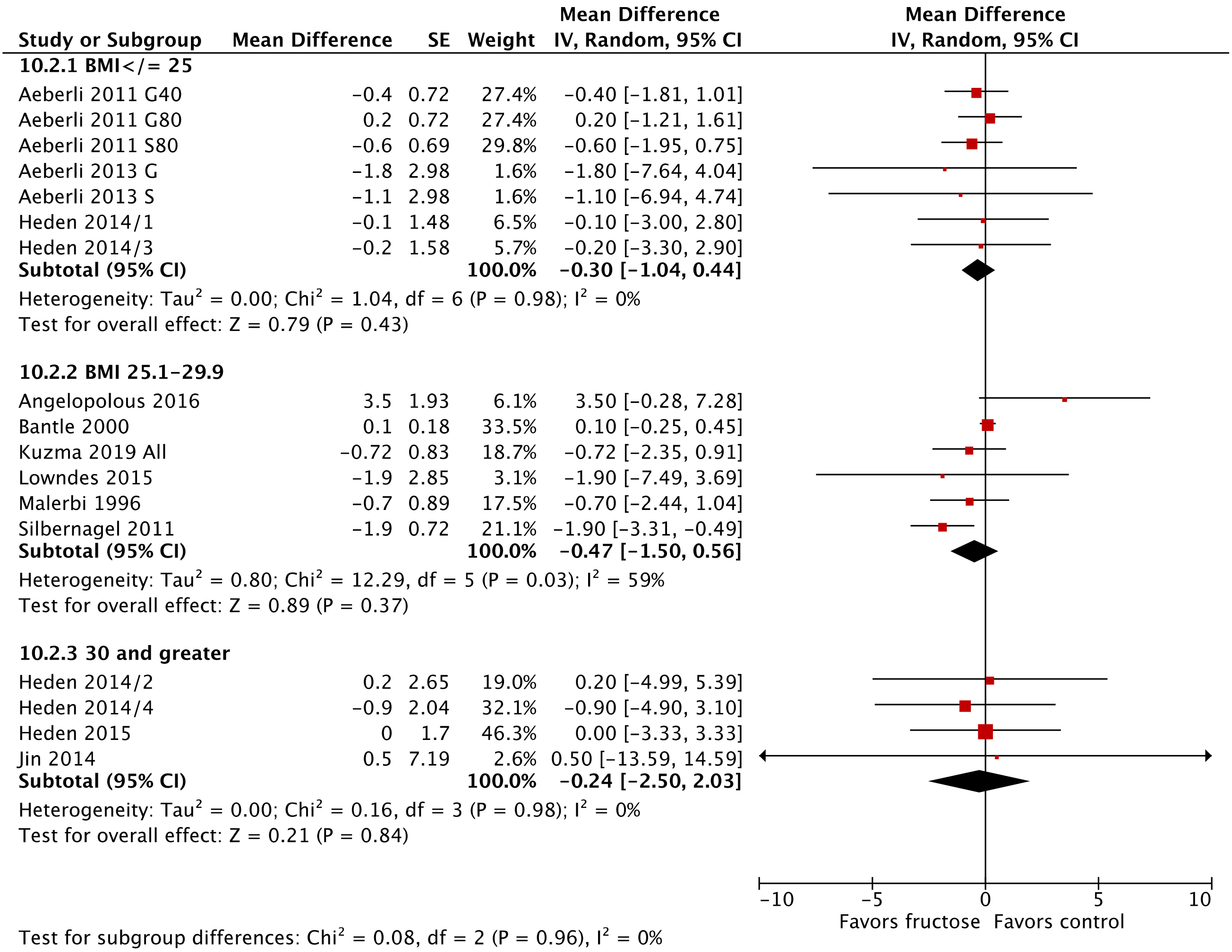

Supplementary figure 25: Subgroup meta-analysis of body weight following isoenergetic substitution of glucose or sucrose by fructose in food or beverages by baseline BMI. Values are mean differences [95% CIs] (expressed as kg) between body weight after fructose consumption and body weight following glucose or sucrose consumption. IV, inverse variance; SE, standard error; G40, fructose/glucose 40 g/day; G80, fructose/glucose 80 g/day; G, glucose; S, sucrose; I, impaired glucose tolerance, N, normal glucose tolerance/body weight; O, overweight

Study or Subgroup	Mean Difference	SE	Weight	Mean Difference IV, Random, 95% CI
**10.4.1 No diabetes**				
Aeberli 2011 G40	−0.4	0.72	4.5%	−0.40 [−1.81, 1.01]
Aeberli 2011 G80	0.2	0.72	4.5%	0.20 [−1.21, 1.61]
Aeberli 2011 S80	−0.6	0.69	5.0%	−0.60 [−1.95, 0.75]
Aeberli 2013 G	−1.8	2.98	0.3%	−1.80 [−7.64, 4.04]
Aeberli 2013 S	−1.1	2.98	0.3%	−1.10 [−6.94, 4.74]
Angelopolous 2016	3.5	1.93	0.6%	3.50 [−0.28, 7.28]
Bantle 2000	0.1	0.18	72.7%	0.10 [−0.25, 0.45]
Heden 2014/1	−0.1	1.48	1.1%	−0.10 [−3.00, 2.80]
Heden 2014/2	0.2	2.65	0.3%	0.20 [−4.99, 5.39]
Heden 2014/3	−0.2	1.58	0.9%	−0.20 [−3.30, 2.90]
Heden 2014/4	−0.9	2.04	0.6%	−0.90 [−4.90, 3.10]
Heden 2015	0	1.7	0.8%	0.00 [−3.33, 3.33]
Jin 2014	0.5	7.19	0.0%	0.50 [−13.59, 14.59]
Kuzma 2019 All	−0.72	0.83	3.4%	−0.72 [−2.35, 0.91]
Lowndes 2015	−1.9	2.85	0.3%	−1.90 [−7.49, 3.69]
Silbernagel 2011	−1.9	0.72	4.5%	−1.90 [−3.31, −0.49]
**Subtotal (95% CI)**			**100.0%**	**−0.08 [−0.38, 0.23]**
Heterogeneity: Tau^2 = 0.00; Chi^2 = 13.39, df = 15 (P = 0.57); I^2 = 0%				
Test for overall effect: Z = 0.49 (P = 0.62)				
**10.4.2 Impaired glucose tolerance**				
**Subtotal (95% CI)**				**Not estimable**
Heterogeneity: Not applicable				
Test for overall effect: Not applicable				
**10.4.3 Type 2 diabetes**				
Malerbi 1996	−0.7	0.89	100.0%	−0.70 [−2.44, 1.04]
**Subtotal (95% CI)**			**100.0%**	**−0.70 [−2.44, 1.04]**
Heterogeneity: Not applicable				
Test for overall effect: Z = 0.79 (P = 0.43)				
Test for subgroup differences: Chi^2 = 0.48, df = 1 (P = 0.49), I^2 = 0%				

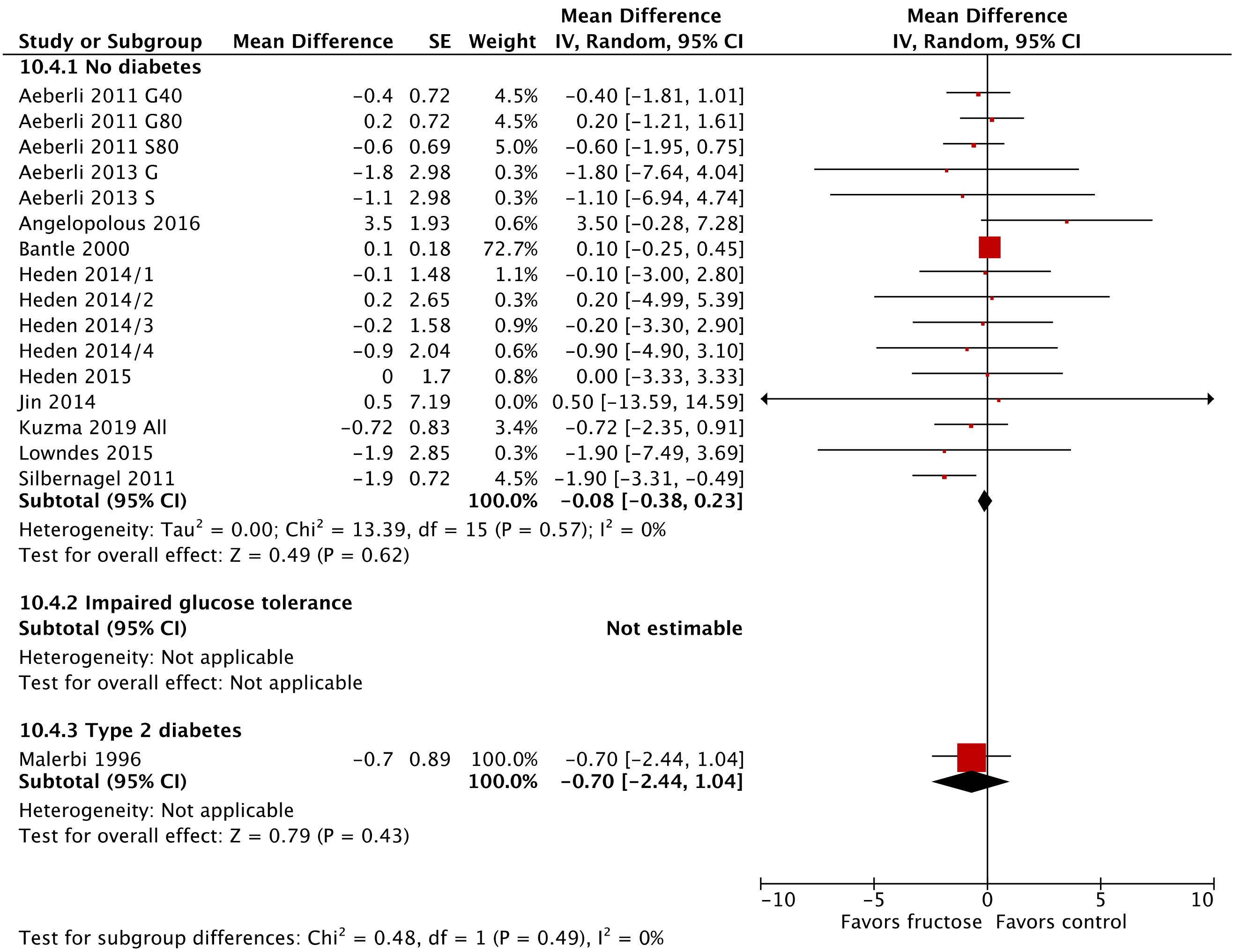

Supplementary figure 26: Subgroup meta-analysis of body weight following isoenergetic substitution of glucose or sucrose by fructose in food or beverages by diabetes status. Values are mean differences [95% CIs] (expressed as kg) between body weight after fructose consumption and body weight following glucose or sucrose consumption. IV, inverse variance; SE, standard error; G40, fructose/glucose 40 g/day; G80, fructose/glucose 80 g/day; G, glucose; S, sucrose; I, impaired glucose tolerance, N, normal glucose tolerance/body weight; O, overweight

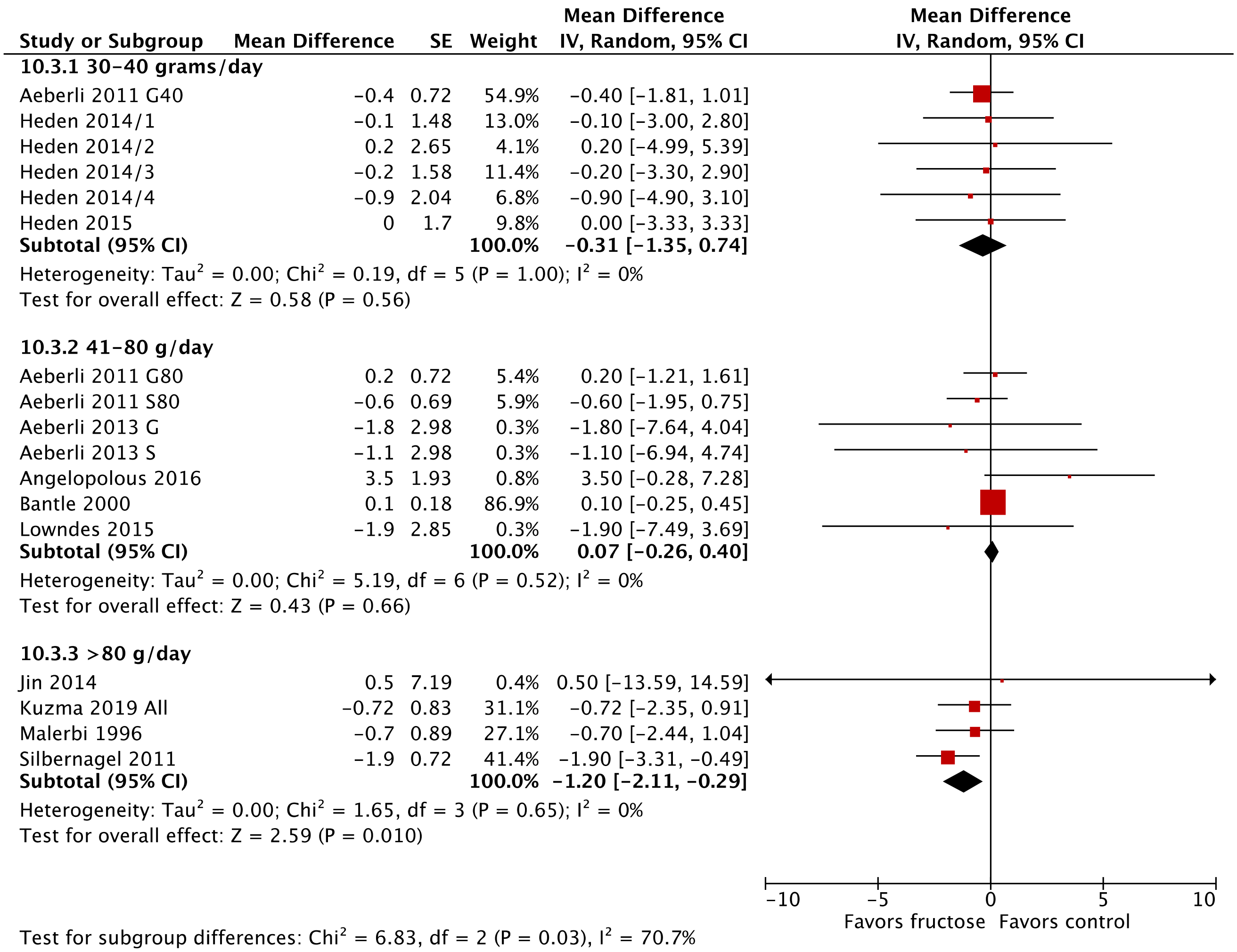

Supplementary figure 27: Subgroup meta-analysis of body weight following isoenergetic substitution of glucose or sucrose by fructose in food or beverages by dose of sugar. Values are mean differences [95% CIs] (expressed as kg) between body weight after fructose consumption and body weight following glucose or sucrose consumption. IV, inverse variance; SE, standard error; G40, fructose/glucose 40 g/day; G80, fructose/glucose 80 g/day; G, glucose; S, sucrose; I, impaired glucose tolerance, N, normal glucose tolerance/body weight; O, overweight

Supplementary table 1: meta-regression of studies reporting on fasting blood insulin

Potential covariant		N study arms	Coefficient	Lower bound	Upper bound	p value
**Factor covariates**						
Control sugar	glucose	13				
	sucrose	3	1.028	-1.543	3.699	0.433
Diabetes status	normoglycaemia	14				
	impaired glucose tolerance	1	-1.874	-4.890	1.142	0.223
	type 2 diabetes	1	-1.024	-5.015	2.967	0.615
Study type	cross-over	13				
	parallel	3	1.905	-1.323	5.133	0.247
Food vs beverage	beverage	12				
	food	4	-0.404	-2.426	1.618	0.695
Gender	males	4				
	both	10	-2.961	-4.109	-1.813	**<0.001**
	females	2	-2.239	-3.622	-0.856	**0.002**
Funding	government	14				
	both	1	-0.677	-4.651	3.297	0.739
	industry	1	4.263	-0.445	8.971	0.076
Blinding	Yes	11				
	Unclear	5	-2.587	-4.187	-0.987	**0.002**
**Continuous covariates**						
BMI	Range: 22.4 - 34.6	16	-0.061	-0.339	0.217	0.668
Age	Range: 13.5 - 54.2	16	-0.066	-0.125	-0.008	**0.026**
Dose	Range: 35 - 150	16	-0.005	-0.032	0.021	0.689
Year of publication	Range: 1984 - 2019	16	0.089	0.022	0.155	**0.009**

Supplementary table 2: meta-regression of studies reporting on body weight

Potential covariant		N study arms	Coefficient	Lower bound	Upper bound	p value
**Factor covariates**						
Control sugar	glucose	14				
	sucrose	3	-0.608	-1.703	0.488	0.277
Diabetes status	normoglycaemia	16				
	impaired glucose tolerance	0	N/A	N/A	N/A	N/A
	type 2 diabetes	1	-0.485	-2.345	1.376	0.610
Study type	cross-over	13				
	parallel	4	-1.233	-2.550	0.084	0.066
Food vs beverage	beverage	16				
	food	1	-0.485	-2.345	1.376	0.610
Gender	males	7				
	both	8	0.244	-0.576	1.064	0.560
	females	2	-0.169	-2.730	2.392	0.897
Funding	government	14				
	both	1	-0.416	-2.306	1.474	0.666
	industry	2	2.027	-1.121	5.266	0.203
Blinding	Yes	14				
	Unclear	3	0.628	-0.051	1.307	0.070
**Continuous covariates**						
BMI	Range: 22.4 - 34.6	17	-0.018	-0.226	0.189	0.862
Age	Range: 13.5 - 54.2	17	0.021	-0.016	0.058	0.270
Dose	Range: 35 - 150	17	-0.017	-0.031	-0.003	**0.016**
Year of publication	Range: 1996 - 2016	17	-0.039	-0.090	0.012	0.131

Supplementary table 2: meta-regression of studies reporting on fasting blood triglycerides

Potential covariant		N study arms	Coefficient	Lower bound	Upper bound	p value
**Factor covariates**						
Control sugar	glucose	12				
	sucrose	3	-0.096	-0.263	0.071	0.261
Diabetes status	normoglycaemia	13				
	impaired glucose tolerance	1	-0.252	-0.520	0.016	0.066
	type 2 diabetes	1	-0.142	-0.358	0.075	0.199
Study type	cross-over	12				
	parallel	3	-0.001	-0.235	0.233	0.995
Food vs beverage	beverage	11				
	food	4	-0.139	-0.273	-0.005	**0.042**
Gender	males	5				
	both	7	-0.200	-0.322	-0.078	**0.001**
	females	3	-0.165	-0.301	-0.030	**0.017**
Funding	government	13				
	both	1	-0.120	-0.360	0.120	0.328
	industry	1	0.060	-0.240	0.360	0.694
Blinding	Yes	9				
	Unclear	6	-0.031	-0.175	0.112	0.670
**Continuous covariates**						
BMI	Range: 22.4 - 32.6	15	-0.007	-0.033	0.019	0.609
Age	Range: 17.1 - 54	15	-0.001	-0.006	0.004	0.793
Dose	Range: 35 - 150	15	0.000	-0.003	0.002	0.802
Year of publication	Range: 1984 - 2016	15	0.003	-0.003	0.009	0.314
